# Neoadjuvant eribulin in HER2-negative early-stage breast cancer (SOLTI-1007-NeoEribulin): a multicenter, two-cohort, non-randomized phase II trial

**DOI:** 10.1038/s41523-021-00351-4

**Published:** 2021-11-25

**Authors:** Tomás Pascual, Mafalda Oliveira, Patricia Villagrasa, Vanesa Ortega, Laia Paré, Begoña Bermejo, Serafín Morales, Kepa Amillano, Rafael López, Patricia Galván, Jordi Canes, Fernando Salvador, Paolo Nuciforo, Isabel T. Rubio, Antonio Llombart-Cussac, Serena Di Cosimo, José Baselga, Nadia Harbeck, Aleix Prat, Javier Cortés

**Affiliations:** 1 SOLTI Breast Cancer Research Group, Barcelona, Spain; 2 Medical Oncology Department, Vall d’Hebron Institute of Oncology (VHIO), Hospital Universitari Vall d’Hebron, Vall d’Hebron Barcelona Hospital Campus, Barcelona, Spain; 3 Breast Cancer Program, Vall d’Hebron Institute of Oncology (VHIO), Hospital Universitari Vall d’Hebron, Vall d’Hebron Barcelona Hospital Campus, Barcelona, Spain; 4 Department of Medical Oncology, Hospital Clínico Universitario de Valencia, Valencia, Spain; 5 Department of Medical Oncology, Hospital Universitario Arnau de Vilanova, Lleida, Spain; 6 Department of Medical Oncology, Hospital Universitari Sant Joan, Reus, Spain; 7 Department of Medical Oncology, Hospital Clínico Universitario de Santiago, Santiago de Compostela, Spain; 8 Translational Genomics and Targeted Therapies in Solid Tumors, August Pi i Sunyer Biomedical Research Institute (IDIBAPS), Barcelona, Spain; 9 Molecular Oncology Lab, Vall d’Hebron Institute of Oncology, Barcelona, Spain; 10 Medical Oncology Department, Hospital Arnau de Vilanova, Fundación para el Fomento de la Investigación Sanitaria i Biomédica de la Comunidad Valenciana (FISABIO), Valencia, Spain; 11 Universidad Catolica de Valencia “San Vicente Martir”, Valencia, Spain; 12 Biomarkers Unit, Department of Applied Research and Technological Development, Fondazione IRCCS Istituto Nazionale dei Tumori, Milano, Italy; 13 Human Oncology & Pathogenesis Program (HOPP), Memorial Sloan Kettering Cancer Center, New York, NY USA; 14 Breast Center, Department OB&GYN and CCCLMU, LMU University Hospital, Munich, Germany; 15 Department of Medical Oncology, Hospital Clinic of Barcelona, Barcelona, Spain; 16 Department of Medicine, University of Barcelona, Barcelona, Spain; 17 International Breast Cancer Center (IBCC), Quiron Group, Barcelona, Spain; 18 Medica Scientia Innovation Research (MEDSIR), Barcelona, Spain

**Keywords:** Predictive markers, Breast cancer, Breast cancer, Breast cancer, Translational research

## Abstract

Eribulin prolongs overall survival in patients with pre-treated advanced breast cancer. However, no biomarker exists to prospectively select patients who will benefit the most from this drug. SOLTI-1007-NeoEribulin is a phase II, open-label, two-cohort, exploratory pharmacogenomic study in patients with clinical stage I–II HER2-negative breast cancer receiving neoadjuvant eribulin monotherapy treatment. Primary objective was to explore the association of baseline tumor gene expression with pathological complete response in the breast (pCR_B_) at surgery. Key secondary objectives were pCR_B_ rates in all patients and according to HR status, gene expression changes during treatment and safety. One-hundred one hormonal receptor-positive (HR + ) and seventy-three triple-negative breast cancer (TNBC) patients were recruited. The pCR_B_ rates were 6.4% in all patients, 4.9% in HR + disease and 8.2% in TNBC. The TNBC cohort was interrupted due to a progression disease rate of 30.1%. The pCR_B_ rates differed according to intrinsic subtypes: 28.6% in HER2-enriched, 11.1% in Normal-like, 7.9% in Luminal B, 5.9% in Basal-like and 0% in Luminal A (HER2-enriched vs. others odds ratio = 7.05, 95% CI 1.80–42.14; *p*-value = 0.032). Intrinsic subtype changes at surgery occurred in 33.3% of cases, mostly (49.0%) Luminal B converting to Luminal A or Basal-like converting to Normal-like. Baseline tumor-infiltrating lymphocytes (TILs) were significantly associated with pCR. Eribulin showed a good safety profile with a low response and pCR_B_ rates. Patients with HER2-negative disease with a HER2-enriched profile may benefit the most from eribulin. In addition, significant biological activity of eribulin is observed in Luminal B and Basal-like subtypes.

## Introduction

Eribulin is a non-taxane microtubule polymerization inhibitor recommended for advanced HER2-negative breast cancer progressing to 1–2 prior chemotherapeutic regimens, including anthracyclines and taxanes^1^. Approval of eribulin monotherapy was based on the results of the EMBRACE phase III trial^2^, which showed a statistically significant overall survival (OS) advantage vs. treatment of physician’s choice (13.1 vs. 10.6 months; hazard ratio 0.81, *p* = 0.041)^2^. However, no biomarker exists to date with the ability to predict benefit from eribulin within HER2-negative breast cancer.

SOLTI-1007 NeoEribulin (NCT01669252) is an open-label phase II pharmacogenomic study of single agent eribulin as neoadjuvant treatment for operable Stage I–II HER2-negative breast cancer. Cohort 1 included patients with triple-negative breast cancer (TNBC) and cohort 2 included patients with hormonal receptor (HR)-positive breast cancer. The main purpose of this study was to identify potential predictive biomarkers of eribulin efficacy in HER2-negative breast cancer.

## Results

### Patient characteristics

**Fig. 1:**
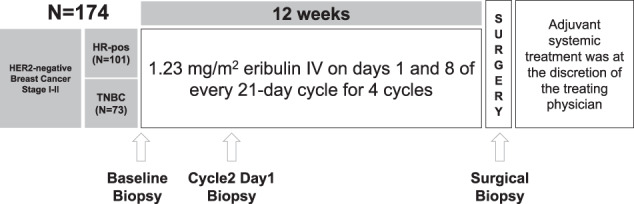
Trial profile. Schematic representation of the SOLTI-1007 NeoEribulin study.

**Fig. 2:**
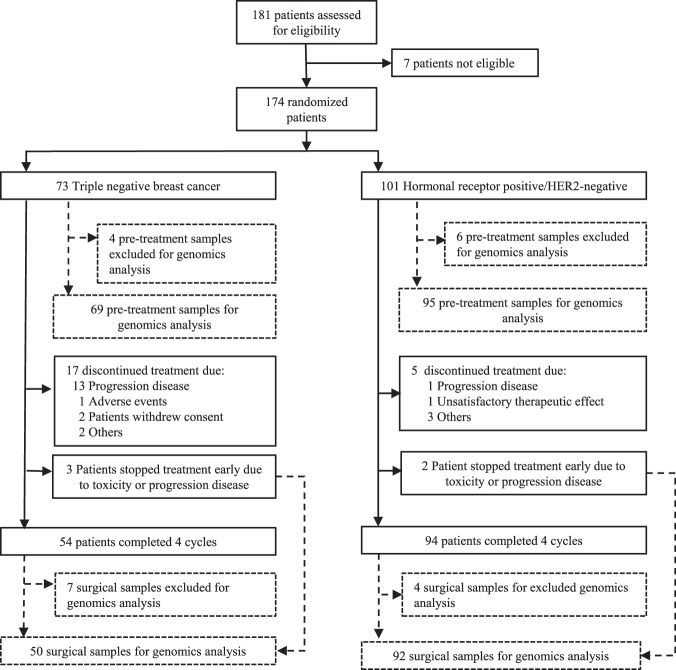
The CONSORT Flow Diagram. Flow chart of the SOLTI-1007 NeoEribulin study.

Between September 2012 and October 2015, 174 patients were enrolled (73 TNBC patients and 101 HR-positive patients) (Fig. 1). Eighteen patients had either insufficient biopsy material (*n* = 10) or withdraw from the trial (*n* = 8), leaving 156 patients as the evaluable population, including 65 patients with TNBC and 91 with HR-positive disease (Fig. 2). All patients (*n* = 174) were included in the intention to treat (ITT) and safety populations. A total of 148 of 174 (85.1%) patients completed neoadjuvant therapy and underwent definitive breast and axillary surgery. Regarding the main baseline clinic-pathological characteristics (Table 1), median age was 53 years (range 25–82), median tumor size was 30 mm (range 12–116), and most patients had T2 tumors (77.6%) and no axillary node involvement (71.8%).

**Table 1:** Patient baseline characteristics.

Characteristic, *n* (%)	TNBC *N* = 73	HR + /HER2– *N* = 101	Overall *N* = 174
	53 [33–82]	52.9 (25–80)	53 (25–82)
Median age (range)			
Sex			
Female	73 (100%)	98 (97.7%)	171 (98.28%)
Male	0	3 (2.3%)	3 (1.72%)
Menopausal status			
Pre-menopausal	25 (34.2%)	45 (44.6%)	70 (40.22%)
Postmenopausal	48 (65.8%)	53 (52.5%)	101 (58.0%)
Tumor size			
Median (range)	32 (13-114)	30 (12–116)	30 (12–116)
T1	6 (8.2%)	10 (9.9%)	16 (9.2%)
T2	64 (87.7%)	71 (70.3%)	135 (77.6%)
T3	3 (4.1%)	20 (19.8%)	23 (13.2%)
Lymph node status			
N0	57 (78.1%)	68 (67.3%)	125 (71.8%)
N1	16 (21.9%)	32 (31.7%)	48 (27.6%)
N2	0	1 (1%)	1 (0.6%)
Clinical baseline tumor stage			
I	7 (9.6%)	6 (5.9%)	13 (7.5%)
II	66 (90.4%)	91 (90.1%)	157 (90.2%)
III	0	4 (4%)	4 (2.3%)
Histological type			
Ductal	64 (87.7%)	78 (77.2%)	142 (81.6%)
Lobular	0	16 (15.8%)	16 (9.2%)
Other	9 (12.3%)	7 (6.9%)	16 (9.2%)
Histologic grade			
G1	2 (2.7%)	11 (10.9%)	13 (7.5%)
G2	26 (35.6%)	64 (63.4%)	90 (51.7%)
G3	45 (61.6%)	26 (25.7%)	71 (40.8%)
Ki67 expression (local)			
Ki67 mean (SD)	61.1 (24.9)	30.7 (21.1)	43.5 (27.3)
Ki67 median (range)	70 (5–95)	25(3–90)	35 (3–95)
≤14% *N* (%)	3 (4.1%)	21 (20.8%)	24 (13.8%)

### Pathological and radiological responses

A pathological complete response in the breast (pCR_B_) was noted in 11 of 174 women (pCR_B_ rate 6.4%, 95% confidence interval [CI] 3.2–11.0). In the TNBC group, the proportion of patients with a pCR_B,_ pathological complete response in the breast and axilla (pCR_BA_) and residual cancer burden (RCB) 0-1 at surgery were 8.2% (95% CI 3.1–17.0), 8.2% (95% CI 3.1–17.0) and 12.3% (95% CI 5.8–22.1) respectively (Table 2). In the HR-positive group, the proportion of patients at surgery with a pCR_B_, pCR_BA_ and RCB 0-1 were 4.9% (95% CI 1.6–11.2), 2.0% (95% CI 0.3–7.0) and 4.9% (95% CI 1.6–11.1), respectively. Age, HR status, tumor size, menopausal status, nodal status, ki67 score or histological grade were not found associated with pCR_B_, pCR_BA_ or RCB 0/1 (data not shown).

**Table 2:** Secondary endpoints determined at surgery.

		Triple negative		Hormone receptor-positive		Overall	
		*N* = 73		*N* = 101		*N* = 174	
		*n* (%)	95% IC	*n* (%)	95% IC	*n* (%)	95% IC
pCR_B_	Yes	6 (8.2)	3.1–17.0	5 (4.9)	1.6–11.2	11 (6.4)	3.2–11.0
pCR_BA_	Yes	6 (8.2)	3.1–17.0	2 (2.0)	0.3–7.0	8 (4.6)	2.0–8.9
Residual cancer burden	0–I	9 (12.3)	5.8–22.1	5 (4.9)	1.6–11.1	14 (8.0)	4.4–13.1
	II-III	55 (75.3)	63.8-84.7	87 (86.1)	77.8–92.2	142 (81.6)	75.0–87.1
	NA	9 (12.3)	-	9 (8.9)	-	18 (10.3)	-
Overall response rate	CR	4 (5.5)	1.5–13.5	4 (3.9)	1.1–9.8	8 (4.6)	2.0–8.9
	PR	18 (24.7)	15.3–36.2	44 (43.6)	33.8–53.8	62 (35.6)	28.5–43.2
	SD	23 (31.5)	21.1–43.4	41 (40.6)	30.9–50.8	64 (36.8)	29.6–44.4
	PD	22 (30.1)	19.9–42.0	8 (7.9)	3.4–14.86	30 (17.3)	12.0–23.7
	NA	3 (4.1)	-	4 (3.9)	-	7 (4.0)	-
BCS	Yes	40 (54.79)	42.7–66.5	54 (53.47)	43.3–93.5	94 (54.0)	46.3–61.6

*BCS* breast conservative surgery, *CR* complete response, *pCR*_*B*_ pathologic complete response in breast, *pCR*_*BA*_ pathologic complete response in breast and axilla, *PR* partial response, *SD* stable disease, *PD* progression disease.

The proportion of patients with TNBC and HR-positive disease achieving a radiological objective response according with ultrasound (US) or Magnetic Resonance Imaging (MRI) before surgery was 30.1% (95% CI 19.9–41.9) and 47.5% (95% CI 37.5–57.7), respectively. Thirty of 174 (17.3%) patients had progressive disease, 14 patients discontinued the treatment due to this reason and in 16 patients achieved a radiological progression disease in the US or MRI before surgery. Within the 30 patients with progression disease there were 22 (30.1%: 95% CI 42.7–66.5) patients with TNBC and 8 (7.9%: 95% CI 3.4–14.86) patients with HR-positive disease. The proportion of patients with TNBC and HR-positive disease who had breast conserving surgery was 54.8% (95% CI 42.7–66.5) and 53.5% (95% CI 43.3–93.5), respectively.

### Individual gene expression at baseline and eribulin response

**Fig. 3:**
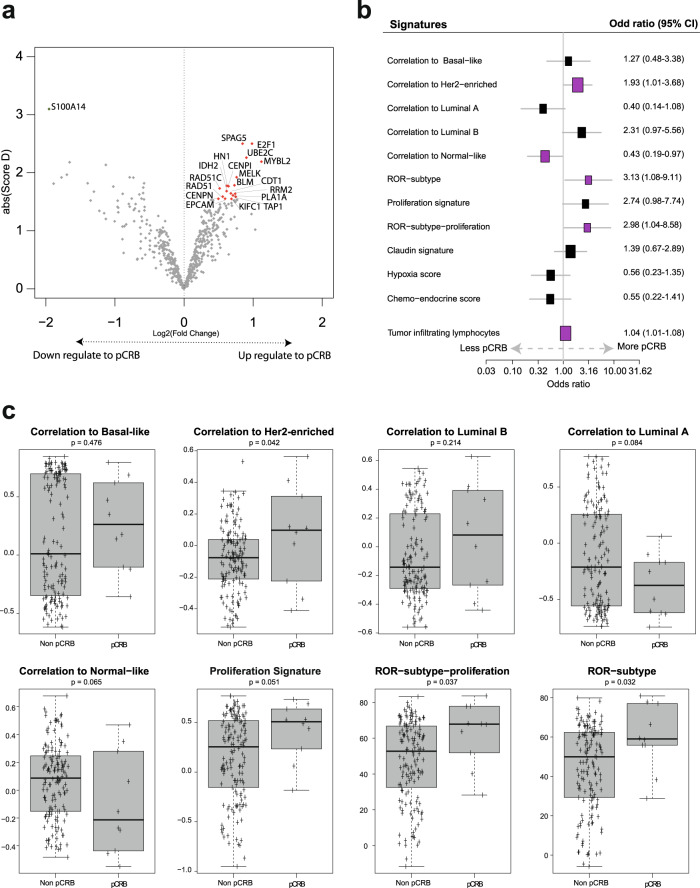
Treatment activity based on baseline gene expression. **a** Genes found differentially expressed in baseline samples between pCR_B_ and no-pCR_B_ in SAM analysis. Colored dots mark the genes with a false discovery rate (FDR) = 0, in red upregulate and in green downregulated. **b** Association of 11 gene signatures (as a continuous variable) with pCR adjusted for cohort (TNBC and HR-positive). Each signature was evaluated as a continuous variable and was standardized to have a mean of 0 and a SD of 1. The size of the square is inversely proportional to the standard error; horizontal bars represent the 95% confidence intervals (CI) of Odd ratios. Statistically significant variables are shown in pink. **c** PAM50 signature expression at baseline according with pathologic complete response (pCR) or residual disease (non-pCR) at surgery. *p*-value was obtained after performing ANOVA test. Error bars correspond to standard error of the mean.

The primary endpoint of the study was to identify individual genes whose expression at baseline is associated with eribulin response in HER2-negative disease. To accomplish it, expression of 540 breast cancer-related genes was evaluated in 156 baseline samples. In the evaluable population, expression of 19 individual genes (3.5%) was found significantly associated with pCR_B_ (false-discovery rate [FDR] < 1%) (Fig. 3a and Supplementary Table 1). Among them, high expression of proliferation-related genes such as *MELK*, *BLM*, *CENPN* and *E2F1* were associated with a higher likelihood of achieving a pCR_B_. On the contrary, high expression of *S100A14* was associated with residual disease at surgery (Supplementary Fig. 1). No gene was found associated with pCR_B_ in TNBC, and similar results as in the evaluable population were obtained in patients with HR-positive disease (data not shown).

### PAM50 intrinsic subtype at baseline and eribulin response

**Fig. 4:**
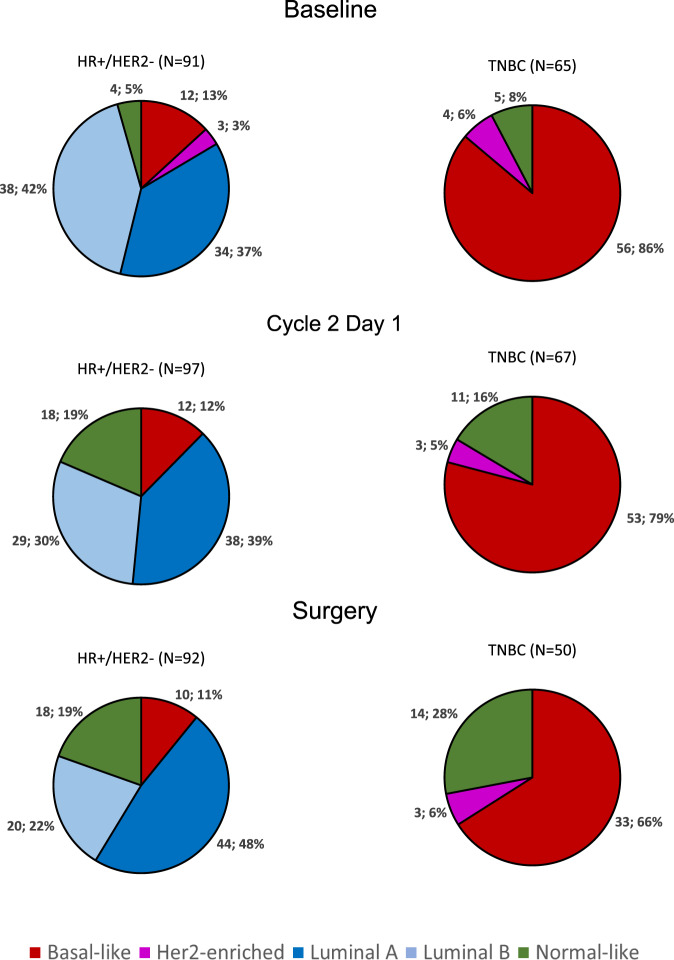
Intrinsic subtype distribution in according to hormonal receptor status at Baseline, C2D1 and surgery. HR: hormone receptor; TNBC triple-negative breast cancer.

All subtypes were identified and differed significantly by hormone receptor status (*p* < 0.001). In HR-positive disease (*n* = 91), the majority of tumors were identified as Luminal B (38 [41.8%]), followed by Luminal A (34 [37.4%]), Basal-like (12 [13.2%]), Normal-like (4 [4.4%]) and HER2-enriched (3 [3.3%]). In TNBC (*n* = 65), most tumors were identified as Basal-like (56 [86.2%]), followed Normal-like (5 [7.7%]) and HER-enriched (4 [6.1%]) (Fig. 4).

The highest rate of pCR_B_ was observed in the HER2-enriched subtype (2 of 7 [28.6%]), followed by Normal-like (1 of 9 [11.1%]), Luminal B (3 of 38 [7.9%]) (7.9%), Basal-like (4 of 68 [5.9%]) and Luminal A (0 of 34 [0%]). HER2-enriched tumors were found to be associated with higher pCR_B_ rates compared with non-HER2-enriched tumors (28.6% vs. 5.7%; odds ratio [OR] = 7.05, 95% CI 1.80–42.14, *p* = 0.032). When adjusted for HR status, the HER2-enriched vs. non-HER2-enriched OR was 6.78 (95% CI 1.12–40.98, *p* = 0.037). Of note, 30 patients (17.3%) presented progressive disease during therapy, including 8 patients with HR-positive disease and 22 patients with TNBC. Interestingly, 25 of 30 (83.3%) patients with progressive disease were identified as Basal-like, 2 (6.7%) patients as Luminal A, 1 (3.3%) patient as Luminal B and the other 2 (6.7%) patients did not have evaluable tumor sample.

### Gene signatures at baseline and eribulin response

To further explore the efficacy of eribulin based on baseline molecular features, we analyzed the following 11 signatures as a continuous variable: each of the 5 PAM50 subtype signatures^3^, the PAM50 proliferation signature^4^, two PAM50 risk of recurrence models (ROR) score^3,4^ (i.e., ROR-subtype and ROR-subtype-proliferation), the PAM50-based chemo-endocrine score^5^ (CES) and the hypoxia^6^ and claudin-low signatures^7^. High expression of 3 signatures (27.3%) was found associated with pCR_B_ after adjusting for HR status: the HER2-enriched signature (OR = 1.93; 95% CI 1.01-3.68), ROR-subtype (OR = 3.13; 95% CI 1.08-9.11) and ROR-subtype-proliferation (OR = 2.98; 95% CI 1.04-2.86). On the contrary, high expression of the Normal-like signature was found associated with residual disease after adjusting for HR status (OR = 0.43; 95% CI 0.19-0.97) (Fig. 3b, c).

### TILs at baseline and eribulin response

Tumor-infiltrating lymphocytes (TILs) were successfully evaluated in 156 out of the 176 (88.6%) baseline samples. The median TIL was 5% and most patients had TILs below 10% (interquartile range 3%–10%). TILs in TNBC were numerically higher compared with HR-positive disease (median 10% vs. 3%; *p* < 0.001) (Supplementary Fig. 2a). No statistically significant associations were identified between baseline TILs and age, nodal status, menopausal status and tumor stage. TILs varied statistically significantly according to the intrinsic subtype (*p* < 0.001), with the Basal-like subtype showing the highest score (median 10%), followed by the HER2-enriched (5%), Luminal B (3%) Normal-like (3%) and Luminal A (2%) (Supplementary Fig. 2b). Across HR-positive tumors, TILs also varied statistically significantly according to the intrinsic subtype (*p* < 0.001) (Supplementary Fig. 2c, d), with the higher levels of TILs in basal-like and HER2-enriched subtype.

We evaluated the association of baseline TILs with pCR. High TILs were statistically significantly associated with pCR after adjusting for HR status (OR = 1.04; 95% CI 1.01–1.08; *p* = 0.042) (Fig. 3b). The rates of pCR in ≤10% TILs and >10% were 4.1% and 12.9%, respectively (adjusted OR = 3.87, 95% CI 0.86–17.39, *p* = 0.076).

### Individual gene expression, intrinsic subtypes and gene signatures at C2D1, and eribulin response

**Fig. 5:**
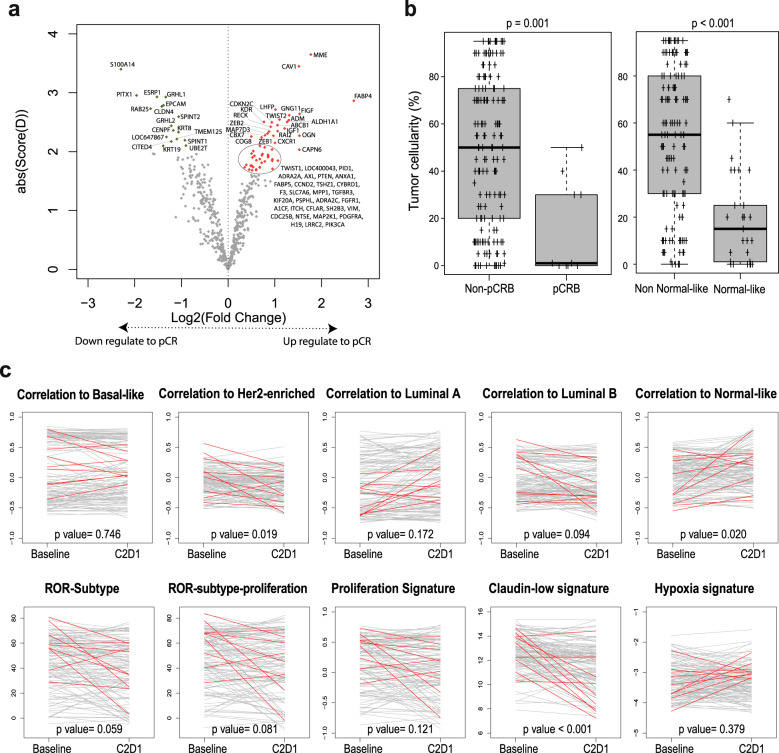
Treatment activity based on Cycle2 Day 1 (C2D1) gene expression. **a** Genes found differentially expressed in C2D1 samples between pCR_B_ and no-pCR_B_ in SAM analysis. Colored dots mark the genes with a false discovery rate (FDR) = 0, in red upregulate and in green downregulated. **b** Percentage of tumor cellularity at C2D1 according with pathologic complete response (pCR) or residual disease (non-pCR) at surgery and Normal-like subtype and non-normal-like subtype at C2D1. *p*-value was obtained after performing ANOVA test. **c** Variation of PAM50-based, claudin-low and hypoxia signatures (Baseline vs. C2D1). *p*-value was obtained after performing a paired *t*-test. Red lines mark the 9 patients, which achieve a pCR_B_ at surgery and with paired samples in both timepoints (Baseline vs. C2D1). Error bars correspond to standard error of the mean.

Gene expression was performed successfully in 164 samples obtained in cycle 2 day 1 (C2D1). As shown in Fig. 5a, the expression of 30 (5.5%) genes was found significantly associated with pCR_B_ (FDR < 1%) (Supplementary Table 2). Among them, high expression of mesenchymal-related genes such as *TWIST1/2, CAV1, FABP4* and *ZEB1/2* were associated with pCR_B_. On the contrary, high expression of epithelial genes such as *EPCAM, GRHL1* and *CLDN4* was associated with residual disease at surgery (Fig. 5a). Of note, the expression of S100A14 at baseline and C2D1 was associated with residual disease at surgery.

In patients with HR-positive disease, the majority of tumors at C2D1 were identified as Luminal A (38 [39.2%]), followed by Luminal B (29 [29.9%]), Normal-like (18[18.5%]) and Basal-like (12 [12.4%]). In patients with TNBC, most tumors at C2D1 were identified as Basal-like (53 [79.1%]), followed Normal-like (11[16.4%]) and HER2-enriched (3 [4.5%]) (Fig. 4).

At the time of surgery, pCR_B_ was noted in 5 of 29 patients (17.24%, 95% CI 5.85-35.77) who had Normal-like disease at C2D1 and 5 of 135 patients (3.70%, 95% CI 1.21-8.43) who had non-Normal-like disease at C2D1. The switch to the Normal-like subtype at C2D1 was likely reflecting early tumor response and increased proportion of normal breast tissue. Indeed, the mean tumor cellularity in Normal-like tumors at C2D1 was 19.0% (95% CI 11.60-26.40), compared with 54.0% (95% CI 48.78-59.22) in Non-normal-like tumors (Fig. 5b). On the other hand, the decreased expression of epithelial genes (Fig. 5a) the increase in mesenchymal genes at C2D1 could indicate an increase in mesenchymal cells in relation to tumor cells. When we performed the same analysis with the ratio of each gene between C2D1 and baseline, we observed the same signal; in other words, the increase in mesenchymal genes and the decrease in epithelial genes were associated with pCR_B_ at surgery (Supplementary Table 3) (Fig. 5c).

### TILs at C2D1 and eribulin response

A total of 146 (83.9%) tumor samples at D15 were available, and 134 (77.0%) tumor samples had paired baseline TILs data. Compared with baseline samples, levels of TILs at C2D1 were statistically significantly higher (mean difference +3.18%, 95% CI 0.76–5.60, *p* = 0.014). Across intrinsic subtypes the increase was statistical significant in basal-like and luminal B tumors (Supplementary Fig. 3). We evaluated TILs at C2D1 with pCR. In univariate analysis, high TILs were not statistically significantly associated with pCR (OR = 1.02; 95% CI 0.97–1.06; *p* = 0.426). Finally, we explored the absolute change and the ratio from baseline to D15 time points. Neither the absolute change nor the ratio were significantly associated with pCR.

### Intrinsic subtype and gene expression changes between baseline, C2D1 and surgery

**Fig. 6:**
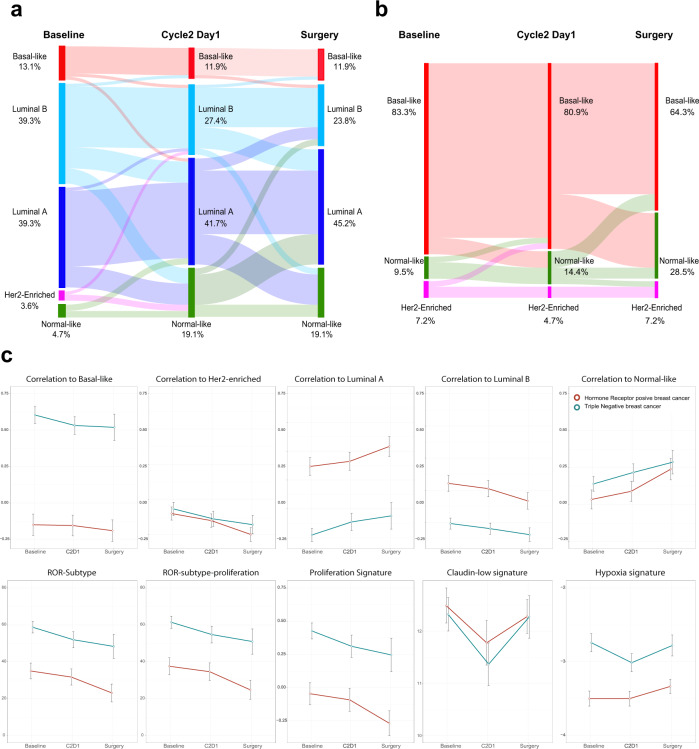
Breast cancer molecular subtype shifts between baseline, cycle 2 day 1 and surgery paired samples. **a** Hormone receptor positives tumors. **b** Triple-negative tumors, **c** Variation of PAM50-based, claudin-low and hypoxia signatures between baseline, Cycle 2 Day 1 (C2D1) and surgery in hormone receptor-positive and triple-negative breast cancer. Error bars correspond to standard error of the mean.

Intrinsic subtype at baseline, C2D1 and surgery was successfully identified in 132 patients (90 with HR-positive disease and 42 with TNBC). In patients with HR-positive disease, 33 (36.7%) of 90 patients had switched subtypes by C2D1, and his proportion was greatest in patients with Luminal B disease at baseline (Fig. 6a). At surgery, 44 (48.8%) of 90 patients had switched subtypes from baseline and were identified as Luminal A (43 [45.2%]), followed by Luminal B (20 [23.8%]), Normal-like (17 [19.1%]) and Basal-like (10 [11.9%]). In patients with TNBC, 5 (11.9%) of 42 patients had switched subtypes by C2D1 (Fig. 6b). At the time of surgery, 10 (48.8%) of 42 patients had switched subtypes from baseline and were identified as Basal-like (27 [64.3%]), followed by Normal-like (12 [28.5%]) and HER2-enriched (3 [7.2%]). As expected, Luminal A and Normal-like signatures were enriched at C2D1 and surgery in both cohorts of patients, whereas the other PAM50 subtypes and signatures, including the proliferation signature, were downregulated during eribulin therapy (Fig. 6c and Supplementary Fig. 4).

Finally, to identify genes whose expression changed between baseline and surgical specimens, we performed a paired two-class SAM analysis. Compared to baseline samples, 73 (13.5%) and 145 (26.8%) genes were found over- and under expressed in surgical specimens, respectively (FDR < 1%). Among them, we observed an increase in luminal-related genes (e.g., *ESR1* and *NAT1*), negative regulation of apoptosis (e.g., *BCL2* and *IL6*) and angiogenesis (e.g., *ANGPTL4*, *HIF1A*) and a decrease in the expression of cell cycle-related genes (e.g., *CCNB1*, *RAD17* and *MKI67*) and genes related to microtubule cytoskeleton organization (e.g., *AURKA, CENPA* and *KIF23*) (Supplementary Table 4).

### Safety

Eribulin administered during the preoperative setting was generally well tolerated. The most frequent grade 1–2 adverse events were alopecia (122 [70.1%]), asthenia (90 [51.7%]), and nausea (40 [23%]) Nearly all of the most frequent adverse events were deemed possibly related to study treatment (Table 3). Grade 3–4 toxicities were observed in 34 (19.6%) of patients. The most common grade 3–4 adverse events were neutropenia (6 [3.4%]) and increased in liver enzymes (3 [1.7%]). A total of 21 (12.1%) patients required a dose reduction or temporary interruption. The most common reasons for dose modifications were hematological toxicities (3.5%, *n* = 6), followed by non-hematological toxicities (2.3%, *n* = 4). A total of 3 (1.7%) patients discontinued permanently study treatment because of adverse events. No deaths were observed during the study.

**Table 3:** Summary of adverse events regardless of relationship to study drugs.

	Grade 1	Grade 2	Grade 3	Grade 4	Any Grade
	*n* (%)	*n* (%)	*n* (%)	*n* (%)	*n* (%)
Any adverse event^a^	157 (90.2%)	87 (50%)	32 (18.4%)	2 (1.2%)	159 (91.4%)
Alopecia	83 (47.7%)	32 (18.4%)	NA	NA	115 (79.9%)
Asthenia	70 (40.2%)	19 (10.9%)	1 (0.6%)	0	90 (51.7%)
Nausea	35 (20.1%)	3 (1.7%)	1 (0.6%)	0	40 (23.0%)
Mucosal inflammation	23 (13.2%)	2 (1.1%)	0	0	25 (14.4%)
Diarrhea	14 (8.0%)	5 (2.9%)	2 (1.1%)	0	21 (12.1%)
Neutropenia^b^	6 (3.4%)	1 (0.6%)	6 (3.4%)	1 (0.6%)	14 (8.0%)
Alanine aminotransferase increased	6 (3.4%)	4 (2.3%)	3 (1.7%)	0	13 (7.5%)
Paresthesia	9 (5.2%)	2 (1.1%)	1 (0.6%)	0	12 (6.9%)
Fatigue	7 (4.0%)	3 (1.7%)	1 (0.6%)	0	11 (6.4%)
Aspartate aminotransferase increased	6 (3.4%)	2 (1.1%)	3 (1.7%)	0	11 (6.4%)
Hyperglycemia	1 (0.6%)	2 (1.1%)	3 (1.7%)	0	6 (3.4%)
Gamma-glutamyltransferase increased	0	0	3 (1.7%)	0	3 (1.7%)
Febrile neutropenia	0	0	1 (0.6%)	1 (0.6%)	2 (1.1%)
Mastitis	0	1 (0.6%)	1 (0.6%)	0	2 (1.1%)
Post procedural hematoma	0	0	2 (1.1%)	0	2 (1.1%)
Blood alkaline phosphatase increased	1 (0.6%)	0	1 (0.6%)	0	2 (1.1%)
Blood bilirubin increased	1 (0.6%)	0	1 (0.6%)	0	2 (1.1%)
Transaminases increased	1 (0.6%)	0	1 (0.6%)	0	2 (1.1%)
Aphonia	1 (0.6%)	0	1 (0.6%)	0	2 (1.1%)
Thrombocytopenia^c^	0	0	1 (0.6%)	0	1 (0.6%)
Gait disturbance	0	0	1 (0.6%)	0	1 (0.6%)
Biliary colic	0	0	1 (0.6%)	0	1 (0.6%)
Drug hypersensitivity	0	0	1 (0.6%)	0	1 (0.6%)
Cellulitis	0	0	1 (0.6%)	0	1 (0.6%)
Hypokalaemia	0	0	1 (0.6%)	0	1 (0.6%)
Cerebrovascular accident	0	0	0	1 (0.6%)	1 (0.6%)
Dysarthria	0	0	1 (0.6%)	0	1 (0.6%)
Psychogenic tremor	0	0	1 (0.6%)	0	1 (0.6%)

Listed are all grade 3 and 4 events and grade 1–2 events that were reported in at least 10% of the patients. Grade 3 and 4 alopecia were not included in the National Cancer Institute Common Terminology Criteria for Adverse Events, version 4.03. *NA* = not applicable.

^a^Patients could have more than one adverse event.

^b^Neutropenia includes decreased neutrophil count.

^c^Thrombocytopenia includes platelet count decreased.

## Discussion

Eribulin is the only chemotherapeutic that has demonstrated a significant prolongation in overall survival on previously treated breast cancer patients. To date, no biomarker exists to prospectively select patients who will derive the maximum benefit from this agent. Our study describes a framework for assessing the biology behind this non-taxane microtubule dynamics inhibitor in HER2-negative primary breast cancer. Gene expression of 156 patients treated with eribulin and its correlation with efficacy suggest that single genes and genes signatures as molecular subtypes are modulated and play a role in responses to eribulin.

The results reported here demonstrate that the administration of 4 cycles of neoadjuvant eribulin was feasible and relatively well tolerated. Discouragingly, the pCR_B_ achieved in our study was low (i.e., 6.4% of the ITT population). Although the pCR_B_ in HR-positive is similar to other neoadjuvant taxane-based studies^8^, it is important to remark that the overall pCR in TNBC, who are considered more chemosensitive, was also relatively low. For example, pCR in TNBC after neoadjuvant weekly paclitaxel is around 20%^9,10^. Furthermore, disease progression rates in other neoadjuvant studies^8,11^ and historical results^12^ are below 5% in both HR-positive and TNBC, and radiological response are greater than 50%^8,11^. In our study, we observed a progression rate of 30.1% and 7.9% in the TNBC and HR-positive cohorts, respectively.

Recently, three further studies have assessed the administration of eribulin in early HER2-negative breast cancer. A recent study comparing Eribulin/Cyclophosphamide vs. Docetaxel/Cyclophosphamide in the neoadjuvant setting resulted in similar pCR rates^13^. The neoadjuvant HOPE trial^14^ failed to demonstrate high eribulin efficacy, as measured by pCR, in patients with TNBC treated with anthracycline and taxane. Moreover*,* Yardley et al. studied the role of additional eribulin as post-neoadjuvant in a phase II trial in breast cancer patients with residual invasive disease after taxane and/or anthracycline-based neoadjuvant therapy^15^. The disease-free survival results suggested a lack of benefit of adjuvant eribulin and did not reach the targeted endpoints of the study in any breast cancer subtype. Despite these results, eribulin is still an attractive drug because its toxicity profile compares favorably with taxanes, and avoids the risk of late toxicities associated with anthracyclines. Therefore, eribulin treatment in breast cancer setting could be evaluated in case of intolerance or contraindication to standard conventional chemotherapy regimens. These data also reinforce the need to focus on the identification of specific molecularly defined patient subsets and confirm whether gene expression signatures, as the obtained in NeoEribulin trial, could be applied to predict response to eribulin vs. standar chemotherapy.

The primary endpoint of our study was to evaluate whether the expression of a particular set of mRNAs in pre-treated tumors was associated with pCR_B_ after eribulin treatment. Among the 540 mRNAs evaluated, we identified the expression of 18 genes significantly associated with pCR_B_. Among them, proliferation genes such as *MELK* and *BLM*, and cell cycles genes such as *CENPN* and *E2F1*, were associated with a higher likelihood of achieving a pCR_B_. Interestingly, eight of theses transcripts (*MYBL2*, *E2F1*, *UBE2C*, *SPAG5*, *MELK*, *TAP1*, *RRM2*, *BLM*) lie downstream of p53, since they are regulated by the p53-DREAM pathway. The DREAM complex controls more than 250 genes, mostly associated with cell cycle^16^. In agreement with that, p53 defects have been associated with positive response to paclitaxel in neoadjuvant setting^17^. On the other hand, the expression of *S100A14* was associated with residual disease at surgery after eribulin. The S100A14 family of proteins present different role depending on tumor type^18^. Consistent with our results, in breast cancer the expression of *S100A14* provides poor prognosis in breast cancer patients^19^. One of the mechanisms by which S100 may promote breast cancer poor prognosis and progression is interacting with actin^19^. This interaction with cytoskeleton dynamics might confer resistance to eribulin.

Despite the low response, the pCR rates differed by subtype. The pCR rates of Luminal A, Luminal B, HER2-enriched and Basal-like were 0%, 7.9%, 28.6% and 5.9%, respectively. This difference was found statistically significant and independent of HR status. Interestingly, the HER2-enriched subtype is a consistent biomarker to identify patients with a higher likelihood of achieving a pCR following cytotoxic therapy^20^. Taking all these findings and observations in consideration, we may speculate about the use of this gene signature as a potential predictive tool for response to eribulin. However, future studies are needed to address this hypothesis.

Eribulin also exhibits non-mitotic activity including vascular remodeling, reversal of the epithelial-to-mesenchymal phenotype and suppression of cancer cell migration, invasion and experimental metastasis. One of the effects it is to induce a less agressive phenotype. In our study, as in other studies^21–23^, the surgical samples reveal that most residual specimens are classified as Luminal A or Normal-like. 44.44% of subtype changes in luminal B disease changed to luminal A. Luminal A tumores are more endocrine responsive and less proliferative than Lumninal B subtypes. The presence of Luminal A tumors fits with the increased expression of endocrine-sensitive and decreased expression of proliferation-related genes supporting the induction of a less aggressive phenotype after eribulin treatment. Whether this is due to changes in the biology of tumor cells at baseline or to selection of clones by eribulin, it cannot be addressed by our study and remains unknown. Interestingly, one future implication could be that eribulin treatment may trigger increased hormonal sensitivity in Luminal B tumors providing a rationale for combining eribulin with hormone therapies and a potential explanation of eribulin pretreatment role in sensitizing breast cancer to subsequent therapies. This hypothesis is being addressed in the currently REVERT clinical study (NCT03795012).

In our study, continuous TILs were associated with pCR, independently of the HR status. Interestingly, TILs varied significantly across different intrinsic subtypes in HR-positive tumors, with the highest levels within the non-luminal subtype (i.e., Basal-like and HER2-enriched). This finding has also been recently described by other groups^24,25^. Denkert et al.^26^ has revealed that there is a clear relationship between the number of TILs and pCR in breast cancer treated with neoadjuvant chemotherapy, but in HR-positive/HER2-negative disease, this has translated into shorter overall survival. One possibility is that the HR-positive/HER2-negative tumors with higher immune infiltration at baseline are enriched in non-luminal tumors, explaining their better response to neoadjuvant chemotherapy and less favorable survival.

Our study has several limitations worth noting. First, it is a single-arm, open-label study without a comparator arm. Second, the trial did not recruit the initially expected 100 TNBC patients and the overall pCR_B_ (6.4%) was lower than the assumed in the sample size calculation (15%). For those reasons, the trial did not achieve the planned statistical power for all comparisons and this fact limits the final conclusions. Third, the genomic correlative analyses did not include all specimens of the study (5.7% of pretreatment samples were not evaluable). Fourth, the panel under investigation was limited to 540 genes. Therefore, we were limited regarding the ability to derive new gene signatures and identify new biological processes associated with treatment response and interactions among genomic markers in predicting pCR could not be fully studied. Fifth, the trial ended after surgery; thus, no long-term follow-up is available and the use of standard adjuvant chemotherapy was left at the discretion of the physician.

In conclusion, eribulin showed a good safety profile with a low response rate in breast cancer. From a response and biological perspective, patients with HER2-enriched disease may benefit the most from eribulin therapy. Moreover, the 49% of subtype changes in luminal B disease changed to luminal A. This result suggests that future strategies combining eribulin with endocrine therapy could be reasonable in Luminal B breast cancer. These results support the notion that eribulin improves response to subsequent lines of therapy, including endocrine-based treatments.

## Methods

### Study design and participants

SOLTI-1007 NeoEribulin is an open-label, two-cohort, conducted in 3 countries at 30 trial centers, phase 2 pharmacogenomic study of single agent eribulin as neoadjuvant treatment for operable Stage I–II HER2-negative breast cancer. Cohort 1 included patients with TNBC and cohort 2 included HR-positive breast cancer.

Patients aged at least 18 years were eligible if they had previously untreated, locally confirmed HER2-negative, stage I–II invasive breast cancer (regardless of hormone receptor status), with primary tumors at least 2 cm in diameter (as measured by ultrasound or MRI), nodal status of 0–2, and no evidence of distant metastasis. Patients had to meet the minimum tissue requirement for gene expression analysis (≥10% invasive tumor cells and >4 mm^2^ tumor surface area). Patients also had to have an Eastern Cooperative Oncology Group (ECOG) performance status of 0–1, and adequate hematological counts and hepatic and renal function. Patients were excluded if they had multicentric tumors, stage III or IV disease, bilateral breast cancer, other malignancies, inadequate bone marrow or renal function, impaired liver or cardiac function, clinically significant cardiovascular disease, and uncontrolled infection.

The study was done in accordance with Good Clinical Practice guidelines and the Declaration of Helsinki. The study protocol was approved by independent ethics committees at each center and the “Agencia Española de Medicamentos y Productos Sanitarios (AEMPS)”. All patients provided written informed consent.

### Procedures

All patients received 4 cycles weeks of eribulin (1.23 mg/kg on Days 1 and 8 of every 21-day cycle). Dose reduction of eribulin to 0.97 mg/kg and 0.62 mg/kg was permitted for patients who developed grade 3 or 4 hematological or non-hematological adverse events and/or omitted of day 8 administration in previous cycle for toxicity. Treatment could be discontinued for a maximum of 14 days and could be resumed if adverse events resolved to grade 1 or below. We did safety laboratory testing (blood count and chemistry) at baseline and day 1 and 8 of every treatment cycle (3 weeks).

At Cycle 2 Day1 (±5 days), a core-needle biopsy was mandatory. Surgery was performed between 2 and 5 weeks after the last dose of eribulin. Standard adjuvant chemotherapy was administered according to the physician’s discretion. The last safety follow-up visit was done 30 days after surgery.

### Study endpoints

The primary endpoint of the trial was the correlation of pretreatment expression of mRNA from primary breast tumors with pathological complete response in the breast (pCR_B_)—defined as the absence of invasive neoplastic cells at microscopic examination of the primary tumor—at the time of surgery. Remaining in-situ lesions were allowed.

The seven key secondary endpoints that we report in this article are: (1) proportion of patients with a pCR_B_ (ypT0/Tis ypNx) and pCR in breast and axillary lymph nodes (pCR_BA_); defined as ypT0/Tis ypN0).(2) proportion of patients who had an objective response (defined as the sum of partial responses and complete responses according to Response Evaluation Criteria in Solid Tumors, version 1.1) (3) proportion of patients with a residual cancer burden score (RCB) 0-1; (4) frequency of breast conserving surgery; (5) safety and tolerability of treatment (6) proportion of pCR_B_ according to breast intrinsic cancer subtype (7) proportion of subtype switching from baseline to Cycle2 Day1 and surgery. Other secondary endpoints will be reported elsewhere. In a post-hoc exploratory analysis, TILs in baseline and C2D1 samples were correlated with pCR_B_.

The tumor-evaluable population was defined as all patients who had initial tumor biopsy mRNA in good condition for molecular analyses and who underwent definitive surgery or discontinue study treatment due to PD or toxicity. This population was used for the primary and genomic analysis. The ITT analysis population was defined as all patients with an enrollment date. This population were used for the analysis of secondary endpoints. The safety population included the set of patients who received at least one (even incomplete) dose of the study treatment. This population were used for the safety analysis.

### Efficacy assessments

Owing to the lack of neoadjuvant data on the proposed regimen, the trial included an interim analysis after the first 50 patients were enrolled. If a rate of progression disease (PD) of >15% had been observed, then the trial had been permanently stopped. The pre-planned efficacy interim analysis was evaluated by the Study Steering Committee, which deemed the trial efficacy and supported its continuation to full recruitment. After the trial met the criteria for continuation and the HR-positive cohort was complete, the sponsor stopped the enrollment in this study due al number of PD observed in the TNBC cohort.

### Gene expression analysis

At baseline, a section of the formalin-fixed paraffin-embedded (FFPE) breast tissue was examined with haematoxylin and eosin (H&E) staining to confirm the presence of invasive tumor cells and to determine the minimum tumor surface area. For samples obtained at Cycle 2 Day 1, those without invasive tumor cells were also profiled. For RNA purification (High Pure FFPET RNA isolation kit, Roche, Indianapolis, IN, USA), at least 1–5 10 μm FFPE slides were used for each tumor specimen, and macrodissection was performed (when needed) to avoid contamination with normal breast tissues. A minimum of roughly 100 ng of total RNA was used to measure the expression of 540 genes and the five housekeeping genes (nCounter platform, Nanostring Technologies, Seattle, WA, USA). Data were log2 transformed and normalized using the housekeeping genes.

Intrinsic molecular subtypes obtained at baseline, C2D1 and surgery were determined with the research-based PAM50 predictor as previously described^3,27,28^. For each sample, we calculated 11 gene signatures: the correlation coefficient to the 5 PAM50 centroids (basal-like, HER2-enriched, luminal A, luminal B and normal-like signatures, respectively)^3^, the PAM50 proliferation signature^4^, two PAM50 risk of recurrence models^3,4^ (ROR-subtype and ROR-subtype-proliferation), the Chemo-Endocrine Score^5^, Claudin-low signature^7^ and VEGF/Hypoxia signature^6^. The PAM 50 ROR models were calculated using weighted coefficients to the four subtypes and a proliferation score using a previously reported and validated formula^3,29^. ROR-subtype-proliferation was evaluated as a continuous variable, and as group categories using the previously reported cutoffs^29^.

### TILs and tumor cellularity

Histopathologic analysis of the proportion of TILs was done in whole sections of tumor tissue stained with H&E. TILs were quantified according to the 2014 Guidelines developed by the International TILs Working Group^30^. Percentages of TILs and tumor cellularity at baseline and C2D1 were scored in slides of core biopsies from clinic–pathologic and outcome data.

### Sample size calculation and statistical analysis

This trial was an exploratory study and sample size was not based on statistical power. Assuming an average pCR_B_ of 15% based on the literature^31^, a sample size of 200 patients, 100 TNBC patients and 100 HR-positive, was estimated to provide a 90% probability of detecting a gene signature whose expression is so associated with a two-fold increase in odds of achieving a pCR_B_, assuming 5% of patients were lost to follow-up and 5% had insufficient quality or quantity of RNA.

To compare distribution of variables between two groups, we used Fisher’s exact test. Proportions and 95% CIs were also provided. To identify genes whose expression was significantly different between paired pCR vs. no pCR, we used an unpaired two-class significance of microarrays (SAM) with a False Discovery Rate (FDR) = 5%^32^. Univariate and multivariate logistic regression analyses were done to investigate the association of each variable with pCR_B_. Odds ratios (ORs) and 95% confidence intervals (CI) were calculated for each variable. The significance level was set to a two-sided α of 0.05. We used R version 3.5.1 for all the statistical analyses.

### Reporting summary

Further information on research design is available in the Nature Research Reporting Summary linked to this article.

## Supplementary information

Supplementary Information

Reporting Summary

## Data Availability

The datasets used and/or analyzed that support the findings of this study have been deposited in “Gene Expression Omnibus” (“GEO”) with the accession code GSE186102.

## Code availability

All analyses were performed with R version 3.6.1 and Microsoft Excel. R codes are available from the corresponding author on reasonable request.
