# Supplementary material for: Neoadjuvant eribulin in HER2-negative early-stage breast cancer (SOLTI-1007-NeoEribulin): a multicenter, two-cohort, non-randomized phase II trial
Source: NPJ Breast Cancer. 2021 Nov 25;7:145. doi: 10.1038/s41523-021-00351-4 (PMC8616926; doi:10.1038/s41523-021-00351-4)
Supplement: Supplementary file 1 — Supplementary Information [file 41523_2021_351_MOESM1_ESM.pdf]

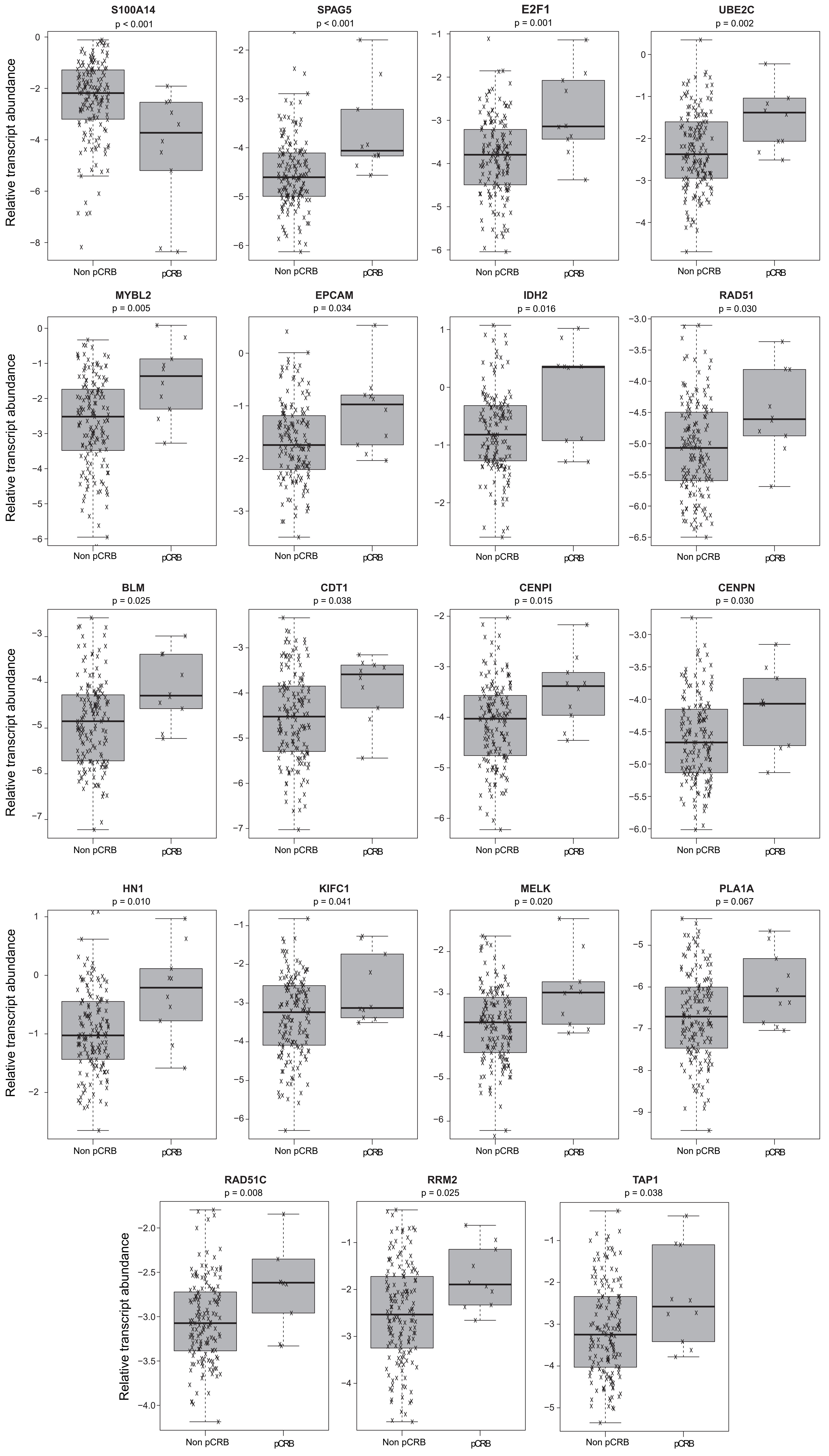

Supplementary Figure 1. Expression of the expression of 18 and 1 genes was found significantly associated with pathological complete response in breast (pCRB) and residual disease in baseline samples. Error bars correspond to standard error of the mean.

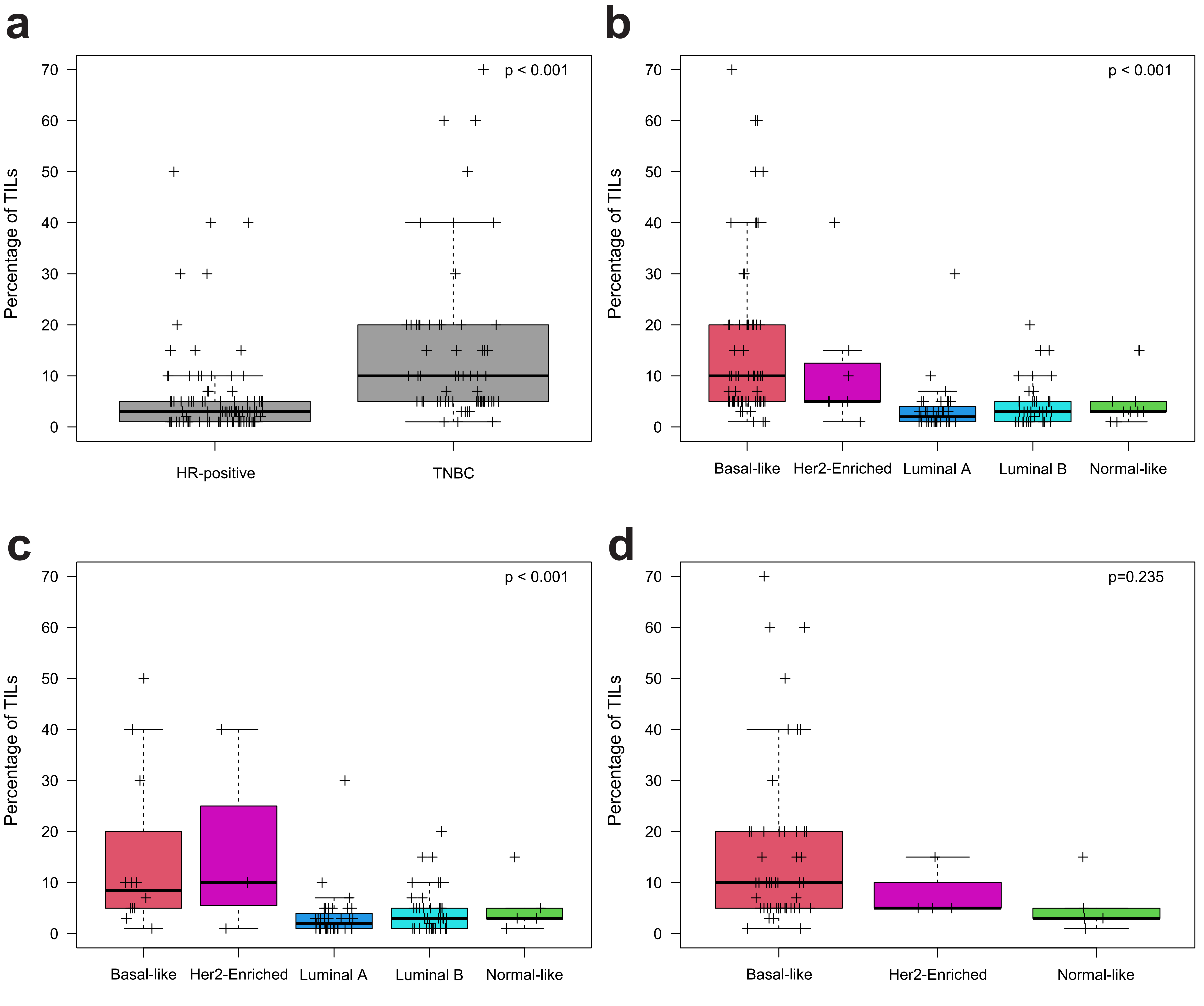

Supplementary Figure 2. Distribution of stromal TILS in NEOERIBULIN baseline primary breast cancer. a) according with homorne receptor status b) across the intrinsic molecular subtypes in whole cohort c) across the intrinsic molecular subtypes in hormone receptor positive tumors d) across the intrinsic molecular subtypes in triple negative tumors. Error bars correspond to standard error of the mean.

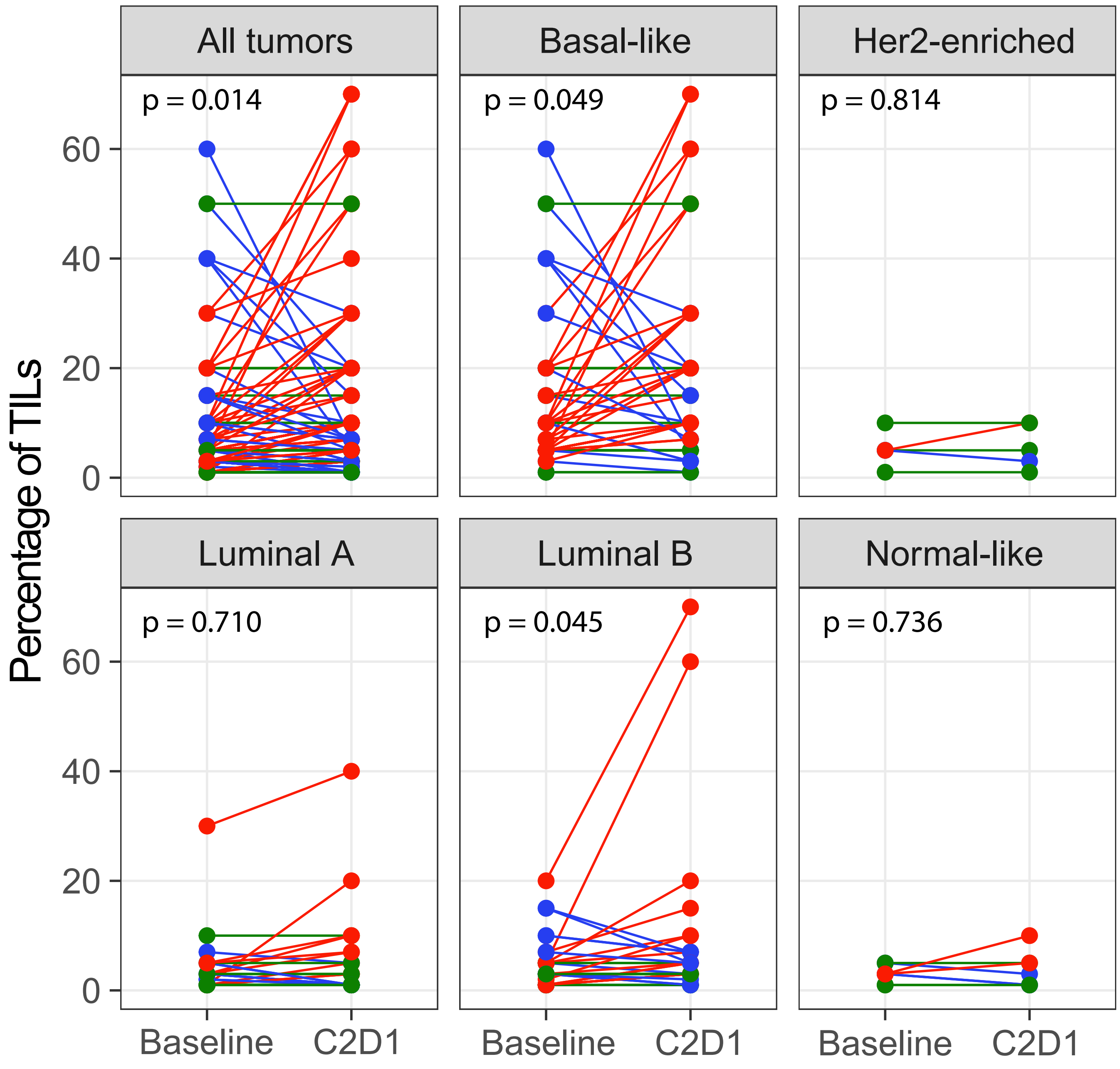

Supplementary Figure 3. Changes in TILs from baseline to cycle 2 day 1 across 134 cases in NEOERIBULIN study: all samples and according to the intrinsic subtype determined in baseline tumor.

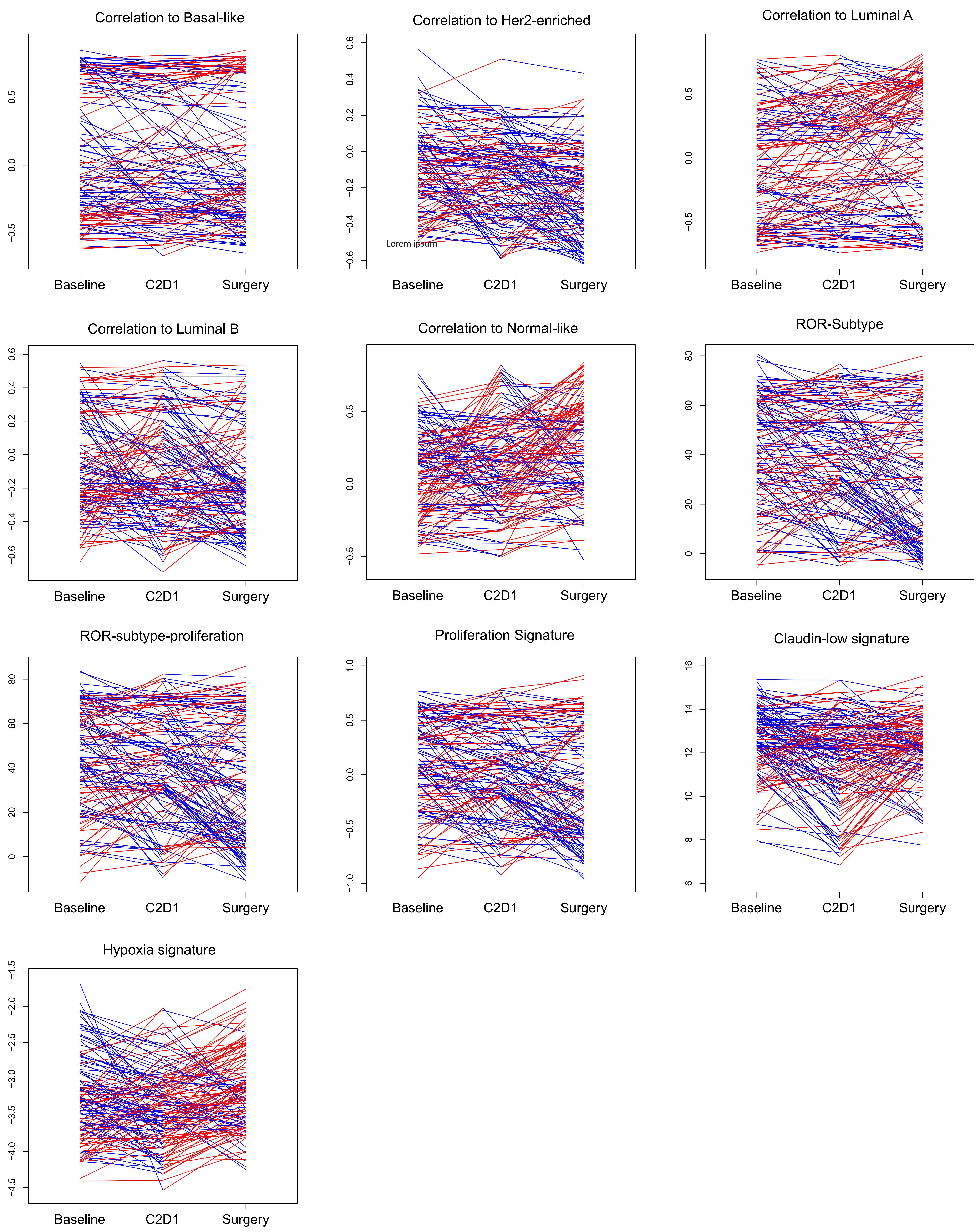

Supplementary Figure 4. Variation of PAM50-based, Claudin low and Hypoxia signature at baseline, Cycle 2 Day1 (C2D1) vs Surgery.

Supplementary Table 1. Gene expression profiles between baseline samples with pathological complete response (pCR) and non pCR. Lists of differentially expressed genes between with pCR and non pCRtumors determined by SAM analysis.

Gene ID	Gene Name	Score(d)	Numerator(r)	Denominator(	Fold Change	q-value(%)
SPAG5	g467	2.50220167	0.84723806	0.33859703	1.79905346	0
E2F1	g154	2.50153527	0.98320885	0.39304217	1.97685745	0
UBE2C	g523	2.26084186	0.90244646	0.3991639	1.86923306	0
MYBL2	g332	2.191624	1.11945349	0.5107872	2.17264654	0
MELK	g310	1.92380796	0.76029703	0.39520422	1.69383932	0
BLM	g46	1.78465928	0.72914451	0.40856231	1.65765585	0
HN1	g228	1.77633775	0.62145105	0.3498496	1.53842173	0
CENPI	g109	1.76813897	0.64376514	0.36409193	1.56240139	0
RAD51C	g419	1.73105614	0.51478059	0.29737949	1.42877683	0
IDH2	g233	1.68406209	0.61645722	0.36605374	1.53310576	0
CDT1	g103	1.64507965	0.67766033	0.41193162	1.59954361	0
RRM2	g438	1.63903368	0.73997318	0.45146917	1.67014479	0
PLA1A	g389	1.61077406	0.70768744	0.43934618	1.63318411	0
RAD51	g417	1.58931631	0.55945243	0.35200824	1.47370977	0
TAP1	g484	1.58683173	0.74671611	0.47057044	1.67796905	0
EPCAM	g162	1.5538389	0.59427067	0.3824532	1.50970918	0
CENPN	g110	1.55264322	0.49688118	0.32002277	1.41115961	0
KIFC1	g265	1.55043773	0.68555741	0.44217023	1.60832327	0
CDH1	g92	1.5339106	0.76891546	0.50127789	1.70398834	34.1
FANCA	g182	1.53379357	0.76376539	0.4979584	1.69791635	34.1
WDR4	g533	1.53223607	0.39245407	0.2561316	1.31262432	34.1
SLC25A19	g454	1.52705217	0.42117182	0.2758071	1.33901472	34.1
ORC6L	g369	1.52454518	0.72523584	0.47570636	1.65317086	34.1
CDCA5	g88	1.49811521	0.74360375	0.49635952	1.67435303	34.1
PNP	g392	1.49548907	0.41696206	0.27881318	1.33511319	34.1
CAND1	g65	1.49133599	0.35576586	0.23855513	1.27966471	34.1
BIRC5	g45	1.47960822	0.68558307	0.46335447	1.60835187	34.1
PRC1	g397	1.47104646	0.58428931	0.39719297	1.49930023	34.1
STAT1	g475	1.46015149	0.64341684	0.44065074	1.56202424	34.1
CDC25C	g84	1.45481645	0.51894616	0.35670902	1.43290817	34.1
CDKN2A	g98	1.43283996	0.69273184	0.4834677	1.61634127	34.1
GINS2	g205	1.43148198	0.60662284	0.4237726	1.52269061	34.1
CDC6	g86	1.40581588	0.52442839	0.37304202	1.43836357	34.1
DLGAP5	g150	1.39156425	0.52088535	0.37431642	1.43483551	34.1
HJURP	g226	1.39016332	0.61551304	0.44276311	1.53210273	34.1
CCNB1	g72	1.36489414	0.50064914	0.36680437	1.41485003	34.1
NUP93	g365	1.35295651	0.37147607	0.27456616	1.29367576	34.1
ITCH	g250	1.34882047	0.29716242	0.22031281	1.22872531	34.1
ASF1A	g29	1.34661001	0.3562872	0.26458083	1.28012722	34.1
UBE2T	g524	1.34586045	0.5027102	0.37352327	1.41687275	34.1
PSMA7	g400	1.3302479	0.3143288	0.2362934	1.24343302	34.1
AURKA	g35	1.32629618	0.46162764	0.34805773	1.37709457	34.1
RANBP1	g422	1.31399788	0.33534729	0.25521144	1.26168109	34.1
HRAS	g229	1.31382685	0.38184061	0.29063237	1.30300318	34.1

KIF4A	g264	1.31351538	0.48939775	0.37258623	1.40385871	34.1
CMC2	g125	1.3113857	0.33442272	0.25501477	1.26087279	34.1
CDC123	g81	1.28998798	0.39078505	0.30293697	1.31110665	34.1
IDO1	g234	1.28853398	0.9755238	0.75708039	1.966355	34.1
BUB1	g55	1.2827965	0.56725799	0.44220419	1.48170474	34.1
MTFR2	g326	1.27957213	0.44061274	0.34434381	1.35718062	34.1
SRC	g473	1.25585927	0.39489691	0.3144436	1.3148488	34.1
RAB35	g413	1.23996843	0.25256314	0.20368514	1.19132178	34.1
CLDN3	g121	1.23929181	0.72999782	0.58904434	1.65863659	34.1
CDC45	g85	1.22610089	0.53990074	0.44033957	1.45387248	34.1
TOP2A	g508	1.21919318	0.5214901	0.42773377	1.43543708	34.1
SUV39H2	g482	1.21180304	0.42900373	0.354021	1.34630355	34.1
LAG3	g285	1.20459999	0.67802536	0.56286349	1.59994838	34.1
FGFR4	g190	1.19538937	0.67822233	0.5673652	1.60016684	34.1
ABCB1	g4	1.19461238	0.51629569	0.43218679	1.4302781	34.1
BCL2A1	g43	1.18268183	0.49746692	0.42062616	1.41173267	34.1
MCM2	g306	1.17856812	0.39388123	0.33420319	1.31392345	34.1
PCNA	g374	1.17607645	0.36043734	0.30647442	1.28381502	34.1
PSMD14	g401	1.17339007	0.28183119	0.24018542	1.21573702	34.1
NR4A3	g358	1.1573374	0.5617807	0.48540789	1.47609002	34.1
CDK4	g95	1.14906935	0.29439633	0.25620414	1.22637172	34.1
CYB5B	g142	1.14325173	0.26360375	0.23057367	1.20047366	34.1
CKS2	g120	1.13893781	0.43779328	0.38438735	1.35453088	34.1
GTPBP4	g220	1.13183318	0.3193146	0.28212161	1.24773763	34.1
NUP88	g364	1.12253536	0.27991236	0.24935727	1.21412112	34.1
KIF23	g262	1.10750215	0.42078391	0.37993959	1.33865474	34.1
PTTG1	g408	1.10631296	0.43153994	0.39007041	1.34867239	34.1
EPSTI1	g164	1.09811454	0.47428007	0.43190401	1.3892248	34.1
TYMS	g522	1.09808283	0.47320733	0.43093956	1.3881922	34.1
KNTC2	g270	1.08809288	0.47672621	0.43813007	1.39158228	34.1
ZNF217	g540	1.08623374	0.4285385	0.39451776	1.34586947	34.1
MCM3	g307	1.07556657	0.3338415	0.31038664	1.26036491	34.1
CHEK1	g113	1.06311019	0.49252653	0.46328832	1.40690657	34.1
EZH2	g173	1.04798569	0.41593337	0.39688841	1.33416156	34.1
PDSS1	g376	1.0418544	0.34129038	0.32757972	1.26688922	34.1
TTK	g517	1.04142546	0.47742499	0.45843415	1.39225647	34.1
SLC9A3R1	g461	1.04087509	0.76395013	0.73394986	1.69813379	34.1
CEP55	g111	1.03295461	0.46819342	0.45325652	1.38337608	34.1
TP53	g510	1.03248319	0.36876464	0.35716285	1.29124668	34.1
IGFBP2	g239	1.02579961	0.62099844	0.60537988	1.53793917	34.1
SPDEF	g469	1.0207189	1.02971217	1.00881073	2.04161689	34.1
HIF1A	g225	1.0175182	0.30838144	0.30307216	1.23831765	34.1
CHEK2	g114	1.01501248	0.29780462	0.29339996	1.22927238	34.1
CENPA	g107	1.01363097	0.44613714	0.44013764	1.36238753	34.1
HSPA14	g230	1.0122777	0.31864484	0.31478007	1.24715851	34.1
ARAF	g27	1.00063042	0.23032767	0.23018256	1.17310136	34.1
CDK1	g94	0.9952025	0.4189761	0.42099583	1.33697835	34.1
NCAPH2	g337	0.99280552	0.26446329	0.26637976	1.2011891	34.1
JUP	g253	0.98948471	0.2887776	0.29184645	1.22160477	34.1
TK1	g497	0.97933173	0.35460751	0.36209131	1.27863768	34.1

TOR1A	g509	0.97472194	0.23005819	0.23602443	1.17288226	34.1
MIS18A	g315	0.97198187	0.31172816	0.32071397	1.2411936	34.1
PHGDH	g381	0.96960422	0.57155284	0.58947025	1.4861223	34.1
FBP1	g186	0.95632215	0.5864487	0.61323342	1.50154603	34.1
CDCA8	g91	0.95508133	0.3804914	0.39838639	1.30178519	34.1
SNRPA1	g465	0.95070372	0.31207712	0.32825907	1.24149386	34.1
CDKN3	g102	0.94734601	0.32343196	0.34140847	1.25130368	34.1
CDC25B	g83	0.94556756	0.31562596	0.33379525	1.24455152	34.1
HSPD1	g231	0.94399073	0.25085582	0.26573971	1.18991277	34.1
CDCA7	g89	0.93559309	0.57692816	0.61664431	1.49166974	34.1
NUDT1	g363	0.93055303	0.22965427	0.24679332	1.17255392	34.1
RHBG	g434	0.923773	0.39430876	0.42684595	1.31431288	34.1
LOC647867	g293	0.92272244	0.46838437	0.50761134	1.3835592	34.1
PIEZO1	g383	0.91938073	0.28306965	0.30789165	1.2167811	34.1
BRCA1	g51	0.91789399	0.38128625	0.41539247	1.3025026	34.1
ATM	g33	0.91523805	0.28574753	0.31221116	1.21904175	34.1
TSPAN13	g516	0.90422881	0.48542851	0.53684257	1.40000164	34.1
C1orf106	g58	0.90282028	0.60326034	0.66819537	1.5191458	34.1
IGF2R	g238	0.90281495	0.21025895	0.23289264	1.15689582	34.1
HMGA1	g227	0.89767059	0.34299208	0.38209126	1.26838444	34.1
CDKN2D	g101	0.87277307	0.2768163	0.3171687	1.21151839	34.1
TMEM208	g503	0.86175431	0.20279489	0.23532797	1.15092585	34.1
SH2B3	g451	0.86102376	0.23797856	0.27639024	1.17933907	34.1
MAD2L1	g299	0.85909628	0.28589641	0.3327874	1.21916756	34.1
PUM1	g410	0.85113138	0.18506207	0.21743067	1.13686588	34.1
CD86	g80	0.84753209	0.28643893	0.33796824	1.2196261	34.1
ADRA2C	g12	0.84599623	0.49802329	0.58868264	1.4122772	34.1
CDCA1	g87	0.84341735	0.40080898	0.47522022	1.32024802	34.1
KIF2C	g263	0.84064991	0.33537342	0.39894541	1.26170394	34.1
GABPB1	g199	0.82823052	0.17855975	0.21559185	1.13175349	39.1
CTSL	g134	0.82741442	0.26663882	0.32225547	1.20300182	39.1
MSH2	g325	0.82387712	0.23674183	0.2873509	1.17832853	39.1
TACC3	g483	0.81899272	0.29961001	0.36582744	1.23081165	39.1
TIMM8A	g496	0.81586179	0.2196929	0.2692771	1.16448568	39.1
CCNE1	g76	0.8097898	0.36132753	0.44619916	1.28460742	39.1
CCND3	g75	0.80956305	0.22297481	0.27542612	1.16713772	39.1
PTGER4	g406	0.80272474	0.26189702	0.32626006	1.19905432	39.1
OCLN	g366	0.78323542	0.34121381	0.43564655	1.26682199	39.1
COX7B	g128	0.7760241	0.16580946	0.21366535	1.12179531	39.1
ACTL8	g8	0.77228519	0.51353904	0.66496036	1.42754778	39.1
ANLN	g21	0.76456611	0.30880824	0.40389999	1.23868405	39.1
NFIB	g345	0.76404717	0.40145535	0.52543268	1.32083967	39.1
IKBKE	g241	0.75427517	0.25238898	0.33461128	1.19117797	39.1
AVL9	g37	0.73045951	0.18637529	0.25514801	1.13790119	43.1
KRT8	g283	0.72995437	0.28540268	0.39098702	1.21875039	43.1
VAMP8	g528	0.72919039	0.20908292	0.28673295	1.15595314	43.1
EPN3	g163	0.72903304	0.30873087	0.42347994	1.23861762	43.1
EVI2A	g171	0.72724108	0.23555015	0.32389556	1.17735562	43.1
CFLAR	g112	0.71286796	0.17985126	0.25229252	1.13276709	43.1
GATA3	g203	0.70875862	0.52564145	0.74163677	1.4395735	43.1

MDM2	g308	0.70697639	0.23565931	0.33333406	1.1774447	43.1
GPR160	g209	0.70099952	0.39689758	0.56618809	1.31667345	43.1
MTOR	g328	0.67918727	0.16043277	0.23621287	1.11762235	45
WDR12	g532	0.66487916	0.16501501	0.24818797	1.12117774	45
POLD1	g393	0.65441399	0.17723391	0.27082843	1.13071388	47.7
FOXM1	g196	0.64640152	0.35037224	0.54203499	1.27488953	47.7
TOMM40	g507	0.6455562	0.16179876	0.25063466	1.11868104	47.7
AARS	g2	0.64134641	0.17978971	0.28033166	1.13271876	47.7
PPP6R1	g395	0.63615637	0.14849996	0.23343311	1.1084164	47.7
GRB7	g212	0.6219269	0.23895109	0.38421088	1.18013433	47.7
DDR1	g148	0.62050182	0.20504569	0.33045139	1.15272285	47.7
ATR	g34	0.62006804	0.15961982	0.25742308	1.11699275	47.7
BTG2	g53	0.6165173	0.2829472	0.45894446	1.21667783	47.7
FABP4	g176	0.60890091	0.48432543	0.79540928	1.39893161	47.7
CEACAM6	g105	0.59371377	0.64664903	1.08915956	1.5655277	47.7
CLDN7	g123	0.59314312	0.27972308	0.47159457	1.21396184	47.7
NOP56	g351	0.59267305	0.14848496	0.25053436	1.10840488	51.3
CDC20	g82	0.58813889	0.28723786	0.4883844	1.22030168	51.3
VAV3	g529	0.57270415	0.34504115	0.60247712	1.27018721	51.3
DDB2	g146	0.57088658	0.1753888	0.30722179	1.12926869	51.3
KDR	g257	0.57072152	0.18569974	0.32537715	1.13736849	51.3
NFKBIA	g347	0.56501888	0.15809032	0.27979653	1.11580918	51.3
EXO1	g172	0.56122012	0.26187115	0.4666104	1.19903282	51.3
KIAA1324	g259	0.55895513	0.39719834	0.71060863	1.31694796	51.3
CAPN6	g67	0.55596903	0.42245413	0.75985192	1.3402054	51.3
PGAM5	g379	0.55329504	0.13250619	0.2394856	1.09619632	51.3
GRHL2	g215	0.5492191	0.19249644	0.35049116	1.1427394	51.3
CDYL	g104	0.53455155	0.12210417	0.22842356	1.08832102	51.3
CKS1B	g119	0.53178088	0.16622908	0.31258943	1.12212165	51.3
CDCA7L	g90	0.53050496	0.18130564	0.3417605	1.13390961	51.3
TOM1L1	g506	0.52424904	0.21513359	0.41036525	1.1608114	51.3
CCNA2	g71	0.5159042	0.18691282	0.36230141	1.13832524	51.3
FAM174B	g179	0.51555616	0.25650639	0.49753336	1.19458242	51.3
CCDC86	g70	0.51500847	0.12842903	0.24937266	1.09310276	51.3
NFKBIE	g349	0.5099447	0.15685076	0.30758386	1.11485089	54.8
S100A9	g443	0.49751612	0.45161918	0.90774783	1.36757427	54.8
MAP2K1	g302	0.49206293	0.10163394	0.20654663	1.072988	54.8
INSIG1	g248	0.49192554	0.16019564	0.32565019	1.11743866	54.8
REEP6	g428	0.4909903	0.41089403	0.83686791	1.32950945	54.8
LRP8	g295	0.4831248	0.21565367	0.44637259	1.16122993	54.8
CCND2	g74	0.47945869	0.16680581	0.34790445	1.12257031	54.8
RFC4	g432	0.46990822	0.16548073	0.35215542	1.12153973	54.8
CABP7	g63	0.46583589	0.25440853	0.54613339	1.19284661	54.8
IGF1	g237	0.43849653	0.22774039	0.51936645	1.17099944	58.6
FABP5	g177	0.43646016	0.18351759	0.42046814	1.13564946	58.6
STMN1	g480	0.43183805	0.13467116	0.31185571	1.09784255	58.6
EMC8	g160	0.42864475	0.13468123	0.31420246	1.09785021	58.6
USP10	g527	0.42617491	0.10027563	0.23529219	1.07197825	58.6
TSHZ1	g515	0.4248853	0.140729	0.33121646	1.10246206	58.6
SPINT1	g470	0.42134081	0.14094743	0.3345212	1.10262899	58.6

COG8	g126	0.42008247	0.0920651	0.21915959	1.06589483	58.6
KRT18	g276	0.41545389	0.22369779	0.53844192	1.16772276	58.6
MKI67	g316	0.41401442	0.21849218	0.52774051	1.16351691	58.6
MYO5C	g334	0.41274859	0.16434324	0.39816791	1.12065581	58.6
PNO1	g391	0.40954969	0.09759354	0.23829475	1.0699872	58.6
P4HTM	g371	0.40326006	0.19827586	0.49168237	1.14732639	58.6
GGH	g204	0.40064911	0.19815287	0.49457958	1.14722858	58.6
NCS1	g338	0.38729645	0.11687546	0.3017726	1.08438379	58.6
NPM2	g356	0.3845344	0.15822803	0.41147952	1.11591569	58.6
CHUK	g117	0.38412229	0.08441383	0.21975771	1.06025687	58.6
FIGF	g191	0.37861306	0.18260528	0.48230053	1.13493154	58.6
CTSV	g135	0.37388788	0.12320382	0.32952077	1.08915088	58.6
IL6R	g244	0.37309673	0.11997579	0.32156753	1.08671663	58.6
LOC642077	g291	0.35549595	0.12958377	0.36451546	1.09397803	58.6
YBX1	g536	0.35054663	0.13904443	0.39665031	1.10117551	58.6
PIR	g387	0.34377711	0.14638346	0.42580921	1.10679149	58.6
PIK3CA	g384	0.34373517	0.08831865	0.25693807	1.06313046	58.6
RELA	g429	0.33888492	0.06837141	0.20175405	1.04853237	58.6
LSR	g297	0.33883305	0.12300861	0.36303605	1.08900352	58.6
SLC5A6	g458	0.33456316	0.12923781	0.38628822	1.09371572	58.6
MLKL	g318	0.32829895	0.11102957	0.33819653	1.07999869	58.6
TIMM17A	g495	0.32161242	0.0897072	0.27892952	1.06415418	58.6
PREP	g398	0.31539054	0.09381124	0.29744469	1.0671857	58.6
KRT19	g277	0.31532736	0.19054027	0.60426179	1.141191	58.6
SNRPD1	g466	0.30905227	0.08172546	0.26443897	1.05828299	58.6
BYSL	g56	0.3066679	0.08871222	0.28927782	1.06342053	58.6
LOC389332	g289	0.30140548	0.11521663	0.38226454	1.08313767	58.6
RAF1	g420	0.2988303	0.06701848	0.22426935	1.04754954	58.6
AKT1	g17	0.29697927	0.0858068	0.28893194	1.06128108	58.6
S100A8	g442	0.29696065	0.25133655	0.84636312	1.19030934	58.6
PDXK	g377	0.2943609	0.09010945	0.30611895	1.06445093	58.6
MPP1	g322	0.28504735	0.09438599	0.33112389	1.06761093	64.6
CLDN4	g122	0.28416171	0.12931527	0.45507634	1.09377446	64.6
ERBB4	g167	0.28208479	0.27807264	0.98577679	1.21257387	64.6
ATAD2	g31	0.28155851	0.10000598	0.35518722	1.07177791	64.6
NACC2	g335	0.27893529	0.07961735	0.28543304	1.05673772	64.6
PSPHL	g403	0.27155465	0.15936877	0.58687551	1.11679839	64.6
IGBP1	g236	0.25724096	0.07362281	0.28620173	1.05235598	64.6
FZD6	g197	0.25178238	0.09639003	0.38283072	1.06909498	64.6
NPEPPS	g355	0.24276654	0.07436811	0.30633591	1.05289977	64.6
MTHFD1L	g327	0.24208616	0.07610739	0.31438144	1.05416989	64.6
BRCA2	g52	0.24100727	0.10205902	0.42346862	1.07330419	64.6
ESRP1	g170	0.23707062	0.07487243	0.31582332	1.0532679	64.6
PCDH8	g373	0.2346768	0.14376371	0.61260299	1.10478353	64.6
NEO1	g342	0.23345755	0.08625672	0.36947495	1.0616121	64.6
BLVRA	g47	0.23179165	0.0819566	0.35357876	1.05845256	64.6
KLHL7	g268	0.2270062	0.06302998	0.27765752	1.04465747	64.6
SLC16A3	g453	0.22573243	0.08708469	0.38578724	1.06222154	64.6
TGFBR2	g491	0.21948732	0.07950773	0.3622429	1.05665743	64.6
NF1	g343	0.21649859	0.05785378	0.26722476	1.0409161	64.6

CDKN2C	g100	0.21394353	0.085293	0.39867065	1.06090318	64.6
MRPS17	g323	0.2098435	0.06089839	0.2902086	1.04311512	64.6
ITGA6	g251	0.20807922	0.08436858	0.40546375	1.06022362	64.6
TFRC	g490	0.20805805	0.06595607	0.31700802	1.04677841	64.6
CDH3	g93	0.20446179	0.13044055	0.63797031	1.09462791	64.6
FGFR1	g188	0.20116407	0.10281022	0.51107648	1.0738632	64.6
UIMC1	g526	0.19757185	0.04787286	0.24230606	1.03373963	64.6
GAL	g200	0.19153373	0.10513083	0.54888937	1.07559193	64.6
RAD17	g415	0.19143558	0.05714581	0.29851197	1.04040541	64.6
MUC5B	g330	0.19087186	0.1800179	0.94313483	1.13289794	64.6
TCF7L1	g486	0.19012654	0.09375056	0.49309557	1.06714081	64.6
MLPH	g319	0.18804133	0.16478815	0.87634007	1.12100145	64.6
ZEB2	g539	0.1808413	0.05906142	0.32659254	1.04178778	64.6
TMCC2	g499	0.17794289	0.09848069	0.55343989	1.07064537	64.6
F11R	g174	0.17547308	0.04930752	0.28099764	1.03476813	64.6
NLN	g350	0.17328511	0.04356024	0.25137901	1.03065411	64.6
CD68	g79	0.17219132	0.05909181	0.34317532	1.04180973	64.6
FLVCR2	g192	0.15751371	0.04919581	0.31232713	1.034688	66
BMI1	g48	0.14219824	0.0655585	0.46103594	1.04648998	66
WIPF2	g534	0.13528459	0.03261916	0.24111514	1.02286742	68.2
CAPN13	g66	0.1287952	0.11042075	0.85733594	1.07954303	68.2
GSTP1	g219	0.12512801	0.05716348	0.45683998	1.04041815	68.2
PARP1	g372	0.1244877	0.03149258	0.25297744	1.02206899	68.2
ATAD3A	g32	0.11932229	0.03014602	0.25264362	1.02111547	68.2
FBXL6	g187	0.1181779	0.04017988	0.33999487	1.02824202	68.2
MKRN2	g317	0.11478012	0.02672676	0.23285185	1.01869824	68.2
SETBP1	g449	0.10958016	0.04503459	0.41097399	1.03170792	68.2
PTDSS1	g404	0.10759365	0.02944627	0.27368035	1.02062032	68.2
GNG11	g207	0.10408398	0.04617412	0.44362374	1.03252315	68.2
TP63	g512	0.09580831	0.05227613	0.54563253	1.03689954	68.2
KLHL9	g269	0.0936645	0.02597578	0.2773279	1.01816811	68.2
PIK3R1	g385	0.08996231	0.03407892	0.37881327	1.02390291	68.2
TP53BP2	g511	0.07608845	0.02726294	0.35830583	1.01907691	68.2
CXCL14	g137	0.0754349	0.05507252	0.73006682	1.03891133	68.2
PTEN	g405	0.0749789	0.01986589	0.26495314	1.01386523	68.2
BCL11A	g41	0.06465507	0.02968087	0.45906493	1.0207863	68.2
CDKN1A	g96	0.06411308	0.02478077	0.38651657	1.01732509	68.2
XBP1	g535	0.06398101	0.03341247	0.52222481	1.02343003	68.2
FAM198B	g180	0.0570153	0.02910657	0.51050456	1.02038003	68.2
KPNA1	g271	0.05527583	0.01168234	0.21134623	1.00813045	68.2
TRIP13	g514	0.05101976	0.01696992	0.33261472	1.01183211	68.2
MAP2K4	g303	0.05046883	0.01369676	0.27139043	1.00953908	68.2
DDIT4	g147	0.04757515	0.01776922	0.3734979	1.01239285	68.2
AR	g26	0.04642605	0.03911706	0.84256699	1.0274848	68.2
ESR1	g169	0.04607853	0.04939105	1.07188849	1.03482804	68.2
SQLE	g472	0.04030139	0.01545994	0.38360815	1.01077364	68.2
RECQL	g427	0.03826288	0.01004703	0.26257889	1.00698837	68.2
SLC7A6	g459	0.0323133	0.00889928	0.27540611	1.00618758	68.2
LHFP	g288	0.02707371	0.00974279	0.35986168	1.00677604	68.2
MIEN1	g314	0.02630704	0.00757352	0.28788953	1.00526337	68.2

FAM214A	g181	0.0241675	0.01245576	0.51539309	1.00867105	68.2
NRAS	g359	0.02175938	0.00606683	0.27881436	1.00421406	68.2
NEK2	g341	0.01848054	0.0099574	0.53880449	1.00692581	68.2
GPR89A	g210	0.00724325	0.00179798	0.24822799	1.00124704	68.2
S100A14	g441	-3.09841055	-1.953828	0.63059042	0.25813041	0
SERPINA3	g448	-2.18132252	-1.65650567	0.75940428	0.31720652	14.4
EIF2S2	g157	-2.13623378	-1.13183023	0.52982508	0.45633644	14.4
ANXA8L2	g23	-1.96685879	-1.69576954	0.86217147	0.30868996	14.4
TWIST1	g520	-1.92112277	-0.78685492	0.40958076	0.57960626	14.4
SCUBE2	g445	-1.91994167	-1.7730591	0.92349634	0.29258767	14.4
FZD7	g198	-1.91695771	-0.73317795	0.38246955	0.60157731	14.4
ACTR3B	g9	-1.81964122	-0.57828482	0.31780156	0.66975957	14.4
STC2	g477	-1.81922775	-1.39473842	0.76666509	0.38031364	14.4
KRT14	g273	-1.81335663	-1.88740594	1.04083549	0.27029263	14.4
SCGB2A2	g444	-1.76706863	-2.5123812	1.42177907	0.17526609	14.4
CITED4	g118	-1.76247048	-1.02382418	0.58090288	0.49181097	14.4
CD44	g78	-1.74593407	-0.59049322	0.33821049	0.66411583	14.4
MUC1	g329	-1.67473444	-1.30457615	0.77897494	0.40484003	19.5
GSTM4	g218	-1.65064571	-0.59310967	0.35931979	0.66291249	19.5
C16orf45	g57	-1.63728268	-0.88807151	0.54240573	0.54033592	19.5
CYR61	g145	-1.62682084	-0.64721945	0.3978431	0.63850975	19.5
CTGF	g131	-1.5562215	-0.6154683	0.39548888	0.65271799	39.1
KRT6A	g280	-1.55589704	-0.75701668	0.48654677	0.59171867	39.1
NUDCD1	g362	-1.54144832	-0.57972879	0.37609356	0.66908955	39.1
KRT23	g278	-1.52224314	-1.35256738	0.8885357	0.39159456	39.1
LAMC2	g287	-1.5184624	-0.96086626	0.63278897	0.51374834	39.1
TMEM158	g502	-1.46331881	-0.79961232	0.54643753	0.57450354	43.1
RAI2	g421	-1.46066667	-0.70071641	0.47972369	0.6152666	43.1
LAMA3	g286	-1.41618617	-0.78604888	0.55504629	0.57993018	43.1
OGN	g368	-1.41366287	-0.96251685	0.68086732	0.5131609	43.1
INHBA	g246	-1.41197386	-0.57067902	0.40417109	0.67329982	43.1
GSTM3	g217	-1.3989936	-0.95310439	0.68127859	0.51651982	45
SEMA3C	g447	-1.38659712	-0.71083617	0.51264795	0.61096593	45
ID4	g232	-1.35788343	-0.72785876	0.53602448	0.6037994	45
VEGFA	g530	-1.35106015	-0.57414019	0.42495531	0.67168644	45
KRT5	g279	-1.34353088	-1.22581121	0.91238038	0.42755704	45
EGFR	g156	-1.33737411	-0.65084267	0.48665715	0.63690819	45
CXCL8	g138	-1.31898641	-0.95185835	0.72165896	0.51696612	47.7
KRT16	g274	-1.30349661	-1.15663309	0.8873311	0.44855814	47.7
PPFIBP1	g394	-1.25548631	-0.37280119	0.29693768	0.77228155	54.8
GREM1	g213	-1.24416908	-0.61533347	0.49457383	0.65277899	54.8
PGR	g380	-1.20462583	-1.1321071	0.93979979	0.45624888	54.8
ABCC3	g5	-1.18723872	-0.53704009	0.45234381	0.68918343	64.6
PTGS2	g407	-1.1690136	-0.60827794	0.52033435	0.65597924	64.6
AHCYL1	g16	-1.16737944	-0.27397209	0.23468984	0.82703937	64.6
DNALI1	g152	-1.16644127	-0.72185489	0.61885233	0.60631739	64.6
MAPT	g305	-1.16445263	-1.00472891	0.86283365	0.49836377	64.6
CYBRD1	g143	-1.11607607	-0.54259191	0.48616033	0.68653639	68.2
CA12	g62	-1.11074778	-0.83801096	0.75445656	0.5594143	68.2
GLRB	g206	-1.09372388	-0.49271421	0.45049232	0.71068679	68.2

AZGP1	g39	-1.07881965	-0.8631559	0.80009286	0.54974866	68.4
AREG	g28	-1.07358053	-0.89086988	0.82981188	0.53928886	68.4
NTN4	g361	-1.05605822	-0.63564429	0.60190269	0.6436533	68.4
RB1	g424	-1.05513409	-0.30566029	0.28968857	0.80907184	68.4
YBX3	g537	-1.02075677	-0.34747496	0.34040917	0.7859585	68.4
IL1B	g242	-1.0105285	-0.39070261	0.38663196	0.76275804	68.4
TMEM125	g500	-1.0068484	-0.4804468	0.47717889	0.71675561	68.4
CXCR1	g139	-1.00364753	-0.32802428	0.32683215	0.79662669	68.4
ERCC1	g168	-0.99206721	-0.28178585	0.28403908	0.82257216	69.4
VIM	g531	-0.98059881	-0.30302656	0.30902196	0.8105502	69.4
CAV1	g68	-0.97582361	-0.34950106	0.35816008	0.78485548	69.4
PDGFRA	g375	-0.97332226	-0.35960999	0.36946652	0.77937524	69.4
PYROXD1	g411	-0.96282351	-0.25515411	0.2650061	0.83789763	69.4
ELSPBP1	g159	-0.95859109	-0.32587859	0.33995579	0.79781238	69.4
ADM	g10	-0.95255548	-0.50219408	0.52720717	0.70603222	69.4
RERG	g431	-0.94562948	-0.62583305	0.66181635	0.64804547	69.4
STRAP	g481	-0.93526958	-0.25620176	0.2739336	0.83728938	69.5
NT5E	g360	-0.92867315	-0.35333638	0.38047442	0.78277176	69.5
AKT3	g18	-0.90676262	-0.36026747	0.39731178	0.77902014	70.2
KRAS	g272	-0.9010121	-0.23332617	0.25896009	0.85067138	70.2
MMP11	g321	-0.8760509	-0.55562513	0.63423841	0.68036219	71
IL6ST	g245	-0.87542526	-0.41570991	0.47486625	0.74965052	71
KRT17	g275	-0.85592856	-0.89191254	1.04204087	0.53889924	71.6
SHC1	g452	-0.84121456	-0.20955512	0.2491102	0.86480387	71.6
PID1	g382	-0.83586447	-0.33853786	0.40501525	0.79084241	71.6
SLC39A6	g455	-0.83014047	-0.49374147	0.59476858	0.71018093	71.6
CRYAB	g130	-0.81739177	-0.62613779	0.7660192	0.6479086	71.6
SLC9A3	g460	-0.81508465	-0.3022219	0.3707859	0.81100241	71.6
OGFRL1	g367	-0.80707378	-0.2768698	0.34305388	0.8253799	71.6
P3H1	g370	-0.79774515	-0.24477564	0.30683438	0.84394703	71.6
INPP4B	g247	-0.79429785	-0.3702976	0.46619489	0.7736229	71.6
KRTAP1.1	g284	-0.78309834	-0.29978512	0.38281925	0.81237339	72
GUSB	g221	-0.77979454	-0.29725355	0.38119471	0.81380015	72
RRP15	g439	-0.77922659	-0.22736135	0.29177822	0.85419577	72
RECK	g426	-0.76341961	-0.28820422	0.37751745	0.81892077	72
KDM4B	g256	-0.75539811	-0.32966577	0.43641329	0.79572081	72
TUBB6	g519	-0.7522859	-0.23025043	0.30606773	0.8524869	72
ZEB1	g538	-0.74612584	-0.26534798	0.35563436	0.83199803	72
PITX1	g388	-0.74457258	-0.49428802	0.66385472	0.70991194	72
ANGPTL4	g20	-0.74029246	-0.40066224	0.54122155	0.75751049	72
UCHL1	g525	-0.73879531	-0.42100255	0.56985005	0.74690541	72
FAP	g184	-0.73526134	-0.32633692	0.443838	0.79755896	72
F3	g175	-0.73498484	-0.33012385	0.44915736	0.7954682	72
RBBP8	g425	-0.73231023	-0.29553812	0.40356956	0.81476837	72
NDRG1	g339	-0.72708583	-0.35528778	0.48864627	0.7817137	72
FBN1	g185	-0.726656	-0.30143668	0.4148272	0.81144394	72
NFIA	g344	-0.72094129	-0.32608654	0.45230666	0.79769739	72
MIA	g313	-0.69824963	-0.62935115	0.90132688	0.6464671	72
MME	g320	-0.69242845	-0.30502164	0.44050998	0.80943008	72.3
FANK1	g183	-0.69149508	-0.35797435	0.5176817	0.78025935	72.3

CLMN	g124	-0.69106066	-0.28119694	0.4069063	0.82290801	72.3
TMEM45B	g505	-0.67831225	-0.49714378	0.73291287	0.70850809	72.3
IRX3	g249	-0.67759943	-0.28578082	0.42175481	0.82029753	72.3
CXCR2	g140	-0.67497271	-0.25002763	0.37042629	0.84088031	72.3
SPINT2	g471	-0.6639891	-0.21385994	0.32208351	0.86222725	72.3
ELOVL5	g158	-0.65246906	-0.30782752	0.47178869	0.80785735	72.8
KRT6C	g282	-0.63836801	-0.45115021	0.70672434	0.73145945	72.8
PLOD1	g390	-0.63667123	-0.18860161	0.29623077	0.87745582	72.8
RGS22	g433	-0.63145088	-0.42952197	0.68021438	0.74250777	72.8
PIP	g386	-0.61908323	-0.87392976	1.41165149	0.5456585	72.8
PSPH	g402	-0.61810789	-0.16131608	0.26098369	0.89420897	72.8
KIAA0040	g258	-0.6129017	-0.28882079	0.47123509	0.81857086	72.8
H19	g222	-0.6109832	-0.35881791	0.58727951	0.77980326	72.8
TUBA4A	g518	-0.6076652	-0.28198659	0.46404926	0.82245772	72.8
GOLT1A	g208	-0.6068491	-0.28927447	0.4766827	0.81831349	72.8
STK38L	g479	-0.59608321	-0.22190619	0.37227385	0.85743179	72.8
KRT6B	g281	-0.58842876	-0.56145758	0.95416407	0.67761721	72.8
CTNNB1	g132	-0.58839057	-0.16578431	0.28175895	0.89144376	72.8
KIT	g266	-0.57582836	-0.28793884	0.50004281	0.81907142	73.2
KLF4	g267	-0.5682346	-0.23871892	0.42010627	0.84749754	73.2
APH1B	g25	-0.55651994	-0.21587347	0.3878989	0.8610247	73.2
NAT1	g336	-0.54097152	-0.43480896	0.80375574	0.73979171	73.2
C8orf33	g61	-0.53836337	-0.1833127	0.34049995	0.88067847	73.2
LRRC2	g296	-0.53340274	-0.17874248	0.33509853	0.88347274	73.2
DSP	g153	-0.53137527	-0.25790244	0.48534897	0.83630295	73.2
MET	g311	-0.52888793	-0.27080426	0.51202579	0.82885735	73.2
SMIM14	g462	-0.52522182	-0.24205258	0.46085782	0.84554147	73.2
AGR2	g14	-0.52514955	-0.60584485	1.15366155	0.65708647	73.2
KCTD1	g255	-0.52139759	-0.17559965	0.33678646	0.88539943	73.2
NFKBIB	g348	-0.52114222	-0.12970237	0.24888095	0.91402	73.2
AGR3	g15	-0.51722953	-0.67390058	1.30290429	0.6268097	73.2
ECE2	g155	-0.51625524	-0.22215087	0.4303121	0.85728638	73.2
TGFBR3	g492	-0.5089568	-0.25777725	0.50648159	0.83637553	73.2
ME1	g309	-0.50780107	-0.20882991	0.41124355	0.86523869	73.2
ST18	g474	-0.50667096	-0.19359569	0.38209352	0.87442364	73.2
TFF1	g488	-0.50616552	-0.6779163	1.33931742	0.62506742	73.2
PEX11G	g378	-0.50580801	-0.20050054	0.39639654	0.87024858	73.2
AXL	g38	-0.50223731	-0.14122057	0.28118295	0.90675169	73.2
A1CF	g1	-0.50182262	-0.20518102	0.4088716	0.86742984	73.2
CBX7	g69	-0.50104028	-0.16083151	0.32099516	0.89450937	73.2
MAGOHB	g301	-0.49323412	-0.15984939	0.3240842	0.89511851	73.2
BRAF	g50	-0.48758672	-0.12652249	0.25948715	0.91603683	73.2
TRIM29	g513	-0.47727686	-0.34168908	0.71591379	0.78911689	73.2
CRIM1	g129	-0.47193932	-0.1577686	0.33429848	0.89641047	73.2
SNAI1	g464	-0.46424146	-0.15170573	0.32678195	0.90018552	73.2
ACOT4	g7	-0.46344117	-0.19382255	0.41822471	0.87428615	73.2
TWIST2	g521	-0.45164076	-0.19995066	0.44272058	0.87058034	73.2
MRPS35	g324	-0.44707264	-0.13818162	0.30908092	0.90866372	73.2
MYC	g333	-0.44518588	-0.1690659	0.37976475	0.88941836	73.2
ABAT	g3	-0.44432421	-0.22856892	0.51441923	0.85348108	73.2

LRIG1	g294	-0.43778985	-0.18056984	0.41245779	0.88235442	73.2
CDKN2B	g99	-0.4305271	-0.17758859	0.41249109	0.88417963	73.2
RAB25	g412	-0.42477295	-0.18067386	0.42534221	0.88229079	73.2
SPATA7	g468	-0.42202314	-0.14129266	0.33479837	0.90670638	73.2
ABCC8	g6	-0.41866144	-0.33273762	0.79476539	0.79402833	73.2
STAT3	g476	-0.39743347	-0.10179103	0.25612093	0.9318754	73.2
NOTCH3	g354	-0.39303507	-0.11956421	0.30420747	0.92046565	73.2
CYCS	g144	-0.37942018	-0.09227508	0.24320024	0.93804232	73.2
GSTM1	g216	-0.35720489	-0.40703273	1.13949372	0.75417293	73.2
NQO1	g357	-0.35239228	-0.18681017	0.53011992	0.87854606	73.2
ANXA1	g22	-0.34872746	-0.12734407	0.36516789	0.91551532	73.2
COX6C	g127	-0.3484673	-0.19195474	0.55085439	0.87541879	73.2
FNBP1	g193	-0.34818497	-0.09482422	0.27233863	0.93638633	73.2
SMO	g463	-0.345638	-0.14203164	0.410926	0.90624206	73.2
RINT1	g435	-0.3437824	-0.08201754	0.238574	0.94473555	73.2
TM7SF3	g498	-0.34086342	-0.1099563	0.3225817	0.92661613	73.2
KIF13B	g260	-0.33982767	-0.14253414	0.41943064	0.90592647	73.2
CAMK2N1	g64	-0.33651464	-0.19207932	0.57079039	0.8753432	73.2
THY1	g494	-0.3345823	-0.1249826	0.37354816	0.9170151	73.2
BOP1	g49	-0.33398095	-0.15832289	0.47404768	0.89606613	73.2
MAP7D3	g304	-0.33385655	-0.1214055	0.36364571	0.91929162	73.2
ERBB2	g165	-0.3313972	-0.14125075	0.42622796	0.90673272	73.2
HGH1	g224	-0.32709947	-0.09439105	0.28856987	0.93666752	73.2
CXCL1	g136	-0.32615229	-0.22058675	0.67633053	0.85821633	73.2
NOTCH1	g352	-0.32488401	-0.10119961	0.31149458	0.93225749	73.2
AFF3	g13	-0.32088268	-0.25821702	0.80470851	0.83612062	73.2
IKBKB	g240	-0.31196785	-0.09709681	0.3112398	0.93491246	73.2
ERBB3	g166	-0.30014728	-0.14675091	0.48892966	0.90328245	73.2
CHPF	g115	-0.29954837	-0.11403466	0.38068863	0.92400038	73.2
BAG1	g40	-0.29850032	-0.10641582	0.35650154	0.9288929	73.2
KIF20A	g261	-0.29127174	-0.09027594	0.30993718	0.93934307	73.2
BCL2	g42	-0.28800023	-0.15468536	0.53710152	0.89832827	73.2
NOTCH2	g353	-0.28083455	-0.08535301	0.30392632	0.94255388	73.2
BDNF	g44	-0.28027694	-0.13737015	0.49012289	0.90917496	73.2
TCEAL1	g485	-0.276325	-0.140237	0.50750745	0.90737009	73.2
TMEM139	g501	-0.27411763	-0.19826293	0.72327683	0.87159938	73.2
SLC52A2	g457	-0.26907143	-0.08302259	0.30855222	0.94407764	73.2
FGFR2	g189	-0.26873886	-0.1632012	0.60728545	0.8930413	73.2
ADRA2A	g11	-0.26554246	-0.132732	0.49985226	0.91210259	73.2
MGC18216	g312	-0.25954192	-0.15303211	0.58962385	0.8993583	73.2
RELB	g430	-0.24388851	-0.08126836	0.3332193	0.94522628	73.2
PROM1	g399	-0.23986863	-0.24102013	1.00480054	0.84614679	73.2
BTG3	g54	-0.23512509	-0.09076933	0.38604696	0.93902188	73.2
THBS1	g493	-0.23512354	-0.08920615	0.37940119	0.94003987	73.2
GARS	g202	-0.22989706	-0.06000471	0.26100686	0.95926099	73.2
RAD51B	g418	-0.22786996	-0.07040237	0.30895855	0.95237234	73.2
LTBP2	g298	-0.22723513	-0.08341651	0.36709338	0.94381989	73.2
SLC40A1	g456	-0.22253145	-0.13488699	0.606148	0.91074117	73.2
MAGEA1	g300	-0.21494758	-0.11468229	0.53353609	0.92358568	73.2
C1orf21	g59	-0.21277019	-0.11673335	0.54863584	0.92227356	73.2

C4orf32	g60	-0.21023249	-0.10925141	0.51966951	0.92706898	73.2
AVEN	g36	-0.20538854	-0.05035127	0.24515133	0.96570117	73.2
DNAJC12	g151	-0.19971989	-0.16437291	0.82301726	0.89231629	73.2
KCNJ15	g254	-0.19755832	-0.06917372	0.35014329	0.95318376	73.2
SFRP1	g450	-0.19017974	-0.17077512	0.89796693	0.88836526	73.2
HEXIM1	g223	-0.19010189	-0.06430441	0.33826288	0.95640633	73.2
RRAGD	g437	-0.18846704	-0.08453986	0.44856574	0.94308528	73.2
DEGS2	g149	-0.18307287	-0.17638296	0.96345766	0.88491883	73.2
LOC647456	g292	-0.15686994	-0.08224188	0.52426797	0.94458866	73.2
GALNT7	g201	-0.15612745	-0.07828288	0.50140371	0.94718433	73.2
PUF60	g409	-0.15441112	-0.04363939	0.28261819	0.97020439	73.2
CCND1	g73	-0.13884786	-0.0785233	0.56553481	0.9470265	73.2
S100A11	g440	-0.13793191	-0.04358363	0.31597927	0.97024189	73.2
TFF3	g489	-0.12913337	-0.16906877	1.309257	0.8894166	73.2
LOC400043	g290	-0.12375857	-0.04394073	0.35505205	0.97000176	73.2
CELSR1	g106	-0.11823983	-0.06445392	0.54511172	0.95630723	73.2
RNF103	g436	-0.11257521	-0.03492155	0.31020637	0.97608484	73.2
EMP3	g161	-0.11220984	-0.03402012	0.30318306	0.97669491	73.2
CHST11	g116	-0.11215871	-0.03237966	0.28869502	0.97780612	73.2
FAM171A1	g178	-0.10920314	-0.05141898	0.47085629	0.96498674	73.2
PRAME	g396	-0.1058045	-0.11159297	1.05470906	0.92556553	73.2
IFT74	g235	-0.10253683	-0.03733111	0.36407513	0.97445596	73.2
NDUFAF4	g340	-0.1005793	-0.02991384	0.29741545	0.97947879	73.2
NFKB1	g346	-0.09541724	-0.03233376	0.33886712	0.97783723	73.2
CXXC5	g141	-0.09268406	-0.04085975	0.44084978	0.97207548	73.2
ASUN	g30	-0.08404406	-0.02451251	0.29166262	0.98315275	73.2
STK11	g478	-0.08171309	-0.02509299	0.30708647	0.98275726	73.2
RAD50	g416	-0.0764943	-0.02169638	0.28363393	0.98507373	73.2
ITGB1	g252	-0.07600104	-0.0204206	0.26868846	0.98594522	73.2
TMEM25	g504	-0.07550462	-0.03523658	0.4666811	0.97587172	73.2
CTPS1	g133	-0.07471921	-0.02380139	0.31854447	0.98363748	73.2
FOXC1	g195	-0.07183613	-0.05176181	0.72055402	0.96475746	73.2
TFAM	g487	-0.07129115	-0.0195339	0.27400178	0.98655138	73.2
IL6	g243	-0.06732282	-0.03013274	0.44758581	0.97933019	73.2
CDKN1B	g97	-0.06668082	-0.02375534	0.35625447	0.98366888	73.2
GPSM2	g211	-0.05731634	-0.02265786	0.39531247	0.98441745	73.2
ALDH1A1	g19	-0.05037217	-0.02263781	0.44941112	0.98443113	73.2
GRHL1	g214	-0.04338213	-0.01846615	0.42566255	0.98728181	73.2
RARA	g423	-0.0432454	-0.01824118	0.42180635	0.98743577	73.2
APC	g24	-0.03208012	-0.00842814	0.26272166	0.99417509	73.2
SEH1L	g446	-0.03094265	-0.00797054	0.25759082	0.99449048	73.2
RACGAP1	g414	-0.02687955	-0.00729823	0.27151608	0.99495403	73.2
MYB	g331	-0.01956932	-0.01388147	0.70934871	0.99042424	73.2
FOXA1	g194	-0.01726146	-0.02217158	1.28445547	0.98474932	73.2
CD24	g77	-0.01380927	-0.00875477	0.63397796	0.99395003	73.2
CENPF	g108	-0.00216195	-0.00097318	0.45013795	0.99932567	73.2

Supplementary Table 2. Gene expression profiles between C2D1 samples with pathological complete response (pCR) and non pCR. Lists of differentially expressed genes between with pCR and non pCR tumors determined by SAM analysis.

GeneID	GeneName	Score(d)	Numerator(r)	Denominator(	FoldChange	q-value(%)
MME	MME	3.64931449	1.77040351	0.48513317	3.41149359	0
CAV1	CAV1	3.44984139	1.51639161	0.43955401	2.8607464	0
FABP4	FABP4	2.86348176	2.69156936	0.93996386	6.46015758	0
LHFP	LHFP	2.71408392	1.01329602	0.37334734	2.01851739	0
FIGF	FIGF	2.64084957	1.53106329	0.57976165	2.88998758	0
GNG11	GNG11	2.61966619	1.30804676	0.49931811	2.47606083	0
TWIST2	TWIST2	2.54432169	1.1016639	0.43298923	2.14602056	0
ADM	ADM	2.53575497	1.3006858	0.51293828	2.46345958	0
ALDH1A1	ALDH1A1	2.50455155	1.26366075	0.50454571	2.40104218	0
KDR	KDR	2.5042954	0.76743821	0.30644875	1.70224442	0
CDKN2C	CDKN2C	2.49709467	0.93586475	0.37478145	1.91303696	0
ABCB1	ABCB1	2.45027392	1.05785369	0.43172875	2.08183206	0
RECK	RECK	2.422566	0.9118808	0.37641113	1.88149675	0
IGF1	IGF1	2.39346633	1.21174238	0.50627091	2.31617198	0
RAI2	RAI2	2.34855683	1.06024794	0.45144658	2.08528987	0
ZEB2	ZEB2	2.34233387	0.8772785	0.37453179	1.83690689	0
MAP7D3	MAP7D3	2.30836051	0.84439027	0.36579653	1.79550575	0
CBX7	CBX7	2.2778503	0.79074059	0.34714335	1.72996229	0
OGN	OGN	2.27069265	1.52685135	0.67241656	2.88156257	0
CXCR1	CXCR1	2.26991874	0.96782812	0.42637126	1.95589391	0
COG8	COG8	2.25663767	0.49627502	0.2199179	1.41056683	0
ZEB1	ZEB1	2.24097556	0.71677265	0.31984849	1.64350136	0
TWIST1	TWIST1	2.14750224	1.00429468	0.46765711	2.00596256	1.94444444
LOC400043	LOC400043	2.09806148	0.67947596	0.32385894	1.6015579	1.94444444
PID1	PID1	2.07243779	0.78292194	0.37777826	1.72061216	1.94444444
ADRA2A	ADRA2A	2.03592102	0.94136036	0.46237568	1.92033813	1.94444444
CAPN6	CAPN6	2.03369162	1.52487372	0.74980577	2.87761527	1.94444444
AXL	AXL	2.01015548	0.54026834	0.26876943	1.45424298	1.94444444
PTEN	PTEN	1.96796735	0.5036299	0.25591375	1.41777628	2.74509804
ANXA1	ANXA1	1.96012551	0.7374221	0.37621167	1.66719412	2.74509804
FABP5	FABP5	1.94535086	0.85181871	0.43787408	1.80477465	2.74509804
CCND2	CCND2	1.93859028	0.72386271	0.37339644	1.65159816	2.74509804
TSHZ1	TSHZ1	1.92868528	0.5645563	0.29271561	1.47893259	2.74509804
CYBRD1	CYBRD1	1.92311646	0.8617007	0.44807515	1.81717921	2.74509804
F3	F3	1.90646999	0.85368532	0.44778325	1.80711125	2.74509804
SLC7A6	SLC7A6	1.90202856	0.52019404	0.27349434	1.43414813	2.74509804
MPP1	MPP1	1.87398105	0.67957978	0.36263962	1.60167316	2.74509804
TGFBR3	TGFBR3	1.86983981	0.94880196	0.5074242	1.93026907	2.74509804
KIF20A	KIF20A	1.85333721	0.60442049	0.32612548	1.52036792	2.74509804
PSPHL	PSPHL	1.84629755	1.06709963	0.57796731	2.09521694	2.74509804
ADRA2C	ADRA2C	1.84539033	0.94496631	0.51206853	1.92514393	2.74509804
FGFR1	FGFR1	1.83398736	0.78678863	0.42900439	1.72522991	2.74509804
A1CF	A1CF	1.80971882	0.7166082	0.39597765	1.64331403	4.11764706
ITCH	ITCH	1.77378294	0.38955325	0.2196172	1.30998768	4.11764706
CFLAR	CFLAR	1.76822719	0.49164333	0.27804308	1.40604555	4.11764706
SH2B3	SH2B3	1.75899846	0.48535682	0.27592794	1.39993207	4.11764706

VIM	VIM	1.74370721	0.53001567	0.3039591	1.44394488	4.11764706
CDC25B	CDC25B	1.73646607	0.5349641	0.30807633	1.4489061	4.11764706
NT5E	NT5E	1.73626465	0.6749314	0.38872611	1.59652085	4.11764706
MAP2K1	MAP2K1	1.73361576	0.35558584	0.20511226	1.27950505	4.11764706
PDGFRA	PDGFRA	1.72979881	0.68044078	0.39336412	1.60262932	4.11764706
H19	H19	1.7074136	0.95171128	0.55739938	1.93416554	4.11764706
LRRC2	LRRC2	1.69904103	0.60264114	0.35469487	1.51849392	4.79452055
PIK3CA	PIK3CA	1.69439991	0.45170321	0.26658595	1.36765393	4.79452055
THY1	THY1	1.68726194	0.59897966	0.35500099	1.51464496	4.79452055
TUBB6	TUBB6	1.68257471	0.51449653	0.30577931	1.42849554	4.79452055
STMN1	STMN1	1.60318588	0.49718848	0.31012529	1.41146023	6.91358025
RRAGD	RRAGD	1.59655458	0.65618826	0.41100271	1.5759134	6.91358025
MLKL	MLKL	1.5880806	0.56207434	0.35393313	1.47639048	6.91358025
ME1	ME1	1.57386062	0.59998632	0.38121947	1.51570219	6.91358025
SPATA7	SPATA7	1.56995229	0.50972581	0.32467599	1.42377958	6.91358025
CD68	CD68	1.53110554	0.63913139	0.41743131	1.55739122	7.77777778
RHBG	RHBG	1.52113806	0.63887852	0.42000035	1.55711826	7.77777778
KIT	KIT	1.51005769	0.79095788	0.52379316	1.73022287	7.77777778
CTSL	CTSL	1.50193438	0.54742944	0.36448293	1.46147935	7.77777778
ANGPTL4	ANGPTL4	1.49443969	0.90580391	0.60611607	1.87358822	7.77777778
PTGER4	PTGER4	1.47118198	0.51854443	0.35246791	1.43250923	7.77777778
EMP3	EMP3	1.46107987	0.46662257	0.31936829	1.38187065	7.77777778
FAP	FAP	1.45813947	0.59131203	0.40552502	1.50661629	7.77777778
TGFBR2	TGFBR2	1.45717745	0.59172999	0.40607957	1.50705283	7.77777778
CRIM1	CRIM1	1.45297282	0.5065629	0.34863894	1.42066155	10.2212389
PUM1	PUM1	1.45096027	0.29986521	0.20666673	1.23102939	10.2212389
AKT3	AKT3	1.44493931	0.51178187	0.35418918	1.42581012	10.2212389
NFIB	NFIB	1.42517637	0.65015697	0.45619404	1.56933894	10.2212389
GAL	GAL	1.4174221	0.80488499	0.56785131	1.74700652	10.2212389
P3H1	P3H1	1.41718855	0.40835583	0.28814503	1.32717244	10.2212389
SLC9A3	SLC9A3	1.39839073	0.44856891	0.32077509	1.36468588	10.2212389
FBN1	FBN1	1.37461615	0.4987218	0.36280805	1.41296115	10.2212389
CXCR2	CXCR2	1.36764884	0.45656616	0.33383289	1.37227171	11.584507
NF1	NF1	1.34486885	0.34928085	0.25971369	1.27392544	11.584507
SNAI1	SNAI1	1.34475746	0.4275404	0.31793124	1.34493868	11.584507
NDUFAF4	NDUFAF4	1.32334502	0.37570697	0.28390704	1.2974752	11.584507
NOTCH3	NOTCH3	1.30248498	0.40893665	0.3139665	1.32770686	14.1830065
CYB5B	CYB5B	1.29971699	0.27047299	0.20810145	1.20620322	14.1830065
MAGOHB	MAGOHB	1.28596013	0.37989384	0.2954165	1.3012461	14.1830065
LOC389332	LOC389332	1.24588273	0.5151771	0.41350369	1.42916957	14.1830065
PIR	PIR	1.23915196	0.47752854	0.38536722	1.3923564	17.2891566
DDB2	DDB2	1.22812293	0.36228655	0.29499209	1.28546164	17.2891566
EGFR	EGFR	1.22566222	0.53463143	0.43619802	1.44857204	17.2891566
APH1B	APH1B	1.22183839	0.4457979	0.36485832	1.36206721	17.2891566
PIK3R1	PIK3R1	1.19939748	0.42656986	0.35565346	1.34403421	17.2891566
PPFIBP1	PPFIBP1	1.19694664	0.32776101	0.27383093	1.25506406	17.2891566
IGBP1	IGBP1	1.18571371	0.3457939	0.29163355	1.27085012	17.2891566
CHUK	CHUK	1.1740426	0.24293628	0.20692288	1.18339875	17.2891566
AARS	AARS	1.17110407	0.28707561	0.24513245	1.22016445	17.2891566
IGF2R	IGF2R	1.16196969	0.26988619	0.23226612	1.20571271	20.5172414

NACC2	NACC2	1.1606264	0.3006644	0.25905356	1.23171152	20.5172414
HIF1A	HIF1A	1.15668515	0.34575996	0.29892315	1.27082023	20.5172414
NEO1	NEO1	1.14863755	0.40166493	0.34968815	1.32103156	20.5172414
CDCA7L	CDCA7L	1.12952522	0.33574085	0.29724069	1.26202532	20.5172414
NFIA	NFIA	1.10241154	0.47169289	0.42787368	1.38673574	20.5172414
FAM171A1	FAM171A1	1.10169966	0.43047589	0.39073797	1.34767805	20.5172414
IL6R	IL6R	1.10036274	0.37628238	0.34196212	1.2979928	20.5172414
ITGB1	ITGB1	1.09447469	0.28007687	0.25590072	1.21425958	23.0681818
KLHL9	KLHL9	1.08996286	0.29623849	0.27178769	1.22793865	23.0681818
BCL2A1	BCL2A1	1.08885345	0.47828773	0.43925813	1.39308929	23.0681818
CCND3	CCND3	1.07534051	0.27733138	0.25790099	1.21195101	23.0681818
NUP93	NUP93	1.07147324	0.27184788	0.25371411	1.20735328	23.0681818
MET	MET	1.0668868	0.48633201	0.45584219	1.40087867	23.0681818
NOTCH2	NOTCH2	1.02901463	0.29871325	0.29029058	1.23004684	26.5517241
SMO	SMO	1.01594181	0.34224718	0.33687675	1.2677297	26.5517241
LTBP2	LTBP2	1.00107586	0.36423389	0.36384245	1.28719791	26.5517241
RELA	RELA	0.98903262	0.19618999	0.19836554	1.14566876	26.5517241
IGFBP2	IGFBP2	0.9850245	0.6012325	0.61037315	1.51701201	26.5517241
CXCL14	CXCL14	0.98019517	0.60618087	0.61842875	1.52222421	26.5517241
CHST11	CHST11	0.97372716	0.29228085	0.3001671	1.22457476	26.5517241
ST18	ST18	0.97297759	0.37096714	0.38126997	1.29321948	26.5517241
UCHL1	UCHL1	0.95964769	0.48704913	0.50752911	1.40157518	26.5517241
AVEN	AVEN	0.95711667	0.20820115	0.21752954	1.15524685	26.5517241
RAD51B	RAD51B	0.95545846	0.29383807	0.30753621	1.22589726	26.5517241
NCAPH2	NCAPH2	0.95083525	0.23548255	0.24765862	1.17730045	26.5517241
SLC40A1	SLC40A1	0.93089649	0.51284818	0.55091859	1.42686434	29.5525292
RECQL	RECQL	0.92974505	0.25291616	0.27202743	1.19161332	29.5525292
BDNF	BDNF	0.92209315	0.41609538	0.45125092	1.33431139	29.5525292
RAD17	RAD17	0.91757028	0.25825016	0.28145001	1.19602717	29.5525292
SETBP1	SETBP1	0.91478881	0.35036508	0.38300105	1.2748832	29.5525292
GUSB	GUSB	0.90180086	0.30885038	0.34248179	1.23872022	29.5525292
CDK4	CDK4	0.90171689	0.20255688	0.22463468	1.15073599	29.5525292
CRYAB	CRYAB	0.89732015	0.6057721	0.67509027	1.52179297	29.5525292
APC	APC	0.89237784	0.2228493	0.24972527	1.16703619	29.5525292
INSIG1	INSIG1	0.88898003	0.28693244	0.3227659	1.22004337	29.5525292
FZD6	FZD6	0.88270084	0.32434258	0.36744338	1.25209374	29.5525292
PPP6R1	PPP6R1	0.88009809	0.20002472	0.22727548	1.14871804	29.5525292
RAB35	RAB35	0.84290356	0.16151761	0.19162052	1.11846307	33.5687732
C16orf45	C16orf45	0.84056352	0.3967216	0.471971	1.31651285	33.5687732
IL6ST	IL6ST	0.83063756	0.37297224	0.44901923	1.29501808	33.5687732
NR4A3	NR4A3	0.82888734	0.39433217	0.47573675	1.31433421	33.5687732
KLF4	KLF4	0.81431301	0.32060916	0.39371736	1.24885776	33.5687732
CD86	CD86	0.79928711	0.32173351	0.40252558	1.24983141	35.9964413
CMC2	CMC2	0.79870744	0.18701082	0.23414183	1.13840256	35.9964413
CDKN1B	CDKN1B	0.79554202	0.25660255	0.32255059	1.19466204	35.9964413
RAD51C	RAD51C	0.78844841	0.21854705	0.27718625	1.16356117	35.9964413
KRTAP1.1	KRTAP1.1	0.78155518	0.27645298	0.35372164	1.21121332	35.9964413
TCF7L1	TCF7L1	0.76900289	0.3114239	0.40497104	1.24093186	35.9964413
NFKBIA	NFKBIA	0.75490664	0.20139214	0.26677754	1.14980734	35.9964413
FAM198B	FAM198B	0.73673566	0.31383764	0.42598405	1.24300978	38.4879725

RAD50	RAD50	0.73193238	0.1955203	0.26712891	1.14513707	38.4879725
LOC642077	LOC642077	0.72884914	0.23936539	0.32841554	1.18047328	38.4879725
ASF1A	ASF1A	0.72769983	0.17430276	0.23952563	1.12841892	38.4879725
SHC1	SHC1	0.72323167	0.17768345	0.24567986	1.13106627	38.4879725
EVI2A	EVI2A	0.72200507	0.27071392	0.37494739	1.20640467	38.4879725
PLOD1	PLOD1	0.70726492	0.19618908	0.27739122	1.14566804	38.4879725
GABPB1	GABPB1	0.70127174	0.14456704	0.20614982	1.10539887	38.4879725
TP63	TP63	0.69663286	0.42321885	0.60752065	1.34091598	38.4879725
NCS1	NCS1	0.68254477	0.1949132	0.28556838	1.14465529	40.9868421
MTHFD1L	MTHFD1L	0.67448519	0.19277143	0.28580528	1.14295724	40.9868421
CAMK2N1	CAMK2N1	0.67225014	0.34636548	0.51523303	1.27135372	40.9868421
PTGS2	PTGS2	0.67148418	0.35642077	0.53079549	1.28024575	40.9868421
CDKN2D	CDKN2D	0.66958883	0.19674143	0.29382424	1.14610675	40.9868421
SLC25A19	SLC25A19	0.66950223	0.17347075	0.25910407	1.12776834	40.9868421
CXCL1	CXCL1	0.66916445	0.42750408	0.63886251	1.34490482	40.9868421
KLHL7	KLHL7	0.66820314	0.153605	0.2298777	1.11234553	40.9868421
NUP88	NUP88	0.65125482	0.15134043	0.23238282	1.11060087	40.9868421
CYR61	CYR61	0.61295284	0.2491522	0.40647857	1.18850848	40.9868421
P4HTM	P4HTM	0.60471937	0.27635744	0.45700114	1.21113311	46.9648562
EPSTI1	EPSTI1	0.60153761	0.27600469	0.45883197	1.21083702	46.9648562
DDIT4	DDIT4	0.58912736	0.20278468	0.34421196	1.15091771	46.9648562
ARAF	ARAF	0.58502049	0.12614879	0.2156314	1.09137643	46.9648562
MTOR	MTOR	0.58458381	0.12498872	0.21380805	1.09049921	46.9648562
FBP1	FBP1	0.56831536	0.32996613	0.58060394	1.25698387	46.9648562
GSTP1	GSTP1	0.56200635	0.22445123	0.39937491	1.16833276	46.9648562
GLRB	GLRB	0.55035563	0.24634142	0.44760406	1.18619518	46.9648562
SEMA3C	SEMA3C	0.54592605	0.25645016	0.46975256	1.19453586	46.9648562
C1orf21	C1orf21	0.5411095	0.29300674	0.5414925	1.22519105	49.7147147
TOR1A	TOR1A	0.53752152	0.12405979	0.23079967	1.08979728	49.7147147
FOXC1	FOXC1	0.53544899	0.330122	0.61653305	1.25711968	49.7147147
FAM214A	FAM214A	0.52688167	0.24076963	0.45697098	1.18162285	49.7147147
ITGA6	ITGA6	0.5169663	0.19124026	0.3699279	1.14174483	49.7147147
NPEPPS	NPEPPS	0.51656689	0.15150027	0.29328296	1.11072392	49.7147147
CLMN	CLMN	0.50450816	0.19486115	0.38623984	1.14461399	49.7147147
TIMM8A	TIMM8A	0.50259481	0.12055503	0.23986525	1.08715303	49.7147147
CTNNB1	CTNNB1	0.49813502	0.12502099	0.25097812	1.0905236	49.7147147
KIF13B	KIF13B	0.49262713	0.18775581	0.38113168	1.13899057	49.7147147
CYCS	CYCS	0.48266716	0.11561421	0.23953196	1.08343621	49.7147147
PDXK	PDXK	0.47540637	0.12683913	0.2668015	1.09189879	52.6988636
ERCC1	ERCC1	0.47029386	0.12826821	0.27274056	1.09298092	52.6988636
CDC123	CDC123	0.46246994	0.12502815	0.27034871	1.09052901	52.6988636
HEXIM1	HEXIM1	0.45343299	0.14751519	0.32532964	1.10766006	52.6988636
SNRPA1	SNRPA1	0.45097371	0.1408234	0.31226522	1.1025342	52.6988636
BTG2	BTG2	0.44935606	0.19102219	0.42510207	1.14157227	52.6988636
GREM1	GREM1	0.44851726	0.21580033	0.48114165	1.16134799	52.6988636
DNALI1	DNALI1	0.44753802	0.26530554	0.59281117	1.20189056	52.6988636
CA12	CA12	0.44072086	0.32754797	0.74320959	1.25487875	52.6988636
ID4	ID4	0.44013492	0.22186651	0.50408751	1.16624146	52.6988636
KCNJ15	KCNJ15	0.42858709	0.16908967	0.39452814	1.12434881	52.6988636
MDM2	MDM2	0.42702176	0.14341457	0.33584837	1.10451619	52.6988636

BLVRA	BLVRA	0.40791833	0.12842226	0.31482347	1.09309763	52.6988636
ABCC8	ABCC8	0.40201778	0.3141975	0.78155125	1.24331986	52.6988636
WIPF2	WIPF2	0.40062927	0.09434351	0.23548832	1.0675795	52.6988636
BRAF	BRAF	0.39953609	0.10411588	0.26059193	1.0748355	52.6988636
BMI1	BMI1	0.39607665	0.16619913	0.41961355	1.12209835	52.6988636
NDRG1	NDRG1	0.39479433	0.16165262	0.40946034	1.11856774	52.6988636
FNBP1	FNBP1	0.39345911	0.1051571	0.26726309	1.07561151	52.6988636
BCL11A	BCL11A	0.38955824	0.15371536	0.39458891	1.11243062	52.6988636
TMEM208	TMEM208	0.37946724	0.07806968	0.20573497	1.0556047	55.5050505
CHPF	CHPF	0.37893856	0.14101656	0.37213568	1.10268182	55.5050505
NOP56	NOP56	0.37565603	0.08351409	0.22231531	1.05959584	55.5050505
RINT1	RINT1	0.37234877	0.07728695	0.20756601	1.05503214	55.5050505
PIEZO1	PIEZO1	0.36494467	0.10369238	0.28413178	1.07452003	55.5050505
TRIP13	TRIP13	0.36026924	0.11528047	0.31998422	1.08318561	55.5050505
CDKN2A	CDKN2A	0.36010069	0.15312714	0.42523423	1.11197715	55.5050505
YBX3	YBX3	0.3578039	0.11160169	0.31190741	1.08042707	55.5050505
BCL2	BCL2	0.35517972	0.17591349	0.49528023	1.12967947	55.5050505
ATM	ATM	0.35040105	0.11324246	0.32317957	1.08165653	55.5050505
ABCC3	ABCC3	0.34813263	0.13508451	0.38802598	1.09815714	55.5050505
LAMA3	LAMA3	0.34649143	0.19162687	0.55304937	1.14205083	55.5050505
GSTM4	GSTM4	0.34590188	0.11648651	0.33676171	1.08409149	55.5050505
AFF3	AFF3	0.34236898	0.26594151	0.7767687	1.2024205	55.5050505
CDCA7	CDCA7	0.33783176	0.182158	0.53919737	1.13457973	55.5050505
C4orf32	C4orf32	0.3322149	0.15934131	0.47963325	1.11677714	55.5050505
LAG3	LAG3	0.32461411	0.18544908	0.57129087	1.13717089	55.5050505
RB1	RB1	0.30753026	0.07920429	0.25754959	1.05643521	55.5050505
POLD1	POLD1	0.30614359	0.07783501	0.25424349	1.05543301	55.5050505
ACOT4	ACOT4	0.29994218	0.12464888	0.41557636	1.09024236	55.5050505
IKBKE	IKBKE	0.29494355	0.10201455	0.34587823	1.07327111	56.5517241
RGS22	RGS22	0.29227239	0.21676142	0.7416418	1.16212191	56.5517241
ATR	ATR	0.28890764	0.07165996	0.2480376	1.05092518	56.5517241
SFRP1	SFRP1	0.28743343	0.22652705	0.78810266	1.17001502	56.5517241
CAND1	CAND1	0.27167283	0.06079389	0.2237761	1.04303957	56.5517241
GTPBP4	GTPBP4	0.27056299	0.06786882	0.25084295	1.04816716	56.5517241
PREP	PREP	0.26910234	0.07238528	0.26898791	1.05145367	56.5517241
KRT6A	KRT6A	0.25974499	0.12486976	0.48073983	1.09040929	59.0069284
FLVCR2	FLVCR2	0.2397551	0.07884252	0.32884608	1.05617033	60.7936508
OGFRL1	OGFRL1	0.23441612	0.0750079	0.31997755	1.0533668	60.7936508
PEX11G	PEX11G	0.21731691	0.07652582	0.35213927	1.05447568	60.7936508
SMIM14	SMIM14	0.21005686	0.08963785	0.42673137	1.06410304	60.7936508
NOTCH1	NOTCH1	0.20253389	0.06301312	0.31112385	1.04464527	60.7936508
CDYL	CDYL	0.19993424	0.04182597	0.20919862	1.0294159	60.7936508
NPM2	NPM2	0.19802141	0.0717303	0.36223505	1.05097642	60.7936508
STAT1	STAT1	0.19312723	0.08312979	0.43044052	1.05931363	60.7936508
KPNA1	KPNA1	0.18811457	0.03721654	0.19783978	1.02613215	60.7936508
STK11	STK11	0.18771539	0.05603408	0.29850551	1.03960399	60.7936508
NRAS	NRAS	0.17491902	0.04422815	0.25284928	1.03113137	62.6865672
YBX1	YBX1	0.17344797	0.05869045	0.33837494	1.04151993	62.6865672
AVL9	AVL9	0.17339115	0.0428269	0.24699587	1.03013035	62.6865672
CDKN1A	CDKN1A	0.17151782	0.06681904	0.38957489	1.04740474	62.6865672

EMC8	EMC8	0.16667784	0.04784611	0.2870574	1.03372046	62.6865672
GOLT1A	GOLT1A	0.16339965	0.07831394	0.47927851	1.05578344	62.6865672
RANBP1	RANBP1	0.15835404	0.03912182	0.24705286	1.0274882	62.6865672
ABAT	ABAT	0.15578198	0.07316597	0.469669	1.0520228	62.6865672
CDKN2B	CDKN2B	0.15547635	0.06122568	0.39379415	1.04335179	62.6865672
KDM4B	KDM4B	0.15505818	0.06434187	0.41495306	1.04560784	62.6865672
MKRN2	MKRN2	0.15146774	0.03375614	0.22286028	1.02367386	62.6865672
PRAME	PRAME	0.14473886	0.13521441	0.93419563	1.09825602	62.6865672
NFKB1	NFKB1	0.14349304	0.04538342	0.31627609	1.0319574	62.6865672
RRP15	RRP15	0.14265957	0.04253611	0.29816515	1.02992274	62.6865672
THBS1	THBS1	0.13195232	0.05209849	0.39482816	1.03677188	62.6865672
UIMC1	UIMC1	0.13047338	0.03108553	0.23825193	1.02178066	62.6865672
AKT1	AKT1	0.1283374	0.03349269	0.26097373	1.02348694	62.6865672
NUDT1	NUDT1	0.11948942	0.0277983	0.23264234	1.01945514	62.6865672
PNP	PNP	0.10947637	0.03104756	0.28360057	1.02175377	62.6865672
TFF3	TFF3	0.10464785	0.12957669	1.23821645	1.09397266	64.1422594
GSTM1	GSTM1	0.10140189	0.11600966	1.14405813	1.08373322	64.1422594
PDSS1	PDSS1	0.09935415	0.02744041	0.27618786	1.01920228	64.1422594
IKBKB	IKBKB	0.09473445	0.02799652	0.29552633	1.01959523	64.1422594
NTN4	NTN4	0.09211524	0.05663383	0.61481498	1.04003626	64.1422594
AR	AR	0.07568824	0.05678828	0.75029194	1.04014761	65.1975052
STAT3	STAT3	0.06713948	0.01541642	0.22961787	1.01074315	65.1975052
RNF103	RNF103	0.05842769	0.01665498	0.28505291	1.01161125	66.2106299
WDR12	WDR12	0.05226677	0.01175665	0.22493535	1.00818238	66.2106299
IDO1	IDO1	0.05105528	0.03384585	0.66292556	1.02373751	66.2106299
TAP1	TAP1	0.04853787	0.02076289	0.42776689	1.0144958	66.2106299
MAGEA1	MAGEA1	0.04113197	0.02227103	0.54145291	1.01555687	66.2106299
USP10	USP10	0.03817984	0.00777142	0.2035477	1.00540127	66.2106299
ELOVL5	ELOVL5	0.03291796	0.01441704	0.43796891	1.01004323	66.2106299
CABP7	CABP7	0.03173689	0.01627228	0.51272454	1.01134293	66.2106299
CCDC86	CCDC86	0.0251575	0.00619587	0.24628336	1.00430389	66.2106299
CTGF	CTGF	0.01275956	0.00575863	0.45131876	1.00399956	66.2106299
TMCC2	TMCC2	0.00877664	0.00435388	0.49607635	1.00302244	66.2106299
S100A14	S100A14	-3.40075838	-2.2957553	0.67507157	0.20366143	0
PITX1	PITX1	-2.9548305	-1.96077876	0.66358417	0.25688975	0
ESRP1	ESRP1	-2.92916214	-1.52453641	0.52046843	0.34759123	0
GRHL1	GRHL1	-2.92727111	-1.33567043	0.45628518	0.39620791	0
EPCAM	EPCAM	-2.78755236	-1.38524733	0.49694038	0.38282386	0
CLDN4	CLDN4	-2.76737824	-1.42075991	0.51339564	0.37351552	0
RAB25	RAB25	-2.7300902	-1.6604077	0.60818785	0.31634974	0
SPINT2	SPINT2	-2.59297692	-1.0576775	0.40790086	0.48040481	0
GRHL2	GRHL2	-2.43873978	-1.21886387	0.49979251	0.42962091	2.1875
CENPF	CENPF	-2.36133301	-1.17363032	0.49702025	0.44330443	2.1875
KRT8	KRT8	-2.33186641	-1.06313077	0.4559141	0.47859235	2.1875
LOC647867	LOC647867	-2.24890557	-1.32040628	0.58713282	0.40042216	2.74509804
TMEM125	TMEM125	-2.21383688	-1.09811548	0.49602366	0.46712628	2.74509804
SPINT1	SPINT1	-2.19495139	-0.92542111	0.42161349	0.52652681	2.74509804
CITED4	CITED4	-2.17209967	-1.2167377	0.5601666	0.43025454	2.74509804
UBE2T	UBE2T	-2.10267505	-0.90381412	0.42984013	0.53447185	4.11764706
KRT19	KRT19	-2.09882886	-1.39076416	0.66263819	0.38136275	4.11764706

KRT23	KRT23	-1.96316673	-1.60556509	0.81784449	0.32860695	6.91358025
MUC1	MUC1	-1.95920201	-1.580485	0.80669833	0.33436946	6.91358025
KCTD1	KCTD1	-1.95845769	-0.68018032	0.34730407	0.62408727	6.91358025
CCNB1	CCNB1	-1.91198611	-0.78235105	0.4091824	0.58141853	6.91358025
FOXM1	FOXM1	-1.89780602	-1.06988713	0.56374946	0.47635627	7.77777778
IRX3	IRX3	-1.89278335	-0.86502897	0.45701426	0.54903538	7.77777778
OCLN	OCLN	-1.86607734	-0.97140761	0.52056128	0.51000822	7.77777778
DLGAP5	DLGAP5	-1.8603213	-0.73892011	0.39720026	0.59918769	7.77777778
KRT18	KRT18	-1.85938683	-1.08081863	0.58127691	0.47276049	7.77777778
PGAM5	PGAM5	-1.77580724	-0.4300507	0.24217195	0.7422357	7.77777778
F11R	F11R	-1.77409672	-0.54985288	0.30993399	0.68308978	7.77777778
GRB7	GRB7	-1.76872616	-0.81893597	0.46300891	0.56685986	10.2212389
KIF2C	KIF2C	-1.7467154	-0.71911197	0.41169384	0.60747125	10.2212389
ERBB3	ERBB3	-1.73814715	-1.00565804	0.57858049	0.49804292	10.2212389
CKS1B	CKS1B	-1.73408789	-0.51297348	0.29581746	0.70077661	10.2212389
RRM2	RRM2	-1.71332751	-0.82516372	0.4816147	0.56441815	10.2212389
DSP	DSP	-1.70688271	-0.97426068	0.57078361	0.50900062	10.2212389
ANLN	ANLN	-1.69581385	-0.72575589	0.42796908	0.60468014	10.2212389
TOP2A	TOP2A	-1.6800396	-0.79698002	0.47438169	0.57555272	10.2212389
INHBA	INHBA	-1.65436733	-0.80524177	0.48673699	0.57226617	10.2212389
LAMC2	LAMC2	-1.62754903	-0.96236561	0.59129746	0.5132147	10.2212389
SQLE	SQLE	-1.62546386	-0.61416824	0.37784183	0.65330644	10.2212389
FBXL6	FBXL6	-1.60739186	-0.50621545	0.31492971	0.70406696	10.2212389
DDR1	DDR1	-1.59929124	-0.65809026	0.41148869	0.63371661	10.2212389
KIFC1	KIFC1	-1.59813921	-0.7672452	0.48008659	0.5875383	10.2212389
CLDN7	CLDN7	-1.59396475	-0.81331765	0.51024821	0.5690717	10.2212389
LSR	LSR	-1.58846616	-0.62315271	0.39229839	0.64925058	10.2212389
BIRC5	BIRC5	-1.58371888	-0.82187507	0.51895262	0.56570622	10.2212389
KIF23	KIF23	-1.56465207	-0.68584833	0.4383392	0.62164018	11.584507
EIF2S2	EIF2S2	-1.55888184	-0.79581636	0.51050461	0.57601714	11.584507
NLN	NLN	-1.54185577	-0.36070604	0.23394279	0.77878336	11.584507
SLC39A6	SLC39A6	-1.53690397	-0.92361574	0.60095865	0.52718611	11.584507
HMGA1	HMGA1	-1.53122171	-0.5141261	0.33576202	0.70021696	11.584507
EZH2	EZH2	-1.52016656	-0.57674267	0.3793944	0.67047588	11.584507
PARP1	PARP1	-1.5009939	-0.39231256	0.26136852	0.76190733	11.584507
TYMS	TYMS	-1.49450361	-0.64912782	0.43434342	0.6376657	11.584507
FANK1	FANK1	-1.48836615	-0.77707963	0.52210246	0.58354684	11.584507
MKI67	MKI67	-1.48828305	-0.77401166	0.5200702	0.58478911	11.584507
RFC4	RFC4	-1.47560724	-0.49728092	0.33700087	0.70844074	11.584507
SLC5A6	SLC5A6	-1.47543848	-0.5457128	0.36986483	0.68505285	11.584507
CLDN3	CLDN3	-1.46269479	-0.87420927	0.59767033	0.5455528	11.584507
CDK1	CDK1	-1.46090052	-0.61273064	0.41941982	0.65395776	11.584507
TTK	TTK	-1.45752802	-0.65467591	0.44916866	0.63521817	11.584507
LOC647456	LOC647456	-1.450321	-0.78464713	0.54101618	0.58049392	11.584507
KIF4A	KIF4A	-1.43001332	-0.58478226	0.40893483	0.66674996	11.584507
HGH1	HGH1	-1.42495008	-0.40641747	0.28521524	0.75449463	11.584507
KIAA1324	KIAA1324	-1.40612342	-0.957755	0.68113153	0.51485747	14.1830065
BOP1	BOP1	-1.39338652	-0.6116438	0.43896205	0.6544506	14.1830065
CEP55	CEP55	-1.39153952	-0.64322974	0.46224325	0.64027796	14.1830065
TK1	TK1	-1.38940839	-0.54073637	0.38918462	0.68741995	14.1830065

CD24	CD24	-1.37252355	-0.91750189	0.66847807	0.52942496	14.1830065
HSPD1	HSPD1	-1.34705243	-0.37521135	0.2785425	0.77099246	14.1830065
VEGFA	VEGFA	-1.3154018	-0.51182079	0.38909844	0.70133674	17.2891566
TOM1L1	TOM1L1	-1.31222178	-0.59650622	0.45457729	0.66135362	17.2891566
PUF60	PUF60	-1.31172639	-0.36085174	0.27509681	0.77870471	17.2891566
CDC20	CDC20	-1.31055826	-0.63592444	0.48523172	0.64352833	17.2891566
GINS2	GINS2	-1.28937501	-0.57510201	0.44603161	0.67123879	17.2891566
SERPINA3	SERPINA3	-1.27422952	-0.95828192	0.75204812	0.51466946	17.2891566
TIMM17A	TIMM17A	-1.27314573	-0.36465082	0.28641719	0.77665683	17.2891566
CDH1	CDH1	-1.2637327	-0.72911173	0.57695091	0.60327524	17.2891566
CCNA2	CCNA2	-1.2596798	-0.45067137	0.35776661	0.73170227	17.2891566
VAV3	VAV3	-1.25388322	-0.73537871	0.58648102	0.60066033	17.2891566
CENPA	CENPA	-1.24670262	-0.54487436	0.43705239	0.68545109	17.2891566
PRC1	PRC1	-1.21482048	-0.51323721	0.42247988	0.70064851	17.2891566
AREG	AREG	-1.20803241	-0.9997735	0.82760487	0.50007851	17.2891566
CDCA8	CDCA8	-1.2073401	-0.46728719	0.38703857	0.72332344	17.2891566
PTTG1	PTTG1	-1.19941122	-0.4588083	0.38252794	0.72758702	17.2891566
TOMM40	TOMM40	-1.19837495	-0.30889094	0.25775817	0.8072621	17.2891566
TACC3	TACC3	-1.19664576	-0.43369333	0.36242416	0.74036401	20.5172414
TFRC	TFRC	-1.1952359	-0.38335191	0.32073326	0.7666543	20.5172414
CDC45	CDC45	-1.19138412	-0.54632947	0.45856703	0.68476009	20.5172414
ERBB2	ERBB2	-1.1831809	-0.56348492	0.47624579	0.67666566	20.5172414
STRAP	STRAP	-1.18211548	-0.2813592	0.23801329	0.82281546	20.5172414
CDC6	CDC6	-1.17785235	-0.46623925	0.39583845	0.72384904	20.5172414
HJURP	HJURP	-1.17770094	-0.54538152	0.46309	0.68521018	20.5172414
CDT1	CDT1	-1.17468605	-0.47988735	0.40852392	0.71703361	20.5172414
MSH2	MSH2	-1.16741666	-0.31047295	0.26594871	0.80637737	20.5172414
MYBL2	MYBL2	-1.15957454	-0.58863841	0.50763309	0.6649702	20.5172414
CKS2	CKS2	-1.15521703	-0.46999993	0.4068499	0.72196463	20.5172414
VAMP8	VAMP8	-1.15330579	-0.32186328	0.27907887	0.80003594	20.5172414
EPN3	EPN3	-1.14224815	-0.56924826	0.49835779	0.67396788	20.5172414
CDKN3	CDKN3	-1.13381391	-0.40804217	0.3598846	0.75364543	20.5172414
NEK2	NEK2	-1.1228066	-0.57793151	0.51472044	0.6699236	20.5172414
GGH	GGH	-1.12150965	-0.51998065	0.46364349	0.69738119	20.5172414
CDCA1	CDCA1	-1.11241341	-0.54336975	0.48846027	0.68616634	20.5172414
MRPS35	MRPS35	-1.10476487	-0.32343916	0.29276742	0.79916253	20.5172414
MAD2L1	MAD2L1	-1.09377554	-0.38557087	0.35251371	0.76547604	20.5172414
SCUBE2	SCUBE2	-1.07442704	-0.9137821	0.85048316	0.53079177	20.5172414
CDC25C	CDC25C	-1.0698879	-0.37757475	0.35291057	0.76973047	20.5172414
EXO1	EXO1	-1.0633318	-0.49917226	0.46944168	0.7075126	23.0681818
IL1B	IL1B	-1.05837488	-0.40164589	0.37949303	0.75699418	23.0681818
TP53BP2	TP53BP2	-1.04719156	-0.34825166	0.33255774	0.78553548	23.0681818
MYB	MYB	-1.03811084	-0.73050308	0.70368505	0.60269371	23.0681818
C1orf106	C1orf106	-1.03077654	-0.62726607	0.60853739	0.64740209	23.0681818
TUBA4A	TUBA4A	-1.01597174	-0.43886662	0.43196735	0.73771393	23.0681818
RBBP8	RBBP8	-1.00916436	-0.39108702	0.38753551	0.76255483	23.0681818
RAD51	RAD51	-1.00798355	-0.3663269	0.36342547	0.77575506	23.0681818
ATAD2	ATAD2	-0.99551233	-0.35372155	0.35531609	0.78256281	23.0681818
ECE2	ECE2	-0.99510991	-0.39663816	0.39858729	0.75962634	26.5517241
SLC52A2	SLC52A2	-0.98852236	-0.30250834	0.30602073	0.8108414	26.5517241

CTPS1	CTPS1	-0.98147216	-0.29006091	0.29553657	0.81786753	26.5517241
CDCA5	CDCA5	-0.96868097	-0.48457812	0.50024532	0.71470604	26.5517241
ATAD3A	ATAD3A	-0.96016889	-0.2215858	0.23077794	0.85762223	26.5517241
MCM2	MCM2	-0.94891761	-0.31550887	0.33249343	0.8035675	26.5517241
ACTR3B	ACTR3B	-0.94273591	-0.26700579	0.28322438	0.83104253	26.5517241
S100A11	S100A11	-0.93899452	-0.28031865	0.29853066	0.82340913	29.5525292
MELK	MELK	-0.92479385	-0.34186231	0.36966326	0.78902214	29.5525292
RELB	RELB	-0.90056855	-0.29017805	0.32221651	0.81780112	29.5525292
TFF1	TFF1	-0.88695763	-1.12040447	1.26319955	0.45996485	29.5525292
MUC5B	MUC5B	-0.88303074	-0.75153912	0.85109055	0.59396955	33.5687732
E2F1	E2F1	-0.88288989	-0.37252034	0.42193296	0.7724319	33.5687732
SRC	SRC	-0.87775977	-0.26058547	0.29687562	0.83474909	33.5687732
BUB1	BUB1	-0.86209614	-0.38277544	0.44400552	0.7669607	33.5687732
AZGP1	AZGP1	-0.85256256	-0.7353858	0.86255934	0.60065738	33.5687732
SEH1L	SEH1L	-0.84314358	-0.1954253	0.23178176	0.87331541	33.5687732
ANXA8L2	ANXA8L2	-0.82791484	-0.643312	0.77702678	0.64024146	33.5687732
CAPN13	CAPN13	-0.82216164	-0.69635338	0.84697868	0.61713012	33.5687732
CEACAM6	CEACAM6	-0.81785184	-0.84811325	1.03700109	0.55551076	33.5687732
BYSL	BYSL	-0.81392929	-0.21449652	0.26353213	0.86184688	35.9964413
CHEK1	CHEK1	-0.79883143	-0.3525283	0.44130499	0.78321033	35.9964413
LRP8	LRP8	-0.79212518	-0.31660308	0.39968819	0.80295827	35.9964413
HN1	HN1	-0.77392654	-0.25091522	0.32421064	0.84036314	38.4879725
KRAS	KRAS	-0.76290103	-0.17625204	0.23102871	0.88499914	38.4879725
SUV39H2	SUV39H2	-0.7392045	-0.23045301	0.31175813	0.8523672	40.9868421
PROM1	PROM1	-0.67766158	-0.59598871	0.87947838	0.6615909	46.9648562
PSMA7	PSMA7	-0.67068882	-0.15203446	0.22668405	0.89998044	46.9648562
NQO1	NQO1	-0.65774019	-0.32820469	0.49898835	0.79652708	46.9648562
PSMD14	PSMD14	-0.65303937	-0.15751888	0.24120886	0.89656564	46.9648562
MRPS17	MRPS17	-0.64571493	-0.16534371	0.25606302	0.89171605	46.9648562
KRT6C	KRT6C	-0.64103347	-0.42019453	0.65549547	0.74732385	46.9648562
DEGS2	DEGS2	-0.62957468	-0.58762648	0.93337057	0.66543678	49.7147147
ORC6L	ORC6L	-0.61766333	-0.2932695	0.47480478	0.81605059	49.7147147
FZD7	FZD7	-0.61728916	-0.22975297	0.37219667	0.8527809	49.7147147
CELSR1	CELSR1	-0.61723203	-0.33403153	0.5411766	0.79331651	49.7147147
C8orf33	C8orf33	-0.61710639	-0.1845339	0.29903093	0.87993332	49.7147147
GPR160	GPR160	-0.59977579	-0.33058914	0.55118786	0.79521169	49.7147147
KRT5	KRT5	-0.59928673	-0.51342907	0.85673359	0.70055534	49.7147147
GPR89A	GPR89A	-0.58551239	-0.1400249	0.23914934	0.90750349	49.7147147
STK38L	STK38L	-0.57659453	-0.20186914	0.35010588	0.86942342	52.6988636
NAT1	NAT1	-0.56554082	-0.43738881	0.77339919	0.73846998	52.6988636
MAPT	MAPT	-0.56354485	-0.47200518	0.83756454	0.72096185	52.6988636
S100A8	S100A8	-0.55046667	-0.42654866	0.77488553	0.74403962	52.6988636
GALNT7	GALNT7	-0.54703525	-0.26089167	0.4769193	0.83457195	52.6988636
TMEM158	TMEM158	-0.54479481	-0.28216322	0.51792569	0.82235702	52.6988636
FGFR4	FGFR4	-0.53331401	-0.29737335	0.55759524	0.81373257	52.6988636
CCNE1	CCNE1	-0.53300048	-0.22433058	0.4208825	0.85599212	52.6988636
COX7B	COX7B	-0.52829224	-0.11825205	0.22383832	0.92130322	52.6988636
KRT14	KRT14	-0.51345386	-0.50740051	0.98821054	0.70348886	55.5050505
PNO1	PNO1	-0.51143952	-0.11856929	0.23183443	0.92110065	55.5050505
TMEM45B	TMEM45B	-0.51061067	-0.34800323	0.68154321	0.78567076	55.5050505

PCDH8	PCDH8	-0.50968405	-0.24837836	0.48731829	0.84184215	55.5050505
IFT74	IFT74	-0.50058954	-0.18272592	0.36502145	0.88103674	55.5050505
FGFR2	FGFR2	-0.50026238	-0.24668533	0.49311189	0.84283064	55.5050505
TM7SF3	TM7SF3	-0.4865095	-0.14320654	0.29435507	0.90550434	55.5050505
MIA	MIA	-0.48598116	-0.38965979	0.80180021	0.76330958	55.5050505
TMEM139	TMEM139	-0.48512429	-0.33325886	0.68695562	0.7937415	55.5050505
SPDEF	SPDEF	-0.48237982	-0.47528896	0.98530026	0.7193227	55.5050505
MTFR2	MTFR2	-0.48148516	-0.15230115	0.31631535	0.89981408	55.5050505
KRT17	KRT17	-0.4443962	-0.42899627	0.96534639	0.74277838	55.5050505
TSPAN13	TSPAN13	-0.43654459	-0.22536462	0.51624651	0.85537881	55.5050505
AGR3	AGR3	-0.43333797	-0.54367268	1.25461584	0.68602227	55.5050505
MIS18A	MIS18A	-0.43234053	-0.12659013	0.2928019	0.91599389	55.5050505
SNRPD1	SNRPD1	-0.43197156	-0.11138967	0.25786344	0.92569596	55.5050505
SCGB2A2	SCGB2A2	-0.42997571	-0.58929333	1.37052704	0.6646684	55.5050505
CENPI	CENPI	-0.42248161	-0.15054048	0.35632434	0.90091289	55.5050505
SPAG5	SPAG5	-0.40667303	-0.14311086	0.35190643	0.9055644	59.0069284
TP53	TP53	-0.39822236	-0.12340684	0.3098943	0.91801724	59.0069284
COX6C	COX6C	-0.39417571	-0.21692333	0.55032141	0.86039835	59.0069284
HRAS	HRAS	-0.39092956	-0.1109686	0.28385829	0.92596618	59.0069284
KRT6B	KRT6B	-0.39007387	-0.33113082	0.84889258	0.79491317	59.0069284
ELSPBP1	ELSPBP1	-0.38921988	-0.13874967	0.35648146	0.90830601	59.0069284
TMEM25	TMEM25	-0.37724937	-0.17022841	0.45123577	0.88870197	59.0069284
CCND1	CCND1	-0.37601518	-0.20547774	0.54646129	0.86725146	59.0069284
CTSV	CTSV	-0.37434891	-0.11480298	0.30667373	0.92350842	59.0069284
BTG3	BTG3	-0.3738895	-0.12491617	0.33409916	0.91705733	59.0069284
TRIM29	TRIM29	-0.36872086	-0.25829219	0.70050876	0.83607705	59.0069284
NFKBIE	NFKBIE	-0.36714931	-0.11187552	0.30471395	0.92538427	59.0069284
RARA	RARA	-0.36712694	-0.15363134	0.41846927	0.89898482	59.0069284
STC2	STC2	-0.36030955	-0.29609409	0.82177697	0.81445445	59.0069284
SLC16A3	SLC16A3	-0.35662998	-0.14327584	0.4017493	0.90546084	59.0069284
UBE2C	UBE2C	-0.35598275	-0.14528151	0.40811392	0.90420292	59.0069284
MYC	MYC	-0.35463433	-0.12530137	0.35332555	0.91681251	59.0069284
CDH3	CDH3	-0.3511379	-0.20274906	0.57740581	0.8688933	59.0069284
JUP	JUP	-0.34839316	-0.11986361	0.34404696	0.92027465	59.0069284
FANCA	FANCA	-0.336947	-0.15926604	0.47267387	0.89548052	59.0069284
IDH2	IDH2	-0.32471426	-0.10600035	0.32644194	0.92916045	60.7936508
BAG1	BAG1	-0.31855193	-0.10527977	0.33049485	0.92962465	60.7936508
GARS	GARS	-0.31639113	-0.07919982	0.25032251	0.94658252	60.7936508
GPSM2	GPSM2	-0.3149927	-0.12304549	0.39062967	0.91824721	60.7936508
NUDCD1	NUDCD1	-0.31165928	-0.1469671	0.47156336	0.90314711	60.7936508
MLPH	MLPH	-0.29599618	-0.25781195	0.87099756	0.83635541	62.6865672
KIAA0040	KIAA0040	-0.29501977	-0.1279075	0.43355569	0.91515784	62.6865672
CD44	CD44	-0.28065455	-0.090585	0.32276336	0.93914186	62.6865672
MIEN1	MIEN1	-0.27427787	-0.07454776	0.27179648	0.94963976	64.1422594
HSPA14	HSPA14	-0.2638142	-0.07431554	0.28169653	0.94979262	64.1422594
LRIG1	LRIG1	-0.24890408	-0.09791045	0.3933662	0.93438534	65.1975052
ZNF217	ZNF217	-0.24298163	-0.08897184	0.36616695	0.94019255	65.1975052
KNTC2	KNTC2	-0.23701056	-0.09729871	0.41052478	0.93478163	65.1975052
CXCL8	CXCL8	-0.22945805	-0.16486452	0.71849523	0.89201229	65.1975052
MAP2K4	MAP2K4	-0.22856751	-0.06092335	0.26654421	0.95865037	65.1975052

PCNA	PCNA	-0.22581572	-0.06542421	0.28972389	0.95566427	65.1975052
XBP1	XBP1	-0.22078697	-0.11329428	0.51313844	0.92447468	65.1975052
BLM	BLM	-0.20362369	-0.08001437	0.39295219	0.94604822	65.1975052
ASUN	ASUN	-0.20114473	-0.05331433	0.26505456	0.96371981	66.2106299
PGR	PGR	-0.19764194	-0.17125419	0.86648711	0.88807031	66.2106299
ACTL8	ACTL8	-0.19712856	-0.11408692	0.57874375	0.9239669	66.2106299
SLC9A3R1	SLC9A3R1	-0.19648309	-0.1324667	0.6741888	0.91227033	66.2106299
PHGDH	PHGDH	-0.19250224	-0.10292248	0.53465602	0.93114485	66.2106299
RAF1	RAF1	-0.1915807	-0.03923341	0.20478788	0.97317192	66.2106299
MCM3	MCM3	-0.18033806	-0.05068813	0.28107285	0.96547571	66.2106299
AGR2	AGR2	-0.17702579	-0.19600486	1.10721075	0.87296465	66.2106299
BRCA2	BRCA2	-0.17546437	-0.06933573	0.3951556	0.95307673	66.2106299
CENPN	CENPN	-0.17232757	-0.04832093	0.2804016	0.96706118	66.2106299
DNAJC12	DNAJC12	-0.16764667	-0.12870483	0.76771481	0.9146522	66.2106299
RERG	RERG	-0.16288916	-0.10037884	0.61624015	0.93278802	66.2106299
PIP	PIP	-0.1621952	-0.2160424	1.33199006	0.86092389	66.2106299
NFKBIB	NFKBIB	-0.15989575	-0.03659429	0.22886341	0.97495377	66.2106299
AURKA	AURKA	-0.15772331	-0.05013887	0.3178913	0.96584336	66.2106299
FOXA1	FOXA1	-0.15560174	-0.18821771	1.20961183	0.87768934	66.2106299
IL6	IL6	-0.15374749	-0.07748856	0.50399885	0.94770598	66.2106299
KRT16	KRT16	-0.14325124	-0.1157764	0.80820524	0.92288552	66.5686275
ESR1	ESR1	-0.14038445	-0.13843939	0.98614478	0.90850138	66.5686275
WDR4	WDR4	-0.0999099	-0.02729449	0.27319099	0.98125875	67.8502879
CHEK2	CHEK2	-0.09919629	-0.02800008	0.28226938	0.98077895	67.8502879
GSTM3	GSTM3	-0.09123375	-0.05974873	0.6548972	0.95943121	67.8502879
PYROXD1	PYROXD1	-0.08722976	-0.02203533	0.2526125	0.98484233	67.8502879
REEP6	REEP6	-0.08502067	-0.06950051	0.81745429	0.95296788	67.8502879
PLA1A	PLA1A	-0.08192494	-0.03378909	0.41243957	0.97685133	67.8502879
MMP11	MMP11	-0.08090333	-0.05017856	0.62022861	0.96581678	67.8502879
ERBB4	ERBB4	-0.07852237	-0.07570974	0.96418052	0.9488752	67.8502879
PTDSS1	PTDSS1	-0.07012406	-0.01749539	0.24949195	0.98794636	67.8502879
RACGAP1	RACGAP1	-0.06998841	-0.01844008	0.26347331	0.98729965	67.8502879
INPP4B	INPP4B	-0.06994693	-0.02934336	0.41950884	0.97986618	67.8502879
CXXC5	CXXC5	-0.06213789	-0.02544854	0.40954944	0.98251508	67.8502879
GATA3	GATA3	-0.04147902	-0.02997764	0.72271824	0.97943548	67.8502879
AHCYL1	AHCYL1	-0.03435752	-0.00803669	0.23391358	0.99444488	67.8502879
MGC18216	MGC18216	-0.03327079	-0.01807944	0.54340279	0.98754648	67.8502879
S100A9	S100A9	-0.03137577	-0.02622728	0.83590882	0.98198488	67.8502879
TCEAL1	TCEAL1	-0.02960323	-0.01420028	0.47968687	0.9902054	67.8502879
TFAM	TFAM	-0.01378364	-0.00364679	0.26457355	0.99747543	67.8502879
FAM174B	FAM174B	-0.0124089	-0.00599938	0.48347439	0.99585018	67.8502879
BRCA1	BRCA1	-0.0104649	-0.0038416	0.36709337	0.99734075	67.8502879
MYO5C	MYO5C	-0.00958001	-0.00418495	0.43684178	0.99710342	67.8502879
PSPH	PSPH	-0.00361096	-0.00088873	0.24611942	0.99938417	67.8502879

Supplementary Table 3. Gene expression ratio between C2D1 and baseline samples with pathological complete response (pCR) and non pCR. Lists of differentially expressed genes ratio (C2D1/baseline) between with pCR and non pCR tumors determined by SAM analysis.

Gene ID	Score(d)	Numerator(r)	Denominator(	FoldChange	q-value(%)
CAV1	3.75919976	1.70208626	0.45277888	3.25371134	0
RAI2	3.32835416	1.4910677	0.4479895	2.81096931	0
MME	3.22120647	1.82956986	0.56797659	3.55431085	0
ADM	3.14150944	1.67015935	0.53164232	3.18249744	0
TWIST1	3.13543425	1.48843116	0.47471292	2.80583691	0
MAP7D3	2.89037846	0.91104952	0.31520077	1.88041295	0.50039401
CYBRD1	2.88461117	1.24710565	0.43233059	2.37364741	0.50039401
OGN	2.88098218	2.04150823	0.70861536	4.11675681	0.50039401
CXCR1	2.8529572	1.18846853	0.41657426	2.2791068	0.50039401
F3	2.82074767	1.03226522	0.36595447	2.04523302	0.50039401
AKT3	2.80382245	0.97610463	0.34813354	1.9671468	0.50039401
CDKN2C	2.77976837	0.94260442	0.33909459	1.92199478	0.50039401
RECK	2.77802146	1.06303974	0.38266074	2.08932907	0.50039401
C16orf45	2.7566051	1.07116143	0.38857993	2.10112418	0.50039401
PID1	2.49333533	1.00653002	0.40368819	2.00907304	0.77747169
PDGFRA	2.48626468	0.95512546	0.38416081	1.93874822	0.77747169
ZEB1	2.47658907	0.87721357	0.35420231	1.83682422	0.77747169
NT5E	2.47495869	0.94540333	0.38198752	1.92572718	0.77747169
CBX7	2.47395183	0.87552968	0.35389924	1.83468156	0.77747169
EGFR	2.42738352	1.03213123	0.4252032	2.04504308	0.77747169
GUSB	2.41179101	0.60577333	0.25117157	1.52179426	0.77747169
AXL	2.36680499	0.70341442	0.29719999	1.62835405	1.02254428
ANXA1	2.35354429	0.82459117	0.35036144	1.77103309	1.02254428
VIM	2.32180139	0.72062032	0.31037122	1.64789043	1.02254428
CRYAB	2.31847573	1.28460768	0.55407424	2.43615795	1.02254428
TWIST2	2.31198546	1.12043159	0.48461879	2.17412003	1.02254428
FABP5	2.27927984	0.8999281	0.39483002	1.86597298	1.02254428
SLC9A3	2.25321353	0.87504341	0.38835352	1.83406328	1.25432099
CYR61	2.25187838	0.83788378	0.37208216	1.78742633	1.25432099
ME1	2.2387919	0.7615626	0.34016677	1.69532586	1.25432099
GAL	2.15379742	1.03915234	0.48247451	2.05501987	2.05787037
PTGS2	2.14497488	0.91926465	0.42856663	1.89115111	2.05787037
GNG11	2.13083088	0.99293545	0.4659851	1.99023039	2.05787037
TGFBR3	2.12741334	1.07246647	0.50411758	2.10302568	2.05787037
TUBB6	2.11155805	0.65714189	0.31121185	1.57695544	2.05787037
RRAGD	2.094272	0.77730128	0.37115584	1.71392179	2.05787037
MAGOHB	2.03552552	0.52487641	0.25785794	1.43881032	2.05787037
CD44	2.01522481	0.51090544	0.2535228	1.42494422	2.68783069
IL6ST	2.00397384	0.69372628	0.34617532	1.61745579	2.68783069
ALDH1A1	1.98397706	1.00699316	0.50756291	2.00971812	2.68783069
UCHL1	1.97872724	0.76170634	0.38494762	1.69549477	2.68783069
KIT	1.96876831	1.03892379	0.52770241	2.05469434	2.68783069
MPP1	1.96007997	0.66598498	0.33977439	1.58665117	2.68783069
LHFP	1.95854144	0.74931781	0.38258971	1.68099777	2.68783069

PPFIBP1	1.91353981	0.48325592	0.25254553	1.39789492	2.68783069
SNAI1	1.91273423	0.62209602	0.32523913	1.53910965	2.68783069
P3H1	1.89455239	0.50795902	0.26811558	1.42203702	2.68783069
A1CF	1.88458183	0.87890531	0.46636622	1.83897939	2.68783069
SPATA7	1.86847183	0.51616764	0.27625123	1.43015116	2.68783069
CXCR2	1.86202989	0.77423108	0.41579949	1.71027827	2.68783069
KIF20A	1.84827282	0.70864313	0.38340829	1.63426635	2.68783069
GSTM4	1.84084629	0.49833087	0.27070748	1.41257833	2.68783069
MLKL	1.83844851	0.53441657	0.2906889	1.44835632	2.68783069
H19	1.81488086	0.90427611	0.49825646	1.87160516	3.32809224
FIGF	1.80843704	1.19772376	0.66229774	2.29377481	3.32809224
FABP4	1.79842155	1.88246062	1.04672935	3.68703374	3.32809224
CA12	1.78978089	0.84315735	0.47109529	1.79397198	3.32809224
ZEB2	1.78132276	0.73326925	0.41164312	1.66240195	3.32809224
KRT16	1.76161014	1.11212292	0.63131047	2.16163497	3.32809224
LAMA3	1.7503565	0.735999	0.4204852	1.66555038	3.32809224
SLC7A6	1.74724814	0.46894066	0.26838813	1.38409278	3.32809224
LOC400043	1.74428706	0.55612985	0.31882932	1.47031967	3.32809224
LRRC2	1.74336673	0.70515723	0.40448015	1.63032234	3.32809224
GSTM3	1.73702881	0.66683139	0.38389196	1.58758231	3.32809224
YBX3	1.73495993	0.47997769	0.27665059	1.3947221	3.32809224
ADRA2A	1.72737301	0.79632322	0.46100247	1.73666949	3.32809224
ABCC3	1.72652904	0.58592146	0.3393638	1.50099738	3.32809224
APH1B	1.72104364	0.52090834	0.30267004	1.43485837	3.32809224
NUDCD1	1.7164621	0.66860968	0.38952778	1.58954039	3.32809224
NOTCH2	1.70899728	0.37397025	0.21882437	1.29591424	3.32809224
STC2	1.70333576	1.06985236	0.62809247	2.09921853	3.32809224
CRIM1	1.69503397	0.5499229	0.32443178	1.46400745	3.32809224
THY1	1.69333852	0.61765837	0.36475776	1.53438271	3.32809224
FBN1	1.69321513	0.66424194	0.39229625	1.58473536	3.32809224
TMEM158	1.68923765	0.79019261	0.4677806	1.72930532	3.32809224
PSPHL	1.67496092	0.69572069	0.41536533	1.61969334	3.32809224
KRT6A	1.6685064	0.71060575	0.42589333	1.6364911	3.32809224
ANGPTL4	1.66246764	1.03882833	0.62487131	2.05455839	3.32809224
KLHL9	1.65118529	0.34926713	0.2115251	1.27391333	4.16256965
DNALI1	1.64927955	0.69393142	0.42074821	1.6176858	4.16256965
NFIA	1.624637	0.51425502	0.31653534	1.42825643	4.16256965
SEMA3C	1.58473509	0.64295323	0.40571653	1.56152236	4.16256965
FAP	1.58006315	0.66207166	0.41901595	1.58235319	4.16256965
COG8	1.57574761	0.33707186	0.21391234	1.26319018	4.16256965
CD68	1.55542805	0.65272779	0.41964512	1.57213793	4.80169753
EMP3	1.54241043	0.51063662	0.33106403	1.42467873	4.80169753
SHC1	1.53955915	0.32201015	0.20915738	1.2500711	4.80169753
ST18	1.53136182	0.60480038	0.39494284	1.52076832	4.80169753
KDR	1.52819282	0.46195254	0.30228682	1.37740473	4.80169753
PTEN	1.52704146	0.34997354	0.22918404	1.27453725	4.80169753
RB1	1.5166737	0.33406125	0.22025915	1.26055691	4.80169753
PGR	1.5026683	0.86286935	0.57422476	1.8186518	4.80169753
GLRB	1.50154271	0.59211926	0.39434061	1.50745952	4.80169753
FAM171A1	1.47014137	0.51358069	0.34934102	1.427589	4.80169753

NDRG1	1.45837541	0.63313553	0.43413755	1.5509321	4.80169753
SMO	1.45575782	0.45520314	0.31269153	1.37097583	4.80169753
GREM1	1.426586	0.66969728	0.46944053	1.59073915	6.45606391
IGF1	1.42063855	0.85187238	0.59964048	1.80484179	6.45606391
SCGB2A2	1.40440312	1.42701598	1.0161014	2.68889977	6.45606391
MAP2K1	1.40144635	0.26955275	0.19233898	1.20543408	6.45606391
NDUFAF4	1.3885045	0.38209753	0.27518639	1.30323525	6.45606391
SCUBE2	1.38764052	0.7248148	0.52233615	1.65268847	6.45606391
ERCC1	1.364732	0.34876971	0.25555912	1.27347418	6.45606391
PLOD1	1.36401465	0.34190686	0.25066216	1.26743069	6.45606391
CLMN	1.36060542	0.46582667	0.34236721	1.38110851	6.45606391
PIK3CA	1.33754585	0.30493415	0.22798034	1.23536225	7.77989016
ID4	1.31389965	0.60195447	0.45814341	1.51777135	7.77989016
KLF4	1.29820113	0.4773759	0.36772107	1.39220909	7.77989016
RAD51B	1.29281948	0.33974956	0.26279737	1.26553689	7.77989016
GSTM1	1.28292591	0.52543531	0.40956014	1.43936782	7.77989016
AHCYL1	1.27543219	0.23395314	0.18343048	1.17605305	7.77989016
MET	1.27442587	0.5825709	0.4571242	1.49751547	7.77989016
CFLAR	1.24657127	0.30752422	0.24669606	1.23758209	8.81944444
ANXA8L2	1.23485125	0.83823635	0.67881565	1.7878632	8.81944444
IL6R	1.21862939	0.41179476	0.33791632	1.33033977	8.81944444
CCND2	1.21479478	0.46192432	0.38024885	1.37737779	8.81944444
NTN4	1.1992941	0.64259419	0.53581035	1.5611338	8.81944444
TGFBR2	1.16177877	0.41069491	0.35350526	1.32932596	10.8927875
OGFRL1	1.15942449	0.33795154	0.29148215	1.26396064	10.8927875
CAPN6	1.15729971	0.86468845	0.74716034	1.82094639	10.8927875
CHST11	1.1238425	0.35416236	0.31513522	1.2782432	10.8927875
KRTAP1.1	1.12117263	0.4424761	0.39465475	1.35893467	12.9544627
NOTCH3	1.10390471	0.30562605	0.27685908	1.23595486	12.9544627
FNBP1	1.09791099	0.25357141	0.23095808	1.19215466	12.9544627
SERPINA3	1.09416446	0.63779606	0.58290694	1.55595039	12.9544627
ITGB1	1.09024227	0.27560989	0.25279692	1.21050571	12.9544627
FZD7	1.07272479	0.38232736	0.35640769	1.30344288	12.9544627
SLC40A1	1.06992344	0.56277696	0.52599741	1.47710968	12.9544627
FOXC1	1.06429121	0.45640008	0.42883007	1.37211374	12.9544627
TSHZ1	1.06028745	0.31434787	0.2964742	1.24344946	12.9544627
FGFR1	1.05418582	0.41256914	0.39136282	1.33105404	12.9544627
CTGF	1.05054681	0.4110652	0.39128689	1.3296672	12.9544627
KDM4B	1.04297837	0.29300205	0.28092821	1.22518707	12.9544627
EIF2S2	1.02931977	0.33785021	0.32822668	1.26387187	15.2906777
BDNF	1.02909189	0.34781888	0.33798622	1.27263516	15.2906777
NEO1	1.02886177	0.29786631	0.28951053	1.22932495	15.2906777
CDKN1B	1.02269921	0.28509805	0.27877019	1.21849307	15.2906777
PRAME	1.01795363	0.58989537	0.5794914	1.50513759	15.2906777
ACOT4	1.00757952	0.30517349	0.30287783	1.23556721	15.2906777
CXCL8	0.99899029	0.64595494	0.64660783	1.56477469	15.2906777
CTSL	0.98490211	0.3477673	0.35309834	1.27258965	15.2906777
AVEN	0.97652846	0.21861187	0.22386635	1.16361344	15.2906777
SH2B3	0.96442601	0.27674412	0.28695215	1.21145777	15.2906777
CHUK	0.96001127	0.1807903	0.18832102	1.13350464	15.2906777

MAPT	0.93952261	0.4865735	0.5178944	1.40111318	16.9618406
TCF7L1	0.93187582	0.3472499	0.37263538	1.27213334	16.9618406
ELOVL5	0.93070955	0.30682005	0.32966252	1.23697818	16.9618406
ACTR3B	0.9183541	0.23799666	0.25915566	1.17935386	16.9618406
LTBP2	0.90296064	0.32410166	0.35893221	1.25188467	16.9618406
RRP15	0.89904717	0.23553126	0.26197875	1.1773402	16.9618406
CYCS	0.89433635	0.19856152	0.22202108	1.14755359	16.9618406
BRAF	0.87841313	0.1908766	0.21729707	1.14145707	19.3480836
NF1	0.8707542	0.21125666	0.24261342	1.15769615	19.3480836
SMIM14	0.86157831	0.25816392	0.29964069	1.19595567	19.3480836
RGS22	0.85319214	0.41646511	0.48812582	1.33465338	19.3480836
PIR	0.84802009	0.26088105	0.30763546	1.19821023	19.3480836
LOC389332	0.84744472	0.39420955	0.46517435	1.3142225	19.3480836
IKBKB	0.80401533	0.18418687	0.22908377	1.13617642	20.7360459
ABCB1	0.80354921	0.40179004	0.50001921	1.32114612	20.7360459
CAMK2N1	0.79320522	0.28279857	0.35652636	1.21655249	20.7360459
RAD50	0.79052567	0.16891372	0.21367266	1.12421169	20.7360459
BAG1	0.78593855	0.1971087	0.25079403	1.14639856	20.7360459
MMP11	0.7831514	0.49267446	0.62909224	1.40705085	20.7360459
CXCL1	0.78164217	0.45142958	0.57753995	1.36739455	20.7360459
KIF13B	0.7803825	0.23044997	0.29530386	1.17320081	20.7360459
GSTP1	0.775397	0.23632448	0.30477868	1.1779877	20.7360459
BCL2	0.76847257	0.27874148	0.36272145	1.21313616	20.7360459
CTNNB1	0.74288142	0.18160753	0.24446368	1.13414691	20.7360459
PEX11G	0.74074588	0.19636482	0.26509066	1.1458076	20.7360459
RINT1	0.73556104	0.14372664	0.1953973	1.10475514	20.7360459
KRT14	0.72513893	0.70213358	0.96827457	1.62690903	23.0674784
APC	0.71416795	0.16114704	0.22564305	1.11817581	23.0674784
NOTCH1	0.69969517	0.20042172	0.28644148	1.14903418	23.0674784
AFF3	0.69790965	0.32308331	0.46292999	1.25100132	23.0674784
PYROXD1	0.69639657	0.14962636	0.21485798	1.10928214	23.0674784
INPP4B	0.69354645	0.24375133	0.35145639	1.18406751	23.0674784
STAT3	0.68575826	0.12996472	0.18951972	1.09426694	23.0674784
HEXIM1	0.6767966	0.15533619	0.2295168	1.11368111	23.0674784
AARS	0.67355138	0.13799411	0.2048754	1.10037411	23.0674784
RAD17	0.67320978	0.15900866	0.23619481	1.11651966	23.0674784
CDC25B	0.66862181	0.17903308	0.26776434	1.13212486	23.0674784
CDCA7L	0.65975778	0.20660915	0.3131591	1.15397275	23.0674784
RELA	0.65974756	0.12704834	0.19257114	1.09205714	23.0674784
SFRP1	0.65697841	0.48499073	0.7382141	1.39957687	23.0674784
MTHFD1L	0.65033793	0.17852153	0.27450579	1.1317235	26.1978434
PIK3R1	0.64106299	0.20249565	0.31587481	1.15068715	26.1978434
ABCC8	0.63520336	0.36173959	0.56948626	1.28497437	26.1978434
STMN1	0.61579313	0.20023389	0.32516421	1.1488846	26.1978434
C1orf21	0.61549974	0.23591065	0.3832831	1.17764985	26.1978434
MYC	0.61312082	0.1936794	0.31589108	1.14367679	26.1978434
PTGER4	0.57224773	0.20304072	0.35481262	1.15112198	28.1046296
NFKBIB	0.55552072	0.11843203	0.21319102	1.0855544	28.1046296
IGBP1	0.55522204	0.13897163	0.2502992	1.10111995	28.1046296
NFIB	0.52808442	0.2164202	0.40982122	1.16184708	29.8810163

PSPH	0.52592659	0.11754176	0.22349462	1.08488473	29.8810163
FAM214A	0.52392337	0.17840442	0.34051624	1.13163164	29.8810163
PUM1	0.52303225	0.09465107	0.18096602	1.06780712	29.8810163
CDKN2B	0.52275836	0.17651624	0.33766315	1.13015154	29.8810163
RECQL	0.50616612	0.11750799	0.232153	1.08485933	29.8810163
ABAT	0.48038652	0.17432131	0.36287719	1.12843343	32.2290809
RHBG	0.4730627	0.1649482	0.34868147	1.12112582	32.2290809
BCL11A	0.46826806	0.17763595	0.37934672	1.13102902	32.2290809
CHPF	0.46743965	0.1453076	0.31085853	1.10596644	32.2290809
FZD6	0.46730082	0.13152477	0.28145632	1.09545086	32.2290809
MGC18216	0.4478133	0.15943331	0.3560263	1.11684836	33.2910536
KIAA0040	0.42134922	0.14603962	0.34659996	1.10652774	35.0399501
SETBP1	0.4158219	0.13283021	0.31944015	1.09644254	35.0399501
CD86	0.39532093	0.14196271	0.3591075	1.10340522	35.0399501
BTG3	0.37363319	0.09458596	0.25315193	1.06775893	36.5958486
NACC2	0.37230704	0.08853448	0.23779964	1.06328952	36.5958486
STK11	0.37035262	0.08048607	0.21732281	1.05737423	36.5958486
ELSPBP1	0.35802616	0.15183	0.42407514	1.11097781	36.5958486
NCS1	0.34586536	0.08743433	0.25279875	1.062479	36.5958486
GOLT1A	0.3302596	0.13704185	0.4149519	1.09964805	38.3044226
DDB2	0.32795357	0.08900259	0.27138777	1.06363458	38.3044226
PDXK	0.32398341	0.06554741	0.20231717	1.04648194	38.3044226
ACTL8	0.32082029	0.16094446	0.50166548	1.11801881	38.3044226
C4orf32	0.31840671	0.1141471	0.35849465	1.082335	38.3044226
PPP6R1	0.31287492	0.06413579	0.20498859	1.0454585	38.3044226
AGR2	0.31261532	0.1802001	0.57642762	1.13304103	38.3044226
KRT5	0.3087583	0.24294475	0.78684442	1.1834057	38.3044226
KRT17	0.30645128	0.28239824	0.92151103	1.21621496	38.3044226
IGF2R	0.30634136	0.06845902	0.223473	1.04859605	38.3044226
CXCL14	0.30221911	0.17576092	0.58156784	1.12956001	38.3044226
FLVCR2	0.30123077	0.09351482	0.31044245	1.06696645	38.3044226
ITGA6	0.28058417	0.08541054	0.30440256	1.06098962	38.3044226
IL1B	0.26687718	0.11215457	0.42024788	1.0808412	39.8750191
ITCH	0.26514302	0.05106545	0.19259585	1.03602976	39.8750191
NFKB1	0.25735317	0.06486016	0.25202781	1.04598355	39.8750191
EVI2A	0.25105997	0.09307881	0.37074332	1.06664405	40.583785
WIPF2	0.22522898	0.0440146	0.19542157	1.03097875	41.8212366
BMI1	0.20365276	0.05620073	0.27596349	1.03972408	41.8212366
AZGP1	0.20205753	0.13498523	0.66805345	1.09808157	41.8212366
ADRA2C	0.19598366	0.10362441	0.52874007	1.07446941	42.8948322
LRIG1	0.19305864	0.05143424	0.26641772	1.03629464	42.8948322
COX6C	0.18991104	0.06610016	0.34805853	1.04688296	42.8948322
RACGAP1	0.17835302	0.05190572	0.29102798	1.03663335	42.8948322
THBS1	0.17068361	0.0615488	0.36060168	1.0435855	42.8948322
KCNJ15	0.16821273	0.06237408	0.37080475	1.04418264	42.8948322
KRAS	0.1675157	0.03377705	0.20163515	1.02368869	42.8948322
KLHL7	0.15936315	0.03740387	0.23470842	1.0262654	42.8948322
FAM198B	0.15269372	0.05774001	0.37814268	1.04083401	42.8948322
INSIG1	0.13670345	0.03477602	0.25439023	1.02439777	43.3978079
RNF103	0.13655049	0.02958139	0.21663333	1.02071591	43.3978079

MAGEA1	0.13261437	0.06638862	0.50061406	1.0470923	43.3978079
MIA	0.13114599	0.07013801	0.53480863	1.04981711	43.3978079
HIF1A	0.12534833	0.03067197	0.24469387	1.0214878	43.4571469
BLVRA	0.12196026	0.03149265	0.25822061	1.02206904	43.4571469
KPNA1	0.10539366	0.01824048	0.17307002	1.0127236	43.8054849
TP63	0.10388728	0.06982288	0.67210228	1.04958781	43.8054849
TRIP13	0.09994904	0.03341693	0.3343397	1.02343319	43.8054849
PTDSS1	0.09236802	0.02037666	0.22060296	1.01422424	43.8054849
LOC642077	0.09013926	0.02944142	0.32662148	1.02061689	43.8054849
NFKBIA	0.07175371	0.01828791	0.25487053	1.01275689	44.1754682
PIP	0.06827572	0.0700672	1.02623881	1.04976558	44.1754682
KRT6B	0.06298889	0.04543448	0.72130947	1.03199393	44.1754682
NPEPPS	0.05502546	0.01073013	0.19500296	1.00746528	44.4535634
NRAS	0.03362053	0.00636927	0.18944571	1.0044246	44.4535634
TFF3	0.02356689	0.01698602	0.7207578	1.01184339	44.4535634
RERG	0.01929954	0.00783106	0.40576409	1.00544284	44.4535634
VEGFA	0.01139462	0.00465186	0.40825019	1.00322963	44.4535634
EPCAM	-4.1691525	-2.00108993	0.47997523	0.2498112	0
LOC647867	-3.82981338	-1.90346964	0.49701368	0.26729974	0
CDH1	-3.7318194	-1.65262983	0.44284829	0.31805985	0
CLDN4	-3.47142807	-1.59456098	0.45933862	0.33112298	0
KRT8	-3.42421908	-1.45041159	0.42357441	0.36591702	0
CLDN3	-3.41831363	-1.64451453	0.48108942	0.31985401	0
ESRP1	-3.3811441	-1.50730503	0.44579734	0.35176771	0
KRT18	-3.31388223	-1.42578044	0.43024475	0.37221796	0
RRM2	-3.27082078	-1.45165707	0.44382043	0.36560126	0
SPINT1	-3.26165595	-1.16828022	0.35818622	0.44495143	0
VAV3	-3.2471204	-1.15892315	0.35690797	0.44784669	0
GRHL2	-3.22689084	-1.40235022	0.43458248	0.37831235	0
E2F1	-3.22113411	-1.23553799	0.38357235	0.4246841	0
GRHL1	-3.14717278	-1.31857321	0.41897071	0.40093126	0
GINS2	-3.0847052	-1.12920278	0.36606506	0.45716828	0
MYBL2	-3.08192179	-1.43082224	0.46426299	0.37091943	0
SPAG5	-3.05220946	-0.95903284	0.31420938	0.51440165	0
KIAA1324	-3.00267844	-1.61869108	0.53908239	0.32563077	0
KRT19	-2.99883331	-1.68913848	0.56326521	0.31011206	0
OCLN	-2.96993453	-1.29083437	0.43463395	0.40871458	0
DLGAP5	-2.91788481	-1.12762941	0.38645439	0.45766713	0
TYMS	-2.91047475	-1.11376155	0.3826735	0.46208765	0
CCNB1	-2.90526618	-1.13575433	0.39092952	0.4550969	0
RAB25	-2.87740849	-1.53323375	0.53285231	0.34550207	0
KIFC1	-2.87287317	-1.26635456	0.44079724	0.41570887	0
HN1	-2.87253884	-0.77068823	0.26829515	0.58613779	0
BIRC5	-2.8602732	-1.31626252	0.46018769	0.40157392	0
GRB7	-2.83757366	-1.06785934	0.37632832	0.47702628	0
CDT1	-2.8332505	-1.00543028	0.35486812	0.49812155	0
SPINT2	-2.79638073	-0.88992285	0.31824095	0.53964298	0
TOP2A	-2.77063706	-1.16298447	0.41975345	0.44658773	0
UBE2T	-2.7444742	-1.08855624	0.39663562	0.47023172	0
CHEK1	-2.6796821	-0.90485228	0.3376715	0.53408739	0

SPDEF	-2.67531913	-1.61294079	0.6028966	0.32693125	0
CDC45	-2.6715367	-1.02747624	0.38460121	0.49056757	0
EZH2	-2.66954105	-0.8394844	0.31446769	0.55884326	0
FGFR4	-2.62221344	-1.16019804	0.44244989	0.44745111	0
CDC6	-2.61148163	-0.8743242	0.33480006	0.54550934	0
TSPAN13	-2.59776999	-0.85586371	0.32946093	0.55253444	0
FANCA	-2.57225445	-0.88311051	0.3433216	0.54219717	0
DDR1	-2.56090745	-0.96460694	0.37666607	0.512418	0
CDK1	-2.55529969	-1.00660492	0.39392832	0.49771614	0
PRC1	-2.55242458	-0.93662043	0.36695323	0.52245532	0
HRAS	-2.52667431	-0.50340632	0.19923673	0.70543921	0
RFC4	-2.51802514	-0.61872176	0.24571707	0.65124768	0
CDC25C	-2.49526008	-0.80609071	0.32304878	0.57192952	0
C1orf106	-2.49261441	-1.1772763	0.47230582	0.44218552	0
UBE2C	-2.47914268	-0.83673104	0.33750822	0.55991082	0
GPR160	-2.47911888	-0.84025232	0.33893184	0.55854587	0
TOM1L1	-2.47748784	-0.92666886	0.37403569	0.52607162	0
RAD51	-2.43128329	-0.83269888	0.34249357	0.56147789	0
HJURP	-2.40623037	-0.97604471	0.40563228	0.50837158	0
ERBB3	-2.40084433	-0.92418849	0.38494311	0.52697686	0
CAPN13	-2.39830964	-1.27474327	0.53151739	0.4132987	0
MELK	-2.38328612	-0.80232966	0.33664848	0.57342247	0
TK1	-2.37777138	-0.78455481	0.32995385	0.58053107	0
CENPI	-2.35394794	-0.73036214	0.31027115	0.60275259	0
KIF4A	-2.35203613	-0.95376641	0.4055067	0.51628286	0
PITX1	-2.35133915	-1.2527585	0.53278511	0.41964506	0
ORC6L	-2.34870855	-0.86857024	0.36980759	0.54768936	0
EPN3	-2.34596276	-1.03084032	0.43941035	0.48942499	0
CDCA5	-2.34462862	-1.01249189	0.43183465	0.49568933	0
CLDN7	-2.32023086	-0.97454228	0.42001953	0.50890128	0
FOXM1	-2.31405849	-1.12624963	0.48669886	0.45810505	0
BUB1	-2.28917588	-0.87601788	0.38267828	0.5448693	0
HSPD1	-2.28128752	-0.55518222	0.24336355	0.68057109	0
TTK	-2.27963416	-0.95072433	0.41705128	0.51737264	0
MSH2	-2.27585726	-0.55353998	0.24322263	0.68134624	0
SLC9A3R1	-2.2727731	-0.91581044	0.40294847	0.53004603	0
LSR	-2.21025729	-0.75955921	0.34365194	0.59067677	0
KIF23	-2.20653724	-0.85344667	0.38678099	0.55346091	0
CEACAM6	-2.19731605	-1.58475601	0.72122352	0.33338105	0
PGAM5	-2.19279314	-0.49135022	0.22407504	0.71135903	0
SRC	-2.18897065	-0.60894059	0.27818582	0.65567801	0
IDH2	-2.18769221	-0.5910479	0.27016959	0.66386054	0
MCM2	-2.18621346	-0.63298515	0.28953493	0.64484076	0
CKS2	-2.16219079	-0.75831672	0.35071684	0.5911857	0
KIF2C	-2.12358406	-0.83721629	0.39424683	0.55972252	0.50039401
BLM	-2.12356157	-0.68653872	0.32329589	0.62134277	0.50039401
CEP55	-2.11030813	-0.92066851	0.43627208	0.52826418	0.50039401
CDKN3	-2.06234403	-0.6759267	0.32774682	0.62593003	0.50039401
ANLN	-2.0596009	-0.87559437	0.42512817	0.54502928	0.50039401
KCTD1	-2.05130236	-0.61465011	0.29963896	0.65308827	0.50039401

PHGDH	-2.04907276	-0.77057373	0.37605972	0.58618431	0.50039401
SLC5A6	-2.04221423	-0.63900507	0.31289816	0.64215565	0.50039401
PTTG1	-2.02898615	-0.69607343	0.34306466	0.61724989	0.50039401
PSMA7	-2.02620228	-0.4209289	0.20774278	0.74694354	0.50039401
F11R	-2.01290054	-0.52753262	0.26207585	0.6937402	0.50039401
SUV39H2	-2.00094934	-0.53181202	0.26577985	0.69168544	0.50039401
CENPA	-2.00021012	-0.80398535	0.40195045	0.57276476	0.50039401
HMGA1	-1.97863828	-0.62473785	0.31574131	0.64853761	0.50039401
PCNA	-1.97851412	-0.49823854	0.2518246	0.70797065	0.50039401
CKS1B	-1.93627103	-0.52427215	0.27076383	0.69530981	0.50039401
MAD2L1	-1.92564516	-0.60683629	0.31513401	0.65663507	0.50039401
MIS18A	-1.9054391	-0.42939671	0.22535315	0.74257224	0.77747169
TOMM40	-1.90484963	-0.4434028	0.23277575	0.73539802	0.77747169
CAND1	-1.89697634	-0.34759946	0.18323869	0.78589067	0.77747169
ZNF217	-1.89380903	-0.46927663	0.24779512	0.72232668	0.77747169
PSMD14	-1.89034377	-0.39954729	0.21136224	0.75809614	0.77747169
CENPF	-1.88826496	-0.89082621	0.47176971	0.53930518	0.77747169
FBXL6	-1.87591435	-0.52995502	0.28250491	0.69257633	0.77747169
GGH	-1.86314256	-0.65813256	0.3532379	0.63369803	0.77747169
CDCA8	-1.83114194	-0.6683484	0.36498994	0.62922662	0.77747169
MKI67	-1.83072554	-0.75650334	0.41322597	0.59192925	0.77747169
CDC20	-1.81493744	-0.77680324	0.42800552	0.58365865	0.77747169
CENPN	-1.81281886	-0.47639447	0.2627921	0.71877171	0.77747169
TACC3	-1.78613006	-0.63918515	0.35786036	0.6420755	1.02254428
MLPH	-1.78547261	-0.77569078	0.43444563	0.58410888	1.02254428
SQLE	-1.78378235	-0.55601945	0.31170812	0.68017626	1.02254428
ERBB2	-1.76969816	-0.54421283	0.30751732	0.68576547	1.02254428
TMEM125	-1.75178639	-0.74973614	0.42798377	0.59471232	1.02254428
FANK1	-1.73435173	-0.54152313	0.31223375	0.68704517	1.02254428
TIMM17A	-1.72142928	-0.39804183	0.23122752	0.75888762	1.02254428
PLA1A	-1.71516948	-0.75106635	0.43789629	0.59416423	1.02254428
AURKA	-1.71205998	-0.42019795	0.24543413	0.74732208	1.02254428
MTFR2	-1.69277288	-0.51354774	0.30337664	0.70049772	1.25432099
GATA3	-1.67568102	-0.74610251	0.4452533	0.59621207	1.25432099
WDR4	-1.67239986	-0.42201786	0.25234268	0.74637995	1.25432099
CD24	-1.66290608	-0.86453085	0.51989157	0.54922498	1.25432099
TP53	-1.66074511	-0.41543378	0.25014903	0.74979402	1.25432099
VAMP8	-1.59930618	-0.3636932	0.22740686	0.77717252	2.05787037
TFRC	-1.5940229	-0.44289687	0.2778485	0.73565596	2.05787037
KNTC2	-1.5885869	-0.52776793	0.33222478	0.69362705	2.05787037
TAP1	-1.5761967	-0.56071874	0.3557416	0.67796432	2.05787037
NLN	-1.5210268	-0.33610039	0.22096941	0.79217968	2.68783069
MCM3	-1.51733923	-0.34077814	0.22458929	0.78961531	2.68783069
IRX3	-1.49717169	-0.56121324	0.37484895	0.67773198	2.68783069
ERBB4	-1.48689826	-0.81518748	0.54824698	0.56833462	2.68783069
MYB	-1.48489355	-0.64836231	0.43663891	0.63800414	2.68783069
DEGS2	-1.48481833	-0.73972415	0.49819169	0.59885385	2.68783069
CDCA1	-1.48343743	-0.64822273	0.43697342	0.63806587	2.68783069
NEK2	-1.44660829	-0.6833559	0.47238489	0.62271507	2.68783069
RANBP1	-1.44468897	-0.26872316	0.18600762	0.83005385	2.68783069

HSPA14	-1.42324161	-0.31526754	0.22151372	0.80370193	3.32809224
PARP1	-1.41074761	-0.32205125	0.2282841	0.79993171	3.32809224
COX7B	-1.40038563	-0.29220398	0.20865965	0.81665352	3.32809224
LOC647456	-1.39921311	-0.61334301	0.43834853	0.65368024	3.32809224
FAM174B	-1.35247599	-0.44391514	0.32822404	0.73513691	4.16256965
RAD51C	-1.35155751	-0.28804942	0.21312406	0.81900864	4.16256965
PNP	-1.34319016	-0.34230672	0.25484606	0.78877913	4.16256965
CCNA2	-1.34314959	-0.44892426	0.33423251	0.7325889	4.16256965
MYO5C	-1.33830504	-0.45194783	0.33770166	0.73105516	4.16256965
DSP	-1.32774742	-0.69757845	0.5253849	0.61660631	4.16256965
EXO1	-1.32686561	-0.54461936	0.41045556	0.68557226	4.16256965
JUP	-1.31658842	-0.3971553	0.30165486	0.7593541	4.16256965
BRCA1	-1.29862558	-0.35825074	0.27586915	0.78010989	4.80169753
REEP6	-1.28993542	-0.6128723	0.47511859	0.65389355	4.80169753
ATAD2	-1.23044794	-0.37114488	0.30163395	0.77316869	6.45606391
STAT1	-1.2300721	-0.43979802	0.35753841	0.73723781	6.45606391
MRPS35	-1.22626967	-0.23629518	0.1926943	0.84892254	6.45606391
FOXA1	-1.19415785	-0.79589297	0.66648891	0.57598655	6.45606391
SLC39A6	-1.17870351	-0.43587715	0.36979371	0.73924416	6.45606391
IDO1	-1.17630622	-0.67773438	0.57615471	0.62514624	6.45606391
PUF60	-1.14936783	-0.27113532	0.23589952	0.82866717	6.45606391
CHEK2	-1.14882835	-0.26346848	0.22933668	0.83308264	6.45606391
CDC123	-1.10826677	-0.21432181	0.19338467	0.86195126	7.77989016
SEH1L	-1.07410889	-0.2219806	0.20666489	0.85738757	8.81944444
MRPS17	-1.07345194	-0.23895841	0.22260746	0.84735687	8.81944444
WDR12	-1.03486061	-0.20701971	0.20004598	0.86632502	8.81944444
HGH1	-1.02727539	-0.24429095	0.23780473	0.84423062	10.8927875
CCNE1	-1.02702617	-0.34634823	0.33723409	0.78657256	10.8927875
BYSL	-1.0099008	-0.23951548	0.23716733	0.84702974	10.8927875
AVL9	-1.00045555	-0.19759956	0.19750959	0.87200024	10.8927875
CCND1	-0.9913857	-0.34150213	0.34446949	0.78921915	10.8927875
PIEZO1	-0.98858843	-0.24124368	0.24402843	0.84601569	10.8927875
CDCA7	-0.96796519	-0.36327612	0.37529874	0.77739724	10.8927875
CDKN2A	-0.95714473	-0.34902632	0.36465366	0.78511379	10.8927875
TMEM25	-0.95242369	-0.32077428	0.33679788	0.80064007	10.8927875
MUC5B	-0.94974936	-0.6423552	0.6763418	0.64066621	10.8927875
RARA	-0.94941041	-0.26231051	0.2762878	0.83375158	10.8927875
AR	-0.9432722	-0.437973	0.46431243	0.73817102	10.8927875
GALNT7	-0.94243411	-0.28160363	0.29880458	0.82267606	10.8927875
CABP7	-0.92109414	-0.40218361	0.43663681	0.75671208	12.9544627
ASF1A	-0.91898997	-0.19726045	0.21464919	0.87220523	12.9544627
ATAD3A	-0.91572504	-0.20895326	0.22818341	0.86516472	12.9544627
ESR1	-0.91340054	-0.4801976	0.5257251	0.71687943	12.9544627
NUDT1	-0.90931795	-0.18254629	0.20075078	0.88114644	12.9544627
LRP8	-0.89763241	-0.31041265	0.34581266	0.80641107	12.9544627
SNRPD1	-0.89186444	-0.20400878	0.22874416	0.86813494	12.9544627
NQO1	-0.87323276	-0.32579021	0.37308519	0.79786125	12.9544627
TMEM208	-0.84296253	-0.15345662	0.18204442	0.8990937	15.2906777
CTPS1	-0.79776735	-0.2054871	0.25757773	0.86724583	16.9618406
GTPBP4	-0.78212772	-0.16854552	0.21549616	0.88973924	16.9618406

FBP1	-0.77146262	-0.33492605	0.43414423	0.79282478	16.9618406
PNO1	-0.74822418	-0.16428288	0.21956371	0.89237198	19.3480836
CELSR1	-0.73780333	-0.26478285	0.35887999	0.832324	19.3480836
TOR1A	-0.73088748	-0.13732039	0.18788172	0.90920631	19.3480836
S100A8	-0.71287313	-0.4595835	0.64469185	0.72719617	20.7360459
PDSS1	-0.67444478	-0.16126858	0.2391131	0.89423841	23.0674784
RBBP8	-0.6457185	-0.1875742	0.29048911	0.87808092	23.0674784
NFKBIE	-0.63425491	-0.1639941	0.25856181	0.89255062	23.0674784
RAF1	-0.631979	-0.11157013	0.17654088	0.92558018	23.0674784
TP53BP2	-0.6286153	-0.19168859	0.30493784	0.8755803	23.0674784
LAG3	-0.62509965	-0.24416386	0.39059989	0.84430499	23.0674784
CDK4	-0.61966811	-0.12262675	0.19789101	0.91851377	23.0674784
ATR	-0.61308241	-0.12154439	0.19825131	0.91920313	26.1978434
SNRPA1	-0.60552575	-0.12236312	0.20207748	0.91868163	26.1978434
DNAJC12	-0.59925828	-0.26466478	0.44165393	0.83239213	26.1978434
S100A14	-0.58694692	-0.3026742	0.51567559	0.81074819	26.1978434
NUP93	-0.58453043	-0.11591262	0.1983004	0.92279838	26.1978434
CTSV	-0.58144708	-0.19199065	0.33019455	0.875397	26.1978434
ARAF	-0.57514411	-0.1149584	0.19987755	0.92340894	26.1978434
CMC2	-0.56744678	-0.11596241	0.20435821	0.92276653	26.1978434
BOP1	-0.56291299	-0.18769535	0.33343582	0.87800718	26.1978434
INHBA	-0.56109454	-0.25185117	0.44885694	0.83981812	26.1978434
TFF1	-0.55312229	-0.41314482	0.74693215	0.75098458	26.1978434
NOP56	-0.5197749	-0.09863261	0.18976024	0.93391774	28.1046296
IGFBP2	-0.51927552	-0.22184448	0.42721922	0.85746847	28.1046296
MTOR	-0.51391283	-0.09446426	0.18381378	0.93662	28.1046296
S100A9	-0.50332532	-0.32070335	0.63716912	0.80067943	28.1046296
CDH3	-0.49168112	-0.2484559	0.50531917	0.8417969	28.1046296
CITED4	-0.48985302	-0.22151829	0.45221379	0.85766236	29.8810163
SLC25A19	-0.48580603	-0.1103532	0.22715486	0.92636124	29.8810163
CCDC86	-0.47933232	-0.11554858	0.24106152	0.92303127	29.8810163
SLC16A3	-0.47630076	-0.18656352	0.39169267	0.87869628	29.8810163
AREG	-0.46857558	-0.28121683	0.60015256	0.82289666	29.8810163
S100A11	-0.45007747	-0.11649199	0.25882653	0.92242787	29.8810163
PCDH8	-0.44144804	-0.20400108	0.46211799	0.86813958	29.8810163
USP10	-0.43442318	-0.08050985	0.18532587	0.94572337	29.8810163
LAMC2	-0.43146262	-0.21154904	0.49030677	0.86360947	29.8810163
MDM2	-0.4290149	-0.1116592	0.26026882	0.92552303	29.8810163
SLC52A2	-0.42132724	-0.10930658	0.25943392	0.92703353	32.2290809
TIMM8A	-0.42067255	-0.10816239	0.25711779	0.92776904	32.2290809
AKT1	-0.40581417	-0.0849857	0.20942025	0.94279388	32.2290809
RELB	-0.39717299	-0.12036934	0.30306528	0.91995211	32.2290809
CDKN1A	-0.39549056	-0.13891452	0.35124612	0.90820223	32.2290809
TM7SF3	-0.39152019	-0.09677944	0.24718888	0.93511815	32.2290809
CDYL	-0.38812257	-0.07645267	0.19698073	0.9483867	32.2290809
CYB5B	-0.38480561	-0.07570112	0.19672562	0.94888087	32.2290809
TUBA4A	-0.37192661	-0.11913986	0.32033164	0.92073643	32.2290809
BRCA2	-0.36785001	-0.10596324	0.288061	0.92918435	33.2910536
FGFR2	-0.36558615	-0.15928764	0.43570479	0.89546712	33.2910536
POLD1	-0.35389843	-0.08467505	0.23926371	0.94299691	33.2910536

RAB35	-0.32891804	-0.05674058	0.17250675	0.96143379	35.0399501
ATM	-0.32714724	-0.10951211	0.3347487	0.92690147	35.0399501
GPR89A	-0.32001107	-0.06491988	0.20286762	0.95599841	35.0399501
GABPB1	-0.31001204	-0.0551847	0.17800823	0.96247122	35.0399501
KRT6C	-0.30657335	-0.16822964	0.54874188	0.88993407	35.0399501
PREP	-0.27544295	-0.05677354	0.20611725	0.96141183	36.5958486
MUC1	-0.27284142	-0.13853574	0.50775187	0.90844071	36.5958486
AGR3	-0.2666961	-0.16504411	0.6188471	0.89190125	36.5958486
IL6	-0.26424915	-0.1359747	0.51457007	0.91005478	36.5958486
STRAP	-0.26208319	-0.05732659	0.21873433	0.96104335	36.5958486
MAP2K4	-0.26172995	-0.05207406	0.19896104	0.96454867	36.5958486
TCEAL1	-0.23853092	-0.06229194	0.26114826	0.9577414	38.3044226
EMC8	-0.23696843	-0.04843056	0.20437558	0.9669877	38.3044226
NR4A3	-0.22801267	-0.10184002	0.44664192	0.93184376	38.3044226
GPSM2	-0.22567945	-0.0700745	0.31050457	0.9525888	38.3044226
TMCC2	-0.22065901	-0.0788784	0.35746738	0.94679343	38.3044226
MIEN1	-0.19835049	-0.04177107	0.2105922	0.97146164	38.3044226
TMEM139	-0.19568625	-0.09963127	0.50913781	0.93327149	39.8750191
STK38L	-0.19485361	-0.05264825	0.27019388	0.96416486	39.8750191
P4HTM	-0.19233134	-0.05243108	0.27260811	0.96431	39.8750191
NUP88	-0.18538409	-0.03530236	0.19042822	0.97582722	39.8750191
UIMC1	-0.18133317	-0.03729659	0.20567988	0.97447928	39.8750191
IKBKE	-0.17591817	-0.05048393	0.28697393	0.96561238	39.8750191
NCAPH2	-0.17524219	-0.03607412	0.20585296	0.97530535	39.8750191
TRIM29	-0.15387096	-0.09518362	0.61859383	0.93615309	40.583785
ASUN	-0.1469648	-0.03308085	0.22509368	0.977331	40.583785
KRT23	-0.14559376	-0.09608322	0.65994052	0.93556953	40.583785
YBX1	-0.14485522	-0.0353108	0.24376615	0.97582152	40.583785
PROM1	-0.13686963	-0.09194944	0.67180304	0.93825408	40.583785
XBP1	-0.11961894	-0.04623459	0.38651566	0.9684607	41.8212366
CDKN2D	-0.10743534	-0.03247898	0.30231191	0.9777388	41.8212366
GARS	-0.10724781	-0.02375	0.22144969	0.98367252	41.8212366
ECE2	-0.09930597	-0.03677538	0.370324	0.97483139	41.8212366
NAT1	-0.09762279	-0.0391942	0.40148618	0.97319836	41.8212366
BCL2A1	-0.09526561	-0.04191935	0.4400261	0.97136179	41.8212366
CXXC5	-0.08033225	-0.02183615	0.27182296	0.9849783	42.8948322
EPSTI1	-0.07989126	-0.03015725	0.37747866	0.97931355	42.8948322
BTG2	-0.07906996	-0.03110209	0.39334899	0.9786724	42.8948322
TFAM	-0.07255759	-0.01533746	0.21138321	0.9894252	42.8948322
DDIT4	-0.0689052	-0.02320806	0.33681138	0.9840421	42.8948322
TMEM45B	-0.06526706	-0.02691779	0.41242538	0.98151499	42.8948322
C8orf33	-0.04706114	-0.01155381	0.24550639	0.99202349	42.8948322
MKRN2	-0.03352429	-0.007018	0.20934069	0.99514731	42.8948322
IFT74	-0.02913653	-0.00692829	0.23778707	0.99520919	42.8948322
NPM2	-0.02211111	-0.00837843	0.378924	0.99420935	42.8948322
CCND3	-0.00019138	-0.0000473	0.24703104	0.99996723	42.8948322

SupplementaryTable 4. Gene expression profiles between Baseline and surgical samples. Lists of differentially expressed genes between baseline and surgical tumors determined by SAM analysis.

Gene ID	Score(d)	Numerator(r)	Denominator(	Fold Change	q-value(%)
CYR61	13.6143349	2.06073699	0.15136523	4.17199374	0
NR4A3	10.87957	1.8494174	0.16998994	3.60354634	0
PTGS2	9.84484826	1.40943555	0.14316478	2.65633214	0
CTGF	9.05179847	1.08960897	0.12037486	2.12816347	0
IL6	8.68046623	1.45140324	0.16720337	2.73473917	0
KLF4	8.11128765	1.00522025	0.12392857	2.00724992	0
BTG2	7.81125142	1.0060117	0.12879008	2.00835137	0
CXCL1	7.21751416	1.1182153	0.15493081	2.17078267	0
CDKN1A	5.4706037	0.47360435	0.08657259	1.38857428	0
OGN	5.29031273	0.97066898	0.18348045	1.95974912	0
SFRP1	5.16804173	0.87480327	0.16927171	1.83375802	0
ADM	5.05864575	0.70606588	0.13957607	1.63134948	0
FABP4	5.0405606	1.17932705	0.23396744	2.26471114	0
MYC	5.00583348	0.48861019	0.09760816	1.40309256	0
TWIST1	4.92988162	0.59913302	0.12153091	1.51480598	0
IGF1	4.7896174	0.65016887	0.13574547	1.56935188	0
NFKBIA	4.69947024	0.34755866	0.07395699	1.27240563	0
TGFBR3	4.47539519	0.55038443	0.12298007	1.46447588	0
CXCL8	4.28003545	0.74681876	0.17448892	1.67808845	0
ID4	4.21470861	0.52433468	0.12440592	1.43827015	0
FOXC1	4.18491884	0.4292469	0.10256995	1.3465305	0
NTN4	4.0461639	0.42038526	0.10389724	1.33828488	0
F3	3.93034462	0.40904222	0.10407286	1.32780401	0
KRT17	3.74716766	0.76045096	0.2029402	1.69402006	0
ANXA1	3.74114455	0.33631666	0.08989673	1.26252912	0
KRT14	3.71731531	0.8580414	0.23082287	1.81257589	0
CAPN6	3.65597461	0.64767665	0.17715568	1.56664321	0
STC2	3.65153255	0.60964391	0.16695563	1.52588254	0
BTG3	3.59365377	0.25313354	0.07043905	1.19179289	0
DDIT4	3.3923497	0.33928576	0.10001497	1.26513011	0
ABCB1	3.33285802	0.41904859	0.12573251	1.33704553	0
CDKN1B	3.29231256	0.25853812	0.07852782	1.19626592	0
FIGF	3.20298308	0.514638	0.16067459	1.42863562	0
PIK3CA	3.20077054	0.17175877	0.05366169	1.12643086	0
TWIST2	3.18731473	0.37003507	0.11609618	1.29238425	0
ANGPTL4	3.15965584	0.4744312	0.15015281	1.38937033	0
KRAS	3.13061815	0.21126002	0.06748189	1.15769885	0
KIT	3.12496168	0.3830107	0.12256493	1.3040604	0
KRT5	3.09144479	0.57656081	0.18650206	1.49128997	0
NDRG1	3.04461803	0.30276203	0.09944171	1.23350369	0
MET	2.96551089	0.33914892	0.11436441	1.26501011	0
CYBRD1	2.95705744	0.28714368	0.09710453	1.22022203	0
NFIB	2.94515004	0.29060223	0.09867145	1.22315076	0
RANBP1	2.86966245	0.14232974	0.04959808	1.10368597	0
TSHZ1	2.84838441	0.19515471	0.06851418	1.14484692	0
OGFRL1	2.81129705	0.21294277	0.07574538	1.15904997	0

GNG11	2.79983331	0.25797515	0.09213947	1.1957992	0
IRX3	2.78066326	0.25792094	0.09275519	1.19575427	0
EGFR	2.77300377	0.29545772	0.1065479	1.22727429	0
STRAP	2.74216119	0.16864181	0.0614996	1.12399982	0
CXCL14	2.68411129	0.41913127	0.15615272	1.33712216	0
KRT23	2.6762587	0.40054423	0.14966574	1.32000576	0
NFIA	2.67437025	0.22053792	0.08246349	1.16516794	0
CITED4	2.67182551	0.29716863	0.11122307	1.2287306	0
CRYAB	2.6523064	0.36768059	0.13862674	1.29027679	0
ESR1	2.61211255	0.35532912	0.13603132	1.27927739	0.3433642
APH1B	2.60697095	0.19160722	0.07349803	1.14203528	0.3433642
AHCYL1	2.58605232	0.14323462	0.05538737	1.10437844	0.3433642
KRT6B	2.57318396	0.46512038	0.18075675	1.38043253	0.3433642
PIP	2.56218163	0.63236518	0.24680732	1.55010418	0.3433642
LHFP	2.54115516	0.24599747	0.09680537	1.18591242	0.3433642
RAI2	2.51228826	0.2628607	0.10462999	1.19985552	0.3433642
TM7SF3	2.50796423	0.15519412	0.06188052	1.11357144	0.3433642
FZD6	2.48453655	0.17510684	0.07047868	1.12904802	0.3433642
COX6C	2.43585369	0.25629639	0.1052183	1.19440854	0.3433642
CDKN2C	2.42697083	0.19838763	0.0817429	1.14741527	0.3433642
BCL2	2.42094157	0.21057375	0.0869801	1.15714828	0.3433642
TP63	2.38735231	0.36637501	0.15346499	1.28910967	0.77742837
HIF1A	2.36777047	0.16594863	0.07008645	1.12190353	0.77742837
CAV1	2.3470578	0.25647387	0.10927463	1.19455549	0.77742837
ITGA6	2.33813116	0.19782325	0.08460742	1.1469665	0.77742837
PREP	2.23821936	0.1254254	0.05603803	1.09082933	0.77742837
AREG	2.21217773	0.37009693	0.16729982	1.29243966	0.77742837
CLDN4	2.16366143	0.24710219	0.11420557	1.18682086	1.40867363
PTGER4	2.1463005	0.21352379	0.09948457	1.15951686	1.40867363
IL6ST	2.12097176	0.1662606	0.07838888	1.12214616	1.40867363
SEMA3C	2.09678649	0.18496265	0.08821244	1.13678755	1.40867363
RERG	2.07402344	0.20888664	0.10071566	1.15579588	1.40867363
CRIM1	2.0705878	0.16341288	0.07892101	1.11993336	1.40867363
ALDH1A1	2.05718713	0.26304692	0.12786728	1.20001041	1.40867363
TRIM29	2.01836129	0.28563788	0.1415197	1.2189491	1.40867363
CYB5B	1.96098635	0.09397669	0.04792318	1.0673081	2.57523148
THBS1	1.86137523	0.1592091	0.08553305	1.1166748	2.57523148
PROM1	1.85321131	0.28760406	0.15519227	1.22061148	2.57523148
SMIM14	1.83951977	0.12882441	0.07003155	1.09340237	2.57523148
PID1	1.7925121	0.19972237	0.11142037	1.14847732	4.62430056
VIM	1.78776504	0.1431391	0.08006595	1.10430531	4.62430056
FZD7	1.76519852	0.1563624	0.08858063	1.11447357	4.62430056
STK38L	1.73081799	0.11057496	0.06388595	1.07965843	4.62430056
TIMM17A	1.71992712	0.0997722	0.05800955	1.07160424	4.62430056
PIK3R1	1.71294587	0.13339699	0.07787577	1.09687337	4.62430056
YBX3	1.68678901	0.13573047	0.08046678	1.09864895	4.62430056
ERBB4	1.67550209	0.19860651	0.11853552	1.14758937	4.62430056
INSIG1	1.66598698	0.13456698	0.08077313	1.09776327	4.62430056
BRAF	1.65784562	0.09374127	0.05654403	1.06713394	4.62430056
PUM1	1.62025484	0.07226697	0.04460222	1.05136744	4.62430056

FAM198B	1.6012933	0.14546268	0.09084075	1.10608533	4.62430056
DSP	1.59661815	0.18262596	0.11438299	1.13494781	7.12291156
ADRA2A	1.59111998	0.17019572	0.10696598	1.12521112	7.12291156
TFAM	1.58827226	0.09100798	0.05729999	1.0651141	7.12291156
MAP2K1	1.58403917	0.07179044	0.04532113	1.05102023	7.12291156
SPATA7	1.57635569	0.10216439	0.06481049	1.07338259	7.12291156
FOXA1	1.57527943	0.21298004	0.13520144	1.15907992	7.12291156
IL1B	1.53675335	0.18951296	0.12332035	1.14037867	7.12291156
PARP1	1.51775129	0.08665453	0.05709403	1.06190487	7.12291156
CDCA7	1.49047041	0.14924105	0.10013017	1.10898592	7.12291156
H19	1.48687576	0.18416245	0.12385866	1.13615719	7.12291156
TCF7L1	1.44834929	0.13174698	0.09096354	1.0956196	7.12291156
NOTCH2	1.41291474	0.08153938	0.05771005	1.0581465	7.12291156
EMC8	1.39713792	0.07412271	0.05305325	1.05272069	10.1783653
RECK	1.3862151	0.13079121	0.09435131	1.094894	10.1783653
HSPD1	1.37899875	0.08893619	0.06449331	1.06358563	10.1783653
SLC39A6	1.37124117	0.15335307	0.11183523	1.1121513	10.1783653
PSMD14	1.36391429	0.07373236	0.05405938	1.0524359	10.1783653
DDB2	1.34518914	0.09449371	0.07024567	1.06769066	10.1783653
C8orf33	1.337097	0.09361388	0.07001278	1.06703972	10.1783653
CTNNB1	1.31387968	0.07913214	0.06022785	1.05638238	10.1783653
MAP7D3	1.31020312	0.11036251	0.08423313	1.07949945	10.1783653
CD44	1.27980598	0.09303325	0.07269325	1.06661037	10.1783653
ZEB1	1.24547219	0.10432159	0.08376068	1.07498877	10.1783653
PDGFRA	1.23281751	0.11959784	0.0970118	1.08643197	13.1939519
PYROXD1	1.19355638	0.06557923	0.0549444	1.04650502	13.1939519
GSTP1	1.18819543	0.09128269	0.07682464	1.06531693	13.1939519
WIPF2	1.18593011	0.06016459	0.05073198	1.04258469	13.1939519
NEO1	1.16986529	0.0819705	0.07006832	1.05846275	13.1939519
CDYL	1.16093982	0.06140632	0.05289363	1.04348244	13.1939519
RAF1	1.15770884	0.05392859	0.04658217	1.03808789	13.1939519
CLMN	1.11224765	0.10073541	0.09056923	1.07231994	13.1939519
LAMA3	1.09702352	0.12649743	0.11530968	1.0916402	16.5221765
PNO1	1.09294167	0.060656	0.05549793	1.04293988	16.5221765
MME	1.08701045	0.15817448	0.1455133	1.11587427	16.5221765
SERPINA3	1.08129728	0.1788532	0.16540613	1.13198372	16.5221765
COX7B	1.05262179	0.05400481	0.05130505	1.03814273	16.5221765
AKT3	1.05180594	0.08893789	0.08455731	1.06358688	16.5221765
SCGB2A2	1.04482698	0.26215653	0.25090904	1.19927002	16.5221765
FGFR1	1.04336706	0.08807151	0.08441086	1.06294836	16.5221765
MIA	1.03990733	0.13896072	0.13362798	1.10111162	16.5221765
MKRN2	1.03926762	0.06233803	0.05998265	1.04415655	16.5221765
AFF3	0.98998419	0.11530752	0.1164741	1.08320591	16.5221765
GALNT7	0.98915009	0.07768478	0.0785369	1.05532311	16.5221765
MAP2K4	0.98809415	0.06477734	0.06555786	1.0459235	16.5221765
PTEN	0.97786376	0.05356662	0.05477923	1.03782746	16.5221765
IGBP1	0.97046638	0.05519366	0.05687334	1.03899857	16.5221765
VEGFA	0.96879048	0.09761733	0.10076206	1.07000484	16.5221765
NT5E	0.96699346	0.1000503	0.10346533	1.07181083	16.5221765
CA12	0.94337437	0.1112289	0.11790537	1.08014793	16.5221765

ELOVL5	0.92003819	0.08517194	0.09257436	1.06081416	19.8526936
TP53BP2	0.91887988	0.05496599	0.05981847	1.03883461	19.8526936
AGR3	0.91379254	0.1489819	0.1630369	1.10878673	19.8526936
TGFBR2	0.91378774	0.08308145	0.09091985	1.05927813	19.8526936
GAL	0.90076342	0.11720336	0.13011559	1.08463029	19.8526936
MYB	0.90032597	0.09221173	0.10242038	1.06600317	19.8526936
PRAME	0.89595385	0.17116363	0.19104068	1.12596629	19.8526936
RELA	0.89233997	0.04467638	0.05006655	1.03145178	19.8526936
LRIG1	0.86363429	0.05872096	0.06799285	1.04154196	19.8526936
GSTM3	0.862185	0.07660927	0.08885479	1.05453668	19.8526936
YBX1	0.85884123	0.05512934	0.06419037	1.03895224	19.8526936
FABP5	0.83791579	0.08715239	0.10401092	1.06227139	19.8526936
GARS	0.83748638	0.04142039	0.04945799	1.02912655	19.8526936
KIF13B	0.83685883	0.06425295	0.07677872	1.0455434	19.8526936
KLHL7	0.81623377	0.04898248	0.06001036	1.03453502	19.8526936
MRPS35	0.81059698	0.04078282	0.05031207	1.02867184	19.8526936
RNF103	0.8101228	0.0482807	0.05959677	1.03403191	19.8526936
CHUK	0.805746	0.03960038	0.04914747	1.02782908	19.8526936
TCEAL1	0.80269645	0.0660827	0.08232589	1.04687029	19.8526936
PSMA7	0.77996242	0.04274773	0.05480742	1.03007382	19.8526936
ME1	0.76597501	0.08953803	0.11689419	1.06402941	19.8526936
TUBA4A	0.74360519	0.05759004	0.07744707	1.04072582	20.6754299
TSPAN13	0.74232025	0.07086874	0.09546922	1.05034897	20.6754299
KIAA1324	0.73909842	0.08658405	0.1171482	1.06185299	20.6754299
ANXA8L2	0.70780627	0.12708348	0.17954557	1.09208374	20.6754299
AZGP1	0.70361972	0.09465224	0.13452187	1.06780799	20.6754299
SCUBE2	0.70318347	0.08894336	0.12648671	1.06359092	20.6754299
GLRB	0.66967677	0.06371044	0.09513611	1.04515031	20.6754299
XBP1	0.66812438	0.06294387	0.0942098	1.04459512	20.6754299
F11R	0.66168	0.04631862	0.07000154	1.03262657	20.6754299
AGR2	0.65443615	0.09755393	0.14906562	1.06995782	20.6754299
CDKN2D	0.61925523	0.05546708	0.08957062	1.03919549	22.2357918
GOLT1A	0.61542608	0.06509291	0.10576886	1.04615231	22.2357918
STAT3	0.61279618	0.0300203	0.04898905	1.02102649	22.2357918
MUC1	0.59826897	0.08006388	0.13382589	1.05706484	22.2357918
KPNA1	0.58362692	0.02517085	0.04312832	1.01760019	22.2357918
VAMP8	0.5795321	0.03281687	0.05662649	1.02300761	22.2357918
CDH1	0.57884121	0.05810284	0.10037786	1.04109581	22.2357918
FBP1	0.5759903	0.06005231	0.10425923	1.04250356	22.2357918
PEX11G	0.57416743	0.04033137	0.07024322	1.02835	22.2357918
GPR160	0.57374772	0.05499002	0.09584355	1.03885191	22.2357918
CDKN2A	0.56789203	0.05480069	0.09649844	1.0387156	22.2357918
IGFBP2	0.54716612	0.0553566	0.10116964	1.03911592	22.2357918
KCTD1	0.5085667	0.0435571	0.08564678	1.03065186	22.2357918
GRHL1	0.49922989	0.04672861	0.09360138	1.03292006	22.2357918
SLC40A1	0.49876691	0.05551012	0.11129471	1.0392265	22.2357918
RBBP8	0.48587639	0.03938184	0.08105321	1.0276734	23.4651601
FNBP1	0.476041	0.02739	0.05753706	1.01916667	23.4651601
RAD50	0.46679367	0.02914304	0.06243238	1.02040582	23.4651601
RRAGD	0.46246339	0.04587	0.09918622	1.03230551	23.4651601

CXCR1	0.4489044	0.04867206	0.10842411	1.03431244	23.4651601
BMI1	0.37550352	0.02735309	0.07284377	1.01914059	24.5505401
ZNF217	0.37236885	0.02314722	0.06216208	1.01617384	24.5505401
CKS1B	0.37167712	0.02451151	0.06594839	1.01713523	24.5505401
CD24	0.36816831	0.04616695	0.12539633	1.03251802	24.5505401
PSPHL	0.36488905	0.04779136	0.13097504	1.03368124	24.5505401
CAND1	0.35579598	0.0168864	0.0474609	1.01177353	24.5505401
TOM1L1	0.34856703	0.02885992	0.08279591	1.0202056	24.5505401
PGR	0.34825119	0.05383132	0.15457612	1.0380179	24.5505401
CXXC5	0.30036693	0.02053013	0.06835018	1.01433214	25.7205871
JUP	0.29453259	0.01956464	0.06642607	1.01365355	25.7205871
NQO1	0.29444493	0.02966408	0.10074575	1.02077442	25.7205871
C4orf32	0.29077441	0.02399961	0.08253688	1.0167744	25.7205871
ECE2	0.27477908	0.02999638	0.10916546	1.02100957	25.7205871
CEACAM6	0.26239236	0.04722551	0.17998049	1.03327589	25.7205871
TMEM139	0.25931216	0.03370806	0.12999029	1.02363974	25.7205871
CCND3	0.21148815	0.01350396	0.06385209	1.00940418	26.4784457
C16orf45	0.19170186	0.01785191	0.09312331	1.01245088	26.4784457
NCAPH2	0.18338496	0.01025807	0.05593735	1.00713569	26.4784457
RAB25	0.17367187	0.02038623	0.11738359	1.01423097	26.4784457
GRB7	0.15768796	0.01389381	0.08810949	1.00967697	26.4784457
DNALI1	0.15694683	0.01490156	0.09494656	1.0103825	26.4784457
BCL2A1	0.14970005	0.01707118	0.11403592	1.01190313	26.4784457
ADRA2C	0.13097407	0.01615212	0.12332301	1.0112587	26.4784457
IDH2	0.12662369	0.00890931	0.07036056	1.00619457	26.4784457
DNAJC12	0.12162365	0.01560218	0.12828246	1.0108733	26.4784457
CDC123	0.11920622	0.00545384	0.04575132	1.00378747	26.4784457
FBN1	0.11211432	0.01054856	0.09408755	1.0073385	26.4784457
NRAS	0.10619084	0.00589296	0.05549401	1.00409304	26.4784457
CYCS	0.08069363	0.00663138	0.08217972	1.0046071	26.4784457
INHBA	0.08017929	0.00867831	0.10823629	1.00603347	26.4784457
SETBP1	0.07801917	0.00651595	0.08351734	1.00452673	26.4784457
SPINT1	0.07651053	0.00573827	0.07499979	1.00398539	26.4784457
PHGDH	0.06598396	0.00647819	0.09817831	1.00450044	26.4784457
CBX7	0.06594128	0.00579815	0.087929	1.00402706	26.4784457
RECQL	0.03818005	0.0021291	0.0557648	1.00147687	26.4784457
CLDN3	0.00629447	0.0007439	0.118183	1.00051576	26.4784457
PSPH	-6.77525798	-0.45398101	0.06700572	0.73002562	0
BCL11A	-6.27119038	-0.85100567	0.13570082	0.55439814	0
HGH1	-5.87706319	-0.35361737	0.06016906	0.78261932	0
CCDC86	-5.8293401	-0.356982	0.06123883	0.78079624	0
TIMM8A	-5.81761791	-0.43952401	0.07555051	0.73737785	0
MDM2	-5.44529873	-0.41126946	0.07552744	0.75196142	0
EVI2A	-5.43302994	-0.57868756	0.10651286	0.66957262	0
PIEZO1	-5.32371732	-0.3099946	0.05822898	0.80664478	0
SPAG5	-4.98715842	-0.45065367	0.09036281	0.73171124	0
NOP56	-4.9093172	-0.25540732	0.05202502	0.83775058	0
SLC52A2	-4.79838329	-0.31755037	0.06617862	0.80243121	0
SUV39H2	-4.75212604	-0.31771005	0.0668564	0.8023424	0
CDCA7L	-4.41705522	-0.44832634	0.10149892	0.73289258	0

SRC	-4.32600543	-0.28019987	0.06477104	0.82347693	0
ATAD3A	-4.32592776	-0.2612914	0.06040124	0.83434074	0
KIF23	-4.3039515	-0.41458614	0.09632686	0.75023468	0
AURKA	-4.27088758	-0.35156651	0.08231697	0.78373264	0
TACC3	-4.26111338	-0.39081835	0.09171743	0.76269685	0
POLD1	-4.23038819	-0.27850819	0.06583514	0.82444309	0
KNTC2	-4.14203478	-0.43920854	0.1060369	0.73753911	0
HJURP	-4.12889179	-0.46619115	0.11290951	0.72387317	0
CCNE1	-4.10645425	-0.37567634	0.09148436	0.770744	0
CENPN	-4.0160902	-0.31520654	0.07848592	0.80373592	0
SLC25A19	-4.0106696	-0.26634009	0.06640788	0.83142608	0
CTPS1	-3.99441472	-0.25475301	0.06377731	0.83813061	0
MELK	-3.92806645	-0.41106492	0.10464816	0.75206803	0
NUP93	-3.90682454	-0.22293523	0.05706302	0.85682042	0
CHPF	-3.88759606	-0.3091523	0.07952274	0.80711586	0
ABCC3	-3.86060219	-0.35780561	0.09268129	0.78035062	0
ANLN	-3.85246287	-0.43799787	0.11369295	0.73815829	0
BAG1	-3.79623082	-0.28838395	0.07596586	0.81881875	0
CDT1	-3.74086537	-0.3989433	0.10664466	0.75841358	0
CHEK1	-3.73420414	-0.33228831	0.08898504	0.79427566	0
BRCA2	-3.72407355	-0.31138872	0.08361508	0.80586567	0
BRCA1	-3.65084235	-0.28559161	0.07822622	0.82040512	0
IFT74	-3.6495435	-0.23882675	0.06544017	0.8474342	0
RAD51	-3.6468054	-0.33199858	0.0910382	0.79443518	0
LRP8	-3.62601763	-0.31410393	0.08662504	0.80435042	0
KIF2C	-3.62255538	-0.37508635	0.10354192	0.77105926	0
FBXL6	-3.62128705	-0.26189328	0.0723205	0.83399273	0
CENPI	-3.59997754	-0.34602891	0.09611974	0.78674668	0
TK1	-3.59898063	-0.35196961	0.09779703	0.78351369	0
CD68	-3.52359951	-0.40351785	0.11451865	0.75601258	0
CCNA2	-3.49931728	-0.33931406	0.09696579	0.79041703	0
FLVCR2	-3.48152115	-0.33442787	0.09605797	0.7930986	0
RAD17	-3.47319643	-0.21979025	0.06328184	0.85869027	0
KIFC1	-3.47059561	-0.3811773	0.10983051	0.76781077	0
FANCA	-3.46132808	-0.33212712	0.09595367	0.7943644	0
MTFR2	-3.45332091	-0.31035965	0.08987281	0.8064407	0
TTK	-3.42400875	-0.31553927	0.09215493	0.80355057	0
CDCA8	-3.40354885	-0.34521338	0.10142748	0.78719154	0
MYBL2	-3.38474832	-0.44024567	0.13006747	0.7370091	0
NDUFAF4	-3.37149447	-0.25961592	0.07700322	0.83531027	0
THY1	-3.3515001	-0.29794712	0.08889963	0.81340901	0
EXO1	-3.34488318	-0.37001342	0.11062073	0.7737753	0
WDR12	-3.30612285	-0.20357348	0.06157469	0.86839692	0
MKI67	-3.30006454	-0.38135216	0.115559	0.76771771	0
FOXM1	-3.29310869	-0.41305919	0.12543139	0.75102915	0
E2F1	-3.26445817	-0.34227108	0.10484774	0.78879861	0
RRM2	-3.22745021	-0.40150946	0.12440454	0.75706577	0
CHEK2	-3.21440329	-0.19519591	0.0607254	0.87345428	0
COG8	-3.20478262	-0.1837471	0.05733528	0.88041333	0
KIF4A	-3.20000549	-0.34078468	0.10649503	0.78961173	0

CDC20	-3.17152267	-0.32962285	0.10393205	0.79574448	0
TMEM125	-3.16628963	-0.31082552	0.09816712	0.80618033	0
NOTCH1	-3.15920178	-0.22355785	0.07076403	0.85645073	0
SLC5A6	-3.15837388	-0.2420593	0.07664048	0.84553753	0
CEP55	-3.13816817	-0.32683678	0.10414891	0.79728267	0
WDR4	-3.1263998	-0.19318422	0.06179127	0.87467307	0
TOMM40	-3.12057063	-0.17865817	0.05725176	0.88352437	0
CENPA	-3.10896054	-0.3572526	0.11491062	0.7806498	0
IKBKE	-3.06165585	-0.23251615	0.07594457	0.85114914	0
ATAD2	-3.03290816	-0.25496897	0.08406749	0.83800516	0
GINS2	-3.00931899	-0.29399948	0.09769635	0.81563778	0
LTBP2	-2.93715346	-0.25418402	0.08654094	0.83846123	0
ACOT4	-2.93324314	-0.22644895	0.07720088	0.85473615	0
CDCA5	-2.92605468	-0.33509277	0.11452034	0.79273316	0
PCNA	-2.92597582	-0.20588877	0.07036585	0.86700441	0
ORC6L	-2.89563558	-0.29863825	0.10313392	0.81301944	0
CDC25C	-2.88672849	-0.31233737	0.10819769	0.80533594	0
ATR	-2.88611634	-0.15806724	0.05476814	0.89622493	0
CD86	-2.88559267	-0.27673489	0.09590227	0.82545708	0
ZEB2	-2.88557809	-0.31162677	0.10799457	0.80573271	0
FAM214A	-2.87866998	-0.22466761	0.07804563	0.85579217	0
BUB1	-2.87207841	-0.29031178	0.10108073	0.81772532	0
P3H1	-2.86128775	-0.21314808	0.07449376	0.8626528	0
BOP1	-2.85104656	-0.22052691	0.07734946	0.85825193	0
CLDN7	-2.84643103	-0.28746455	0.10099122	0.81934074	0
STAT1	-2.84133154	-0.2735385	0.09627123	0.82728797	0
SH2B3	-2.84052841	-0.22451114	0.07903851	0.855885	0
MTOR	-2.8319992	-0.14114535	0.04983948	0.90679896	0
RAD51B	-2.83075278	-0.19105618	0.06749306	0.8759642	0
HRAS	-2.75201262	-0.16447985	0.05976711	0.89225015	0
S100A14	-2.74832747	-0.34094012	0.12405367	0.78952666	0
DLGAP5	-2.73672843	-0.3129025	0.11433451	0.80502054	0
MMP11	-2.73237904	-0.42012294	0.1537572	0.74736093	0
EZH2	-2.71883706	-0.21826122	0.08027742	0.85960083	0
CDC45	-2.65303994	-0.29810608	0.11236396	0.81331939	0
BIRC5	-2.59049278	-0.32972499	0.12728273	0.79568815	0
PGAM5	-2.59033777	-0.1360886	0.05253701	0.90998294	0
BLM	-2.58173976	-0.22715	0.08798331	0.85432091	0
PDXK	-2.57482468	-0.1402331	0.05446316	0.90737254	0
CDK1	-2.56955322	-0.27812852	0.10824003	0.82466008	0
KDR	-2.56616829	-0.20229209	0.0788304	0.86916857	0
BYSL	-2.54402564	-0.14795234	0.05815678	0.90253054	0
SLC7A6	-2.53159836	-0.18193629	0.07186617	0.88151909	0
MAD2L1	-2.51171708	-0.19854136	0.07904607	0.87143118	0
CDKN3	-2.49196054	-0.24000566	0.09631198	0.84674199	0
RINT1	-2.47585491	-0.15056819	0.06081463	0.90089559	0
EIF2S2	-2.45700175	-0.26444954	0.107631	0.83251632	0
MLKL	-2.43283657	-0.20652756	0.08489167	0.8666206	0
NACC2	-2.38401625	-0.14396749	0.06038863	0.90502686	0
NAT1	-2.33664385	-0.28383485	0.12147116	0.82140473	0

HN1	-2.30020159	-0.1867085	0.08117049	0.87860798	0
CENPF	-2.27898388	-0.26601501	0.11672527	0.83161345	0.3433642
CDC6	-2.24389205	-0.21949087	0.09781703	0.85886848	0.3433642
GPR89A	-2.2388861	-0.12674831	0.05661222	0.91589346	0.3433642
PTDSS1	-2.22925883	-0.13770945	0.06177365	0.90896116	0.3433642
CCNB1	-2.22757683	-0.20584397	0.09240712	0.86703133	0.3433642
PDSS1	-2.22360901	-0.14137138	0.06357745	0.9066569	0.3433642
UBE2C	-2.22289685	-0.20873366	0.09390164	0.86529642	0.3433642
CHST11	-2.22112674	-0.16945745	0.07629346	0.88917701	0.3433642
KDM4B	-2.21078903	-0.16686584	0.07547796	0.89077573	0.3433642
MTHFD1L	-2.19306937	-0.15906319	0.07252994	0.89560644	0.3433642
NUDT1	-2.18895726	-0.11555994	0.05279223	0.92302399	0.3433642
CDCA1	-2.17142454	-0.24123959	0.11109738	0.84601809	0.3433642
MCM2	-2.16623674	-0.18255973	0.08427506	0.88113823	0.3433642
C1orf21	-2.15542369	-0.22030661	0.10221035	0.85838299	0.3433642
SLC16A3	-2.11252964	-0.20427865	0.09669859	0.86797257	0.3433642
IGF2R	-2.10864785	-0.12856115	0.06096852	0.9147433	0.3433642
CTSV	-2.07932306	-0.2205411	0.1060639	0.85824348	0.3433642
TMEM208	-2.05975438	-0.09776498	0.04746439	0.93447956	0.3433642
APC	-2.04218894	-0.11008555	0.05390566	0.92653312	0.3433642
PNP	-2.02535324	-0.12859411	0.06349219	0.9147224	0.77742837
PRC1	-2.01135737	-0.21774699	0.10825873	0.85990728	0.77742837
NFKBIE	-1.99917183	-0.13015076	0.06510234	0.91373596	0.77742837
CTSL	-1.96724597	-0.18532835	0.094207	0.8794489	0.77742837
AR	-1.9516158	-0.19069328	0.09771046	0.87618457	0.77742837
TAP1	-1.93635903	-0.18496749	0.09552334	0.8796689	0.77742837
TMEM45B	-1.9249415	-0.22294272	0.11581792	0.85681597	0.77742837
MLPH	-1.91907865	-0.19422641	0.10120816	0.87404144	0.77742837
TYMS	-1.86817833	-0.1850651	0.09906179	0.87960939	0.77742837
LOC642077	-1.8267543	-0.17716419	0.09698304	0.88443977	0.77742837
STMN1	-1.82190147	-0.18914161	0.1038155	0.87712745	0.77742837
MCM3	-1.82157687	-0.10622069	0.05831249	0.92901855	0.77742837
CDC25B	-1.80451322	-0.12684978	0.07029585	0.91582904	1.40867363
UBE2T	-1.80447845	-0.18407143	0.1020081	0.88021543	1.40867363
ABAT	-1.76965719	-0.16285255	0.09202491	0.89325715	1.40867363
LOC647867	-1.76555705	-0.20558372	0.11644128	0.86718775	1.40867363
AARS	-1.75360139	-0.09203218	0.05248181	0.93820027	1.40867363
RGS22	-1.75071447	-0.22291434	0.12732764	0.85683282	1.40867363
PPFIBP1	-1.74156393	-0.1008047	0.05788171	0.93251272	1.40867363
RACGAP1	-1.7293861	-0.1680756	0.09718801	0.89002909	1.40867363
GSTM4	-1.71600464	-0.11266884	0.06565766	0.92487555	1.40867363
PTTG1	-1.69081382	-0.15107589	0.08935099	0.90057861	1.40867363
MGC18216	-1.68190329	-0.16986349	0.1009948	0.88892679	1.40867363
IKBKB	-1.67952357	-0.11240066	0.06692414	0.92504749	1.40867363
ERBB2	-1.66468374	-0.13516188	0.08119373	0.91056766	1.40867363
GTPBP4	-1.59498448	-0.08863475	0.05557092	0.94041226	1.40867363
RARA	-1.58955229	-0.11117647	0.069942	0.92583277	2.57523148
HSPA14	-1.58548162	-0.08492135	0.05356186	0.94283594	2.57523148
KLHL9	-1.57880369	-0.09401625	0.05954904	0.9369109	2.57523148
TP53	-1.57775443	-0.08767337	0.05556845	0.94103913	2.57523148

A1CF	-1.57346968	-0.19996258	0.12708384	0.87057315	2.57523148
RHBG	-1.5645825	-0.16419069	0.10494217	0.89242901	2.57523148
PUF60	-1.56441388	-0.09231395	0.05900865	0.93801705	2.57523148
MAGEA1	-1.55355883	-0.20264997	0.13044242	0.86895299	2.57523148
EPSTI1	-1.54235305	-0.1529605	0.09917347	0.89940294	2.57523148
MAGOHB	-1.53442233	-0.10847861	0.07069671	0.92756571	2.57523148
PLOD1	-1.51008418	-0.11200339	0.0741703	0.92530225	2.57523148
LOC400043	-1.49333394	-0.13032796	0.08727315	0.91362374	2.57523148
ASUN	-1.47858278	-0.0791382	0.05352301	0.94662294	2.57523148
UIMC1	-1.47546755	-0.08288662	0.05617651	0.94416662	2.57523148
KRT8	-1.46680594	-0.14734057	0.10044994	0.90291334	2.57523148
TOP2A	-1.45180472	-0.18123915	0.12483714	0.88194515	2.57523148
LOC647456	-1.42884638	-0.17556157	0.12286945	0.8854228	2.57523148
PLA1A	-1.4244843	-0.1651179	0.11591416	0.89185563	2.57523148
PPP6R1	-1.41044948	-0.07906443	0.05605619	0.94667135	2.57523148
HEXIM1	-1.40238877	-0.08975196	0.06399934	0.93968429	2.57523148
SNRPA1	-1.38960122	-0.07755457	0.05581067	0.94766262	2.57523148
SNRPD1	-1.38544171	-0.09697138	0.06999312	0.93499374	2.57523148
AKT1	-1.35275315	-0.06989014	0.05166511	0.95271055	4.62430056
TMEM158	-1.34291611	-0.17173386	0.1278813	0.88777509	4.62430056
CAPN13	-1.33277103	-0.16737205	0.125582	0.89046324	4.62430056
ATM	-1.31182021	-0.12951431	0.09872871	0.91413915	4.62430056
NUDCD1	-1.29878166	-0.13198861	0.10162494	0.91257269	4.62430056
S100A11	-1.28200992	-0.08129425	0.06341156	0.94520931	4.62430056
MRPS17	-1.26280494	-0.0673237	0.05331283	0.95440685	4.62430056
CDKN2B	-1.24563293	-0.11135669	0.08939768	0.92571712	4.62430056
MUC5B	-1.22641389	-0.21559498	0.17579301	0.86119092	4.62430056
LAG3	-1.22497418	-0.13464471	0.10991637	0.91089413	4.62430056
ACTL8	-1.21631275	-0.15863363	0.13042174	0.89587315	4.62430056
EPN3	-1.2123161	-0.13463041	0.11105223	0.91090316	4.62430056
CELSR1	-1.21060977	-0.12542205	0.10360238	0.91673582	4.62430056
ACTR3B	-1.20458231	-0.07578446	0.06291348	0.94882606	4.62430056
ITCH	-1.20428449	-0.06100037	0.05065279	0.9585992	4.62430056
MAPT	-1.20162793	-0.15804287	0.13152397	0.89624007	4.62430056
KRT6A	-1.18376372	-0.15614417	0.13190485	0.89742036	4.62430056
LOC389332	-1.16522295	-0.15989526	0.13722288	0.89509006	7.12291156
NOTCH3	-1.16471551	-0.07816114	0.0671075	0.94726426	7.12291156
GATA3	-1.16160666	-0.12789989	0.11010602	0.91516267	7.12291156
MPP1	-1.14246371	-0.10872355	0.09516586	0.92740824	7.12291156
USP10	-1.12364697	-0.05515127	0.04908238	0.96249352	7.12291156
TFF1	-1.11891959	-0.24853716	0.22212245	0.84174949	7.12291156
NEK2	-1.1181577	-0.1491111	0.13335427	0.90180593	7.12291156
SMO	-1.11021482	-0.08943693	0.08055822	0.93988951	7.12291156
GSTM1	-1.10845977	-0.1261169	0.11377671	0.91629439	7.12291156
AXL	-1.09277785	-0.08088497	0.07401776	0.9454775	7.12291156
CABP7	-1.07301703	-0.14408333	0.1342787	0.90495419	7.12291156
NCS1	-1.06671083	-0.08085802	0.07580126	0.94549516	7.12291156
NFKBIB	-1.0450364	-0.06642106	0.06355861	0.95500417	7.12291156
TMCC2	-1.03674498	-0.10708948	0.10329395	0.92845926	7.12291156
EPCAM	-1.03320772	-0.11155717	0.10797168	0.92558849	7.12291156

SLC9A3R1	-1.02798462	-0.11479547	0.11167042	0.92351323	7.12291156
NUP88	-1.02774595	-0.05483908	0.0533586	0.96270182	7.12291156
GPSM2	-1.01299968	-0.07812544	0.07712287	0.9472877	7.12291156
SEH1L	-1.01084786	-0.0477244	0.04721225	0.96746113	7.12291156
TFF3	-1.00109244	-0.19019274	0.18998519	0.87648862	7.12291156
AVEN	-0.96177055	-0.05530719	0.0575056	0.9623895	7.12291156
REEP6	-0.96095276	-0.10462435	0.10887564	0.93004708	7.12291156
STK11	-0.95003958	-0.04943076	0.05203021	0.96631753	10.1783653
MSH2	-0.93863512	-0.05425729	0.05780446	0.96309012	10.1783653
RELB	-0.93584268	-0.06567293	0.07017518	0.95549953	10.1783653
C1orf106	-0.93219669	-0.11506786	0.12343732	0.92333888	10.1783653
ITGB1	-0.91544208	-0.05326086	0.05818048	0.96375553	10.1783653
FANK1	-0.91275959	-0.08001912	0.08766725	0.94604511	10.1783653
AVL9	-0.91066797	-0.04852286	0.05328271	0.96692583	10.1783653
SLC9A3	-0.90848104	-0.09997009	0.11004092	0.93305234	10.1783653
KRT18	-0.90800139	-0.09155568	0.10083209	0.93851019	10.1783653
RFC4	-0.90163347	-0.0603641	0.06694971	0.95902206	10.1783653
S100A9	-0.89328855	-0.1500102	0.16793028	0.90124409	10.1783653
DDR1	-0.87193189	-0.07653116	0.08777195	0.9483351	10.1783653
BDNF	-0.86684258	-0.09159471	0.10566476	0.9384848	10.1783653
FAP	-0.85208062	-0.0865339	0.101556	0.94178268	10.1783653
CAMK2N1	-0.85163236	-0.07707139	0.09049843	0.94798005	10.1783653
NPM2	-0.83147948	-0.09737239	0.11710739	0.93473389	10.1783653
PITX1	-0.82362247	-0.10697447	0.1298829	0.92853328	10.1783653
NLN	-0.81651221	-0.04788629	0.05864736	0.96735257	10.1783653
TFRC	-0.800538	-0.06662121	0.08322055	0.95487169	10.1783653
PCDH8	-0.78561829	-0.10899285	0.13873513	0.92723514	10.1783653
TMEM25	-0.78169355	-0.06276325	0.08029137	0.95742856	10.1783653
ST18	-0.76149832	-0.09249162	0.12146005	0.93790154	13.1939519
SQLE	-0.73082516	-0.06710028	0.09181442	0.95455466	13.1939519
TRIP13	-0.71097662	-0.06893486	0.09695798	0.95334159	13.1939519
ERCC1	-0.7078308	-0.04955725	0.07001285	0.96623281	13.1939519
EMP3	-0.70638563	-0.05972879	0.0845555	0.95944447	13.1939519
LAMC2	-0.70028673	-0.0809511	0.11559708	0.94543416	13.1939519
KRT16	-0.68916757	-0.09544666	0.13849557	0.93598242	13.1939519
MIEN1	-0.68744491	-0.0383446	0.05577843	0.97377165	13.1939519
SHC1	-0.66464317	-0.03391862	0.05103283	0.97676362	13.1939519
CCND2	-0.66223376	-0.06348316	0.09586217	0.95695092	13.1939519
KRTAP1.1	-0.64476747	-0.08237217	0.12775485	0.94450335	13.1939519
OCLN	-0.63523833	-0.06329859	0.09964541	0.95707336	13.1939519
TUBB6	-0.63289384	-0.04533905	0.07163768	0.96906205	13.1939519
RRP15	-0.63250253	-0.04240682	0.06704609	0.97103364	13.1939519
CCND1	-0.60780031	-0.06391799	0.10516281	0.95666254	13.1939519
GUSB	-0.59909872	-0.04548139	0.07591635	0.96896645	13.1939519
SPINT2	-0.59511997	-0.04583351	0.07701557	0.96872998	16.5221765
NPEPPS	-0.59290721	-0.03179022	0.05361753	0.9782057	16.5221765
IDO1	-0.58855932	-0.08511942	0.14462335	0.9427065	16.5221765
FGFR4	-0.58557922	-0.07552202	0.12896977	0.94899868	16.5221765
FGFR2	-0.5841861	-0.06162358	0.10548622	0.95818519	16.5221765
KCNJ15	-0.58327692	-0.06054578	0.1038028	0.9589013	16.5221765

CXCR2	-0.5368756	-0.06603637	0.12300125	0.95525886	16.5221765
LSR	-0.51855321	-0.04033627	0.07778618	0.97242826	16.5221765
DEGS2	-0.51309167	-0.06177531	0.1203982	0.95808442	16.5221765
RB1	-0.50800875	-0.02874133	0.05657644	0.98027516	16.5221765
ARAF	-0.45777834	-0.02520289	0.05505478	0.98268239	16.5221765
CFLAR	-0.45379845	-0.03163637	0.06971459	0.97831002	16.5221765
MYO5C	-0.42035289	-0.03418679	0.08132879	0.97658208	19.8526936
TOR1A	-0.41603087	-0.01865489	0.04484017	0.98715265	19.8526936
HMGA1	-0.41311808	-0.03790183	0.09174576	0.97407055	19.8526936
KRT19	-0.41249452	-0.04952751	0.12006828	0.96625273	19.8526936
UCHL1	-0.39617762	-0.04506674	0.11375388	0.96924498	19.8526936
MIS18A	-0.38389183	-0.02368948	0.06170873	0.98371378	19.8526936
IL6R	-0.37119053	-0.03225311	0.08689098	0.97789189	19.8526936
FAM171A1	-0.36462616	-0.03288467	0.09018736	0.9774639	19.8526936
SPDEF	-0.3617745	-0.0429442	0.11870433	0.97067201	19.8526936
P4HTM	-0.34213233	-0.02294415	0.06706221	0.98422212	19.8526936
GRHL2	-0.31990039	-0.03113981	0.09734219	0.97864681	19.8526936
CKS2	-0.27968963	-0.02570618	0.09190967	0.98233964	19.8526936
SNAI1	-0.27710332	-0.02668677	0.0963062	0.98167218	19.8526936
ESRP1	-0.27182392	-0.02649654	0.09747684	0.98180163	19.8526936
CDK4	-0.26040271	-0.01376183	0.05284827	0.99050638	19.8526936
ERBB3	-0.25226232	-0.02303192	0.09130147	0.98416225	19.8526936
ELSPBP1	-0.23018232	-0.02655589	0.11536892	0.98176124	19.8526936
NF1	-0.21872151	-0.01224306	0.05597556	0.99154966	19.8526936
INPP4B	-0.21099802	-0.01909787	0.09051208	0.9868496	19.8526936
GGH	-0.19523251	-0.01652647	0.0846502	0.98861008	19.8526936
KRT6C	-0.18210268	-0.02689322	0.14768163	0.98153171	19.8526936
RAD51C	-0.18120884	-0.01062289	0.05862238	0.99266381	19.8526936
RAB35	-0.16780591	-0.00741632	0.0441958	0.99487259	19.8526936
LRRC2	-0.15570594	-0.01728276	0.11099614	0.98809197	19.8526936
GREM1	-0.15258498	-0.01957341	0.12827873	0.98652437	19.8526936
CDH3	-0.13286235	-0.0145132	0.10923485	0.98999065	19.8526936
FAM174B	-0.12850656	-0.01192506	0.0927973	0.99176824	19.8526936
ASF1A	-0.11539066	-0.00616045	0.05338775	0.99573901	19.8526936
NFKB1	-0.09220897	-0.00594078	0.06442736	0.99589063	19.8526936
KIF20A	-0.08806536	-0.01101116	0.1250339	0.9923967	19.8526936
KIAA0040	-0.05033126	-0.00405535	0.08057319	0.99719299	19.8526936
GABPB1	-0.04576892	-0.00229609	0.05016703	0.99840974	19.8526936
ABCC8	-0.04066384	-0.00486542	0.11964971	0.99663323	19.8526936
S100A8	-0.02838317	-0.00467675	0.16477189	0.99676357	19.8526936
BLVRA	-0.0210903	-0.00145303	0.06889564	0.99899334	19.8526936
VAV3	-0.00741953	-0.0006427	0.08662258	0.99955461	19.8526936
PIR	-0.00671612	-0.00063782	0.09496836	0.999558	19.8526936
CMC2	-0.00259408	-0.00014609	0.05631557	0.99989875	19.8526936
