# Supplementary material for: Neoadjuvant eribulin in HER2-negative early-stage breast cancer (SOLTI-1007-NeoEribulin): a multicenter, two-cohort, non-randomized phase II trial
Source: NPJ Breast Cancer. 2021 Nov 25;7:145. doi: 10.1038/s41523-021-00351-4 (PMC8616926; doi:10.1038/s41523-021-00351-4)
Supplement: Supplementary file 2 — Reporting Summary [file 41523_2021_351_MOESM2_ESM.pdf]

nature portfolio

Corresponding author(s): Aleix Prat, MD, PhD

Last updated by author(s): Oct 6, 2021

Reporting Summary

Nature Portfolio wishes to improve the reproducibility of the work that we publish. This form provides structure for consistency and transparency in reporting. For further information on Nature Portfolio policies, see our Editorial Policies and the Editorial Policy Checklist.

Statistics

For all statistical analyses, confirm that the following items are present in the figure legend, table legend, main text, or Methods section.

n/a	Confirmed	
☐	☒	The exact sample size (n) for each experimental group/condition, given as a discrete number and unit of measurement
☐	☒	A statement on whether measurements were taken from distinct samples or whether the same sample was measured repeatedly
☐	☒	The statistical test(s) used AND whether they are one- or two-sided<br>*Only common tests should be described solely by name; describe more complex techniques in the Methods section.*
☐	☒	A description of all covariates tested
☐	☒	A description of any assumptions or corrections, such as tests of normality and adjustment for multiple comparisons
☐	☒	A full description of the statistical parameters including central tendency (e.g. means) or other basic estimates (e.g. regression coefficient) AND variation (e.g. standard deviation) or associated estimates of uncertainty (e.g. confidence intervals)
☐	☒	For null hypothesis testing, the test statistic (e.g. F, t, r) with confidence intervals, effect sizes, degrees of freedom and P value noted<br>*Give P values as exact values whenever suitable.*
☒	☐	For Bayesian analysis, information on the choice of priors and Markov chain Monte Carlo settings
☒	☐	For hierarchical and complex designs, identification of the appropriate level for tests and full reporting of outcomes
☐	☒	Estimates of effect sizes (e.g. Cohen's d, Pearson's r), indicating how they were calculated

Our web collection on statistics for biologists contains articles on many of the points above.

Software and code

Policy information about availability of computer code

Data collection	Genomic analysis and TILs determination were made available without associated clinical data
Data analysis	All analyses were performed with R version 3.6.1 and Microsoft Excel.

For manuscripts utilizing custom algorithms or software that are central to the research but not yet described in published literature, software must be made available to editors and reviewers. We strongly encourage code deposition in a community repository (e.g. GitHub). See the Nature Portfolio guidelines for submitting code & software for further information.

Data

Policy information about availability of data

All manuscripts must include a data availability statement. This statement should provide the following information, where applicable:

- Accession codes, unique identifiers, or web links for publicly available datasets
- A description of any restrictions on data availability
- For clinical datasets or third party data, please ensure that the statement adheres to our policy

All datasets used and/or analyzed during the current study are available from the corresponding author on reasonable request.

Field-specific reporting

Please select the one below that is the best fit for your research. If you are not sure, read the appropriate sections before making your selection.

☒ Life sciences ☐ Behavioural & social sciences ☐ Ecological, evolutionary & environmental sciences

For a reference copy of the document with all sections, see nature.com/documents/nr-reporting-summary-flat.pdf

Life sciences study design

All studies must disclose on these points even when the disclosure is negative.

Sample size	This trial was an exploratory study and sample size was not based on statistical power. Assuming an average pCRB of 15% based on the literature31, a sample size of 200 patients, 100 TNBC patients and 100 HR-positive, was estimated to provide a 90% probability of detecting a gene signature whose expression is so associated with a two-fold increase in odds of achieving a pCRB, assuming 5% of patients were lost to follow-up and 5% had insufficient quality or quantity of RNA.
Data exclusions	For genomic analyses genes with more than 80% missing data were excluded. IN all other cases patients were excluded only if data were missing.
Replication	No replications were undertaken
Randomization	The study was not randomized, since it was phase II single arm.
Blinding	The study was not blinded, since it was phase II single arm.

Reporting for specific materials, systems and methods

We require information from authors about some types of materials, experimental systems and methods used in many studies. Here, indicate whether each material, system or method listed is relevant to your study. If you are not sure if a list item applies to your research, read the appropriate section before selecting a response.

Materials & experimental systems

n/a	Involved in the study	
☒	☐	Antibodies
☒	☐	Eukaryotic cell lines
☒	☐	Palaeontology and archaeology
☒	☐	Animals and other organisms
☐	☒	Human research participants
☐	☒	Clinical data
☒	☐	Dual use research of concern

Methods

n/a	Involved in the study	
☒	☐	ChIP-seq
☒	☐	Flow cytometry
☒	☐	MRI-based neuroimaging

Human research participants

Policy information about studies involving human research participants

Population characteristics	Patients aged at least 18 years were eligible if they had previously untreated, locally confirmed HER2-negative, stage I–II invasive breast cancer (regardless of hormone receptor status), with primary tumors at least 2 cm in diameter (as measured by ultrasound or MRI), nodal status of 0–2, and no evidence of distant metastasis. Patients had to meet the minimum tissue requirement for gene expression analysis (≥10% invasive tumor cells and >4 mm2 tumor surface area). Patients also had to have an Eastern Cooperative Oncology Group (ECOG) performance status of 0-1, and adequate haematological counts and hepatic and renal function. Patients were excluded if they had multicentric tumors, stage III or IV disease, bilateral breast cancer, other malignancies, inadequate bone marrow or renal function, impaired liver or cardiac function, clinically significant cardiovascular disease, and uncontrolled infection.
Recruitment	SOLTI-1007 NeoEribulin is an open-label, two cohort, conducted in 3 countries at 30 trial centers, phase 2 pharmacogenomic study of single agent eribulin as neoadjuvant treatment for operable Stage I-II HER2-negative breast cancer. Cohort 1 included patients with TNBC and cohort 2 included HR-positive breast cancer. Between September 2012 and October 2015, 174 patients were enrolled (73 TNBC patients and 101 HR-positive patients)
Ethics oversight	This study was approved by the Institutional Review Board of Hospital Vall d'Hebron (Barcelona). All patients provided written informed consent.

Note that full information on the approval of the study protocol must also be provided in the manuscript.

Clinical data

Policy information about clinical studies

All manuscripts should comply with the ICMJE guidelines for publication of clinical research and a completed CONSORT checklist must be included with all submissions.

Clinical trial registration	NCT01669252
Study protocol	https://clinicaltrials.gov/ct2/show/NCT01669252
Data collection	SOLTI-1007 NeoEribulin is an open-label, two cohort, conducted in 3 countries at 30 trial centers. Between September 2012 and October 2015, 174 patients were enrolled
Outcomes	The primary endpoint of the trial was the correlation of pre-treatment expression of mRNA from primary breast tumors with pathological complete response in the breast (pCRB) —defined as the absence of invasive neoplastic cells at microscopic examination of the primary tumor—at the time of surgery. Remaining in-situ lesions were allowed.<br>The seven key secondary endpoints that we report in this article are: (1) proportion of patients with a pCRB (ypT0/Tis ypNx) and pCR in breast and axillary lymph nodes (pCRBL; defined as ypT0/Tis ypN0).(2) proportion of patients who had an objective response (defined as the sum of partial responses and complete responses according to Response Evaluation Criteria in Solid Tumors, version 1.1) (3) proportion of patients with a residual cancer burden score (RCB) 0-1; (4) frequency of breast conserving surgery; (5) safety and tolerability of treatment (6) proportion of pCRB according to breast intrinsic cancer subtype (7) proportion of subtype switching from baseline to Cycle2 Day1 and surgery.
